# Supplementary material for: Towards Antibacterial Agents: Synthesis and Biological Activity of Multivalent Amide Derivatives of Thiacalix[4]arene with Hydroxyl and Amine Groups
Source: Pharmaceutics. 2023 Dec 5;15(12):2731. doi: 10.3390/pharmaceutics15122731 (PMC10747887; doi:10.3390/pharmaceutics15122731)
Supplement: Supplementary file 1 [file pharmaceutics-15-02731-s001.zip › pharmaceutics-2739559-supplementary.pdf]

Supplementary Information

Towards Antibacterial Agents: Synthesis and Biological Activity of Multivalent Amide Derivatives of Thiacalix[4]arene with Hydroxyl and Amine Groups

Igor Shiabiev [1], Dmitry Pysin [1], Alan Akhmedov [1], Olga Babaeva [2], Vasily Babaev [2], Anna Lyubina [2], Alexandra Voloshina [2], Konstantin Petrov [2], Pavel Padnya [1,*] and Ivan Stoikov [1,*]

[1] A.M. Butlerov Chemical Institute, Kazan Federal University, Kremlevskaya, 18, Kazan 420008, Russia; shiabiev.ig@yandex.ru (I.S.); pysin_dima@mail.ru (D.P.); naive2294@gmail.com (A.A.)

[2] Arbuzov Institute of Organic and Physical Chemistry, FRC Kazan Scientific Center, Russian Academy of Sciences, 8 Arbuzov Street, Kazan 420088, Russia; olbazanova@iopc.ru (O.B.); babaev@iopc.ru (V.B.); aplyubina@gmail.com (A.L.); sobaka-1968@mail.ru (A.V.); kpetrov2005@mail.ru (K.P.)

* Correspondence: padnya.ksu@gmail.com (P.P.); ivan.stoikov@mail.ru (I.S.); Tel.: +7-843-233-7463 (I.S.)

1. NMR, IR, and mass spectra of the synthesized compounds ______ 2

2. Biological assay ______ 41

3. DLS data ______ 42

4. Table S3. Values (MW, miLogP, HBA, HBD, TPSA) and solubility data for compounds 7–18 and 21–24 ______ 44

1. NMR, IR, and mass spectra of the synthesized compounds

^1H NMR and $^{13}C\{^1H\}$ NMR NMR spectra were obtained on the Bruker Avance-400 spectrometer (Bruker Corp., Billerica, MA, USA) (^1H 400 MHz and $^{13}C\{^1H\}$ 100 MHz). Chemical shifts were determined against the signals of residual protons of deuterated solvent (DMSO-d_6, $CDCl_3$, and CD_3OD). Concentrations of the compounds were equal to 3–5 mass %. FTIR ATR spectra were recorded on the Spectrum 400 FT-IR spectrometer (Perkin–Elmer, Seer Green, Llantrisant, UK) with the Diamond KRS-5 attenuated total internal reflectance attachment (resolution 0.5 cm^{-1}, accumulation of 64 scans, recording time 16 s in the wavelength range 400–4000 cm^{-1}). Melting points were determined using the Boetius Block apparatus (VEB Kombinat Nagema, Radebeul, Germany). ESI-HRMS experiments of compounds **4**–**6** were performed at Agilent 6550 iFunnel Q-TOF LC/MS (Agilent Technologies, Santa Clara, CA, USA), equipped with Agilent 1290 Infinity II LC. For compounds **7**–**18**, samples were analyzed using Impact II mass spectrometer with Elute UHPLC system («Bruker Daltonik GmbH», Germany). The column used was YMC-Triart C18 (50×2 mm; 3 μm). The temperature of the column thermostat was set at 40 °C and the temperature of the autosampler at 12 °C. Elution solvents used were 0.1% formic acid in Milli-Q water (A) and 0.1% formic acid in HPLC-grade acetonitrile (B) and elution gradient was the following: 0 min at 5% B, 3 min at 95% B, 4 min at 95% B, 4.1 min at 95% B, 6 min at 95% B with flow rate of 0.3 mL/min. The injection volume was 2 μL. Analytes were ionized by electrospray in positive polarity. ESI conditions were set with the capillary temperature at 220 °C, capillary voltage at 4.5 kV and sheath gas (N_2) flow rate of 6 L/min. Measurements were made in the range m/z 300-3500. The solution of analyte (1 mg/mL, HPLC-grade methanol) was diluted in HPLC-grade acetonitrile to concentration 0.01 mg/mL. The solution of sodium iodide in Milli-Q water (0.2 mg/mL) was used as calibrant. The relative error in determining the masses was no more than 5.0 ppm. For instrument control and data acquiring the otofControl software (Bruker Daltonik GmbH, Version 5.2) was used. Data processing was performed by DataAnalysis software (Bruker Daltonik GmbH, Version 5.3).

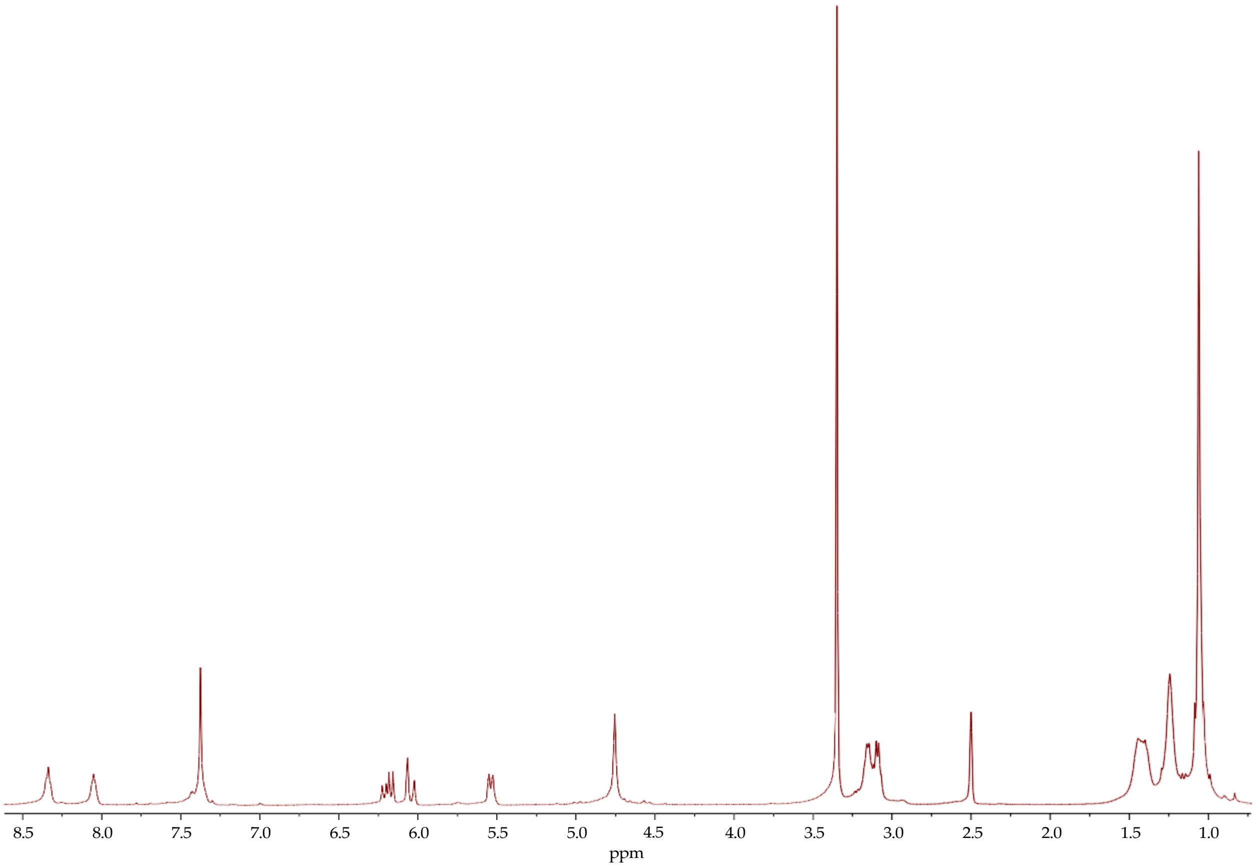

Figure S1. ^{1}H NMR spectrum of **4**, DMSO-*d*$_6$, 298 K, 400 MHz

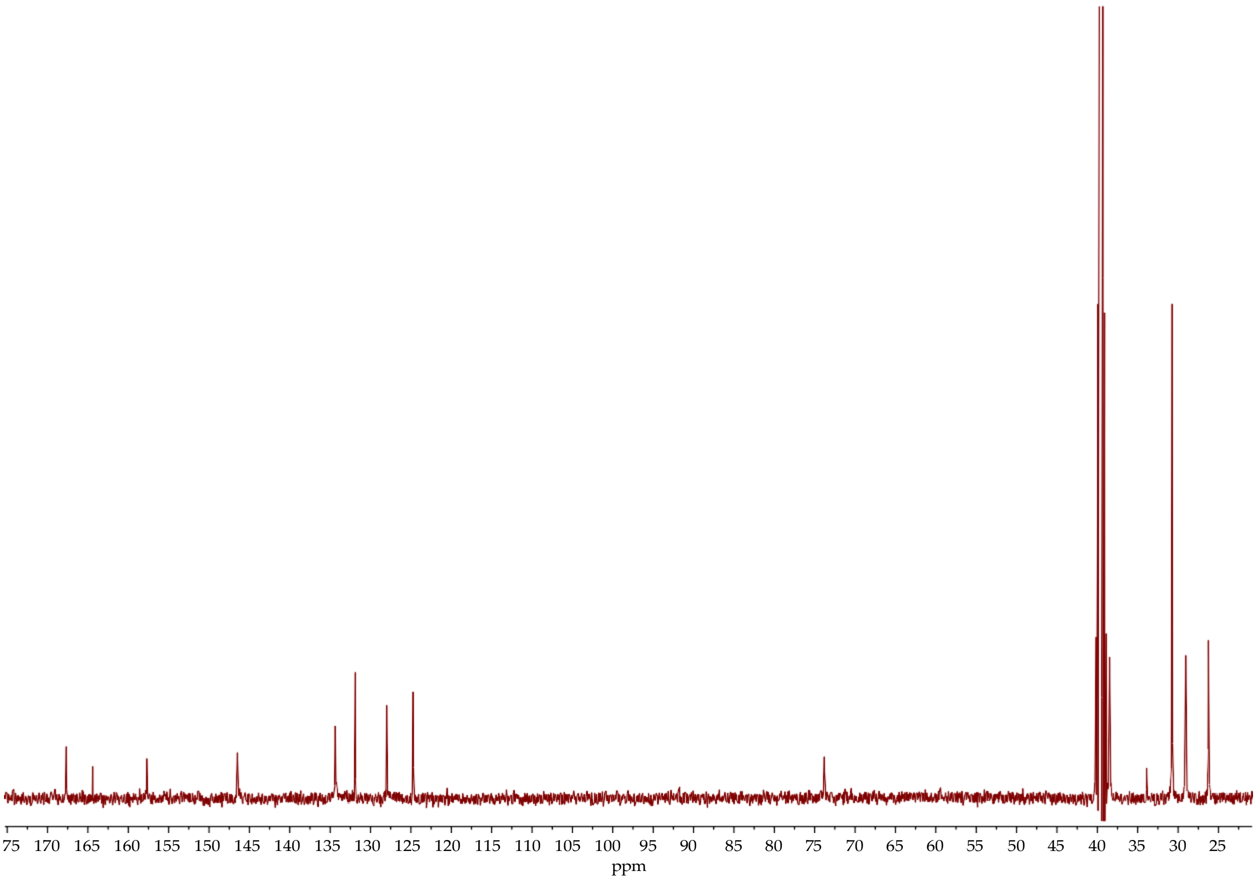

Figure S2. $^{13}C\{^{1}H\}$ NMR spectrum of **4**, DMSO-*d*$_6$, 298 K, 100 MHz

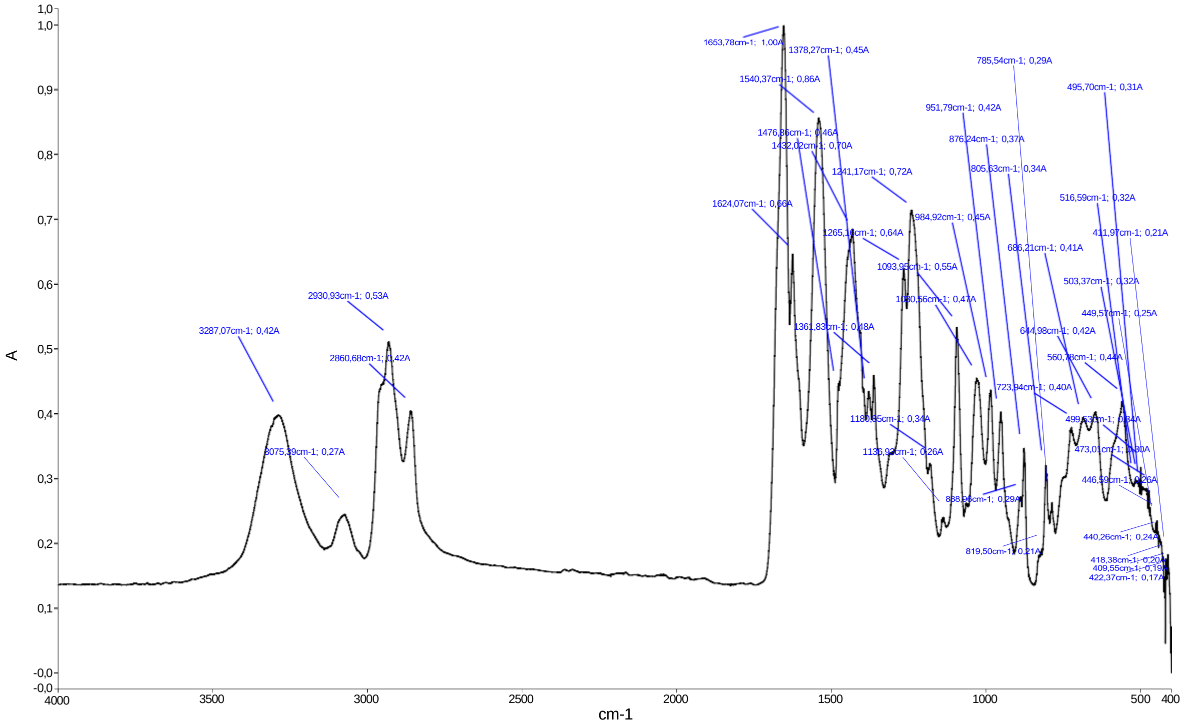

Figure S3. FTIR-ATR spectrum of **4**

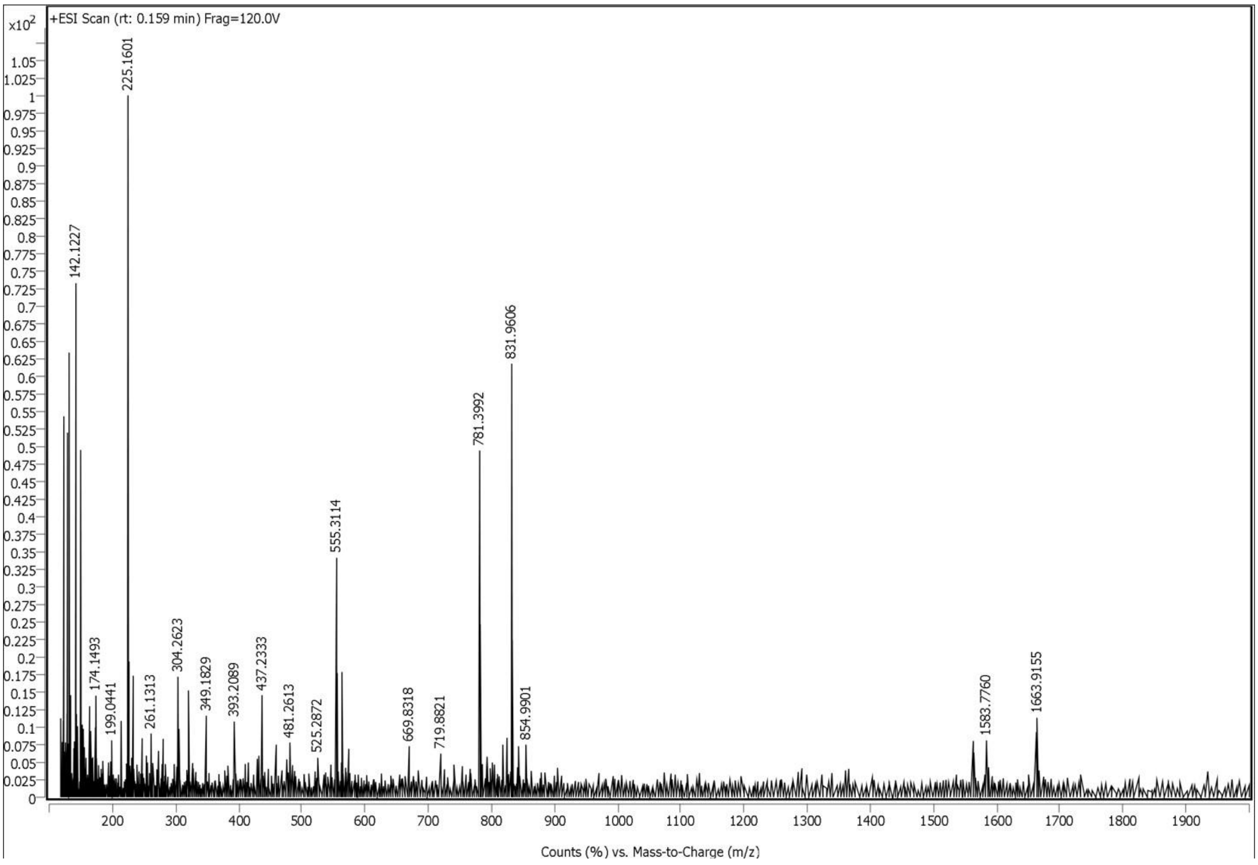

Figure S4. Mass spectrum (HR ESI) of **4**

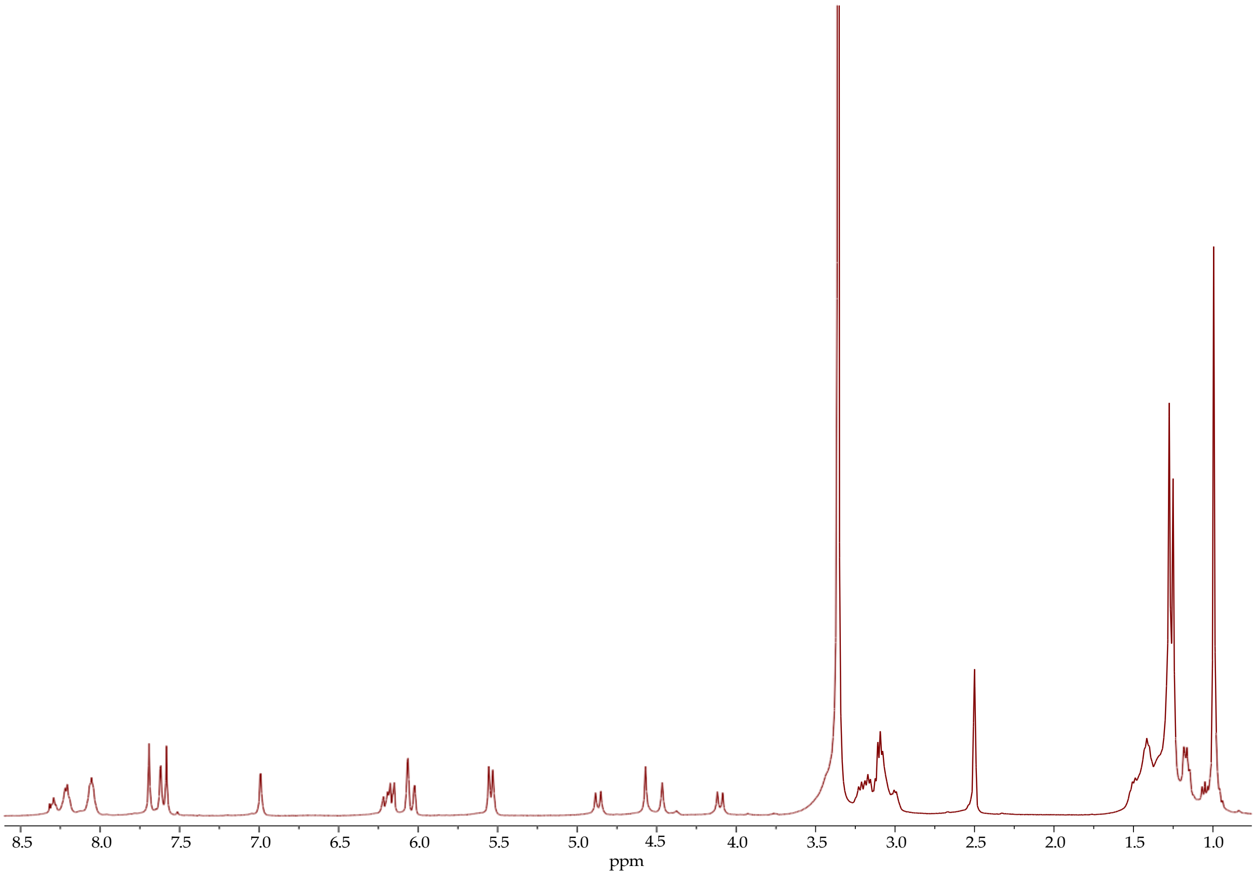

Figure S5. ^{1}H NMR spectrum of **5**, DMSO-d_6, 298 K, 400 MHz

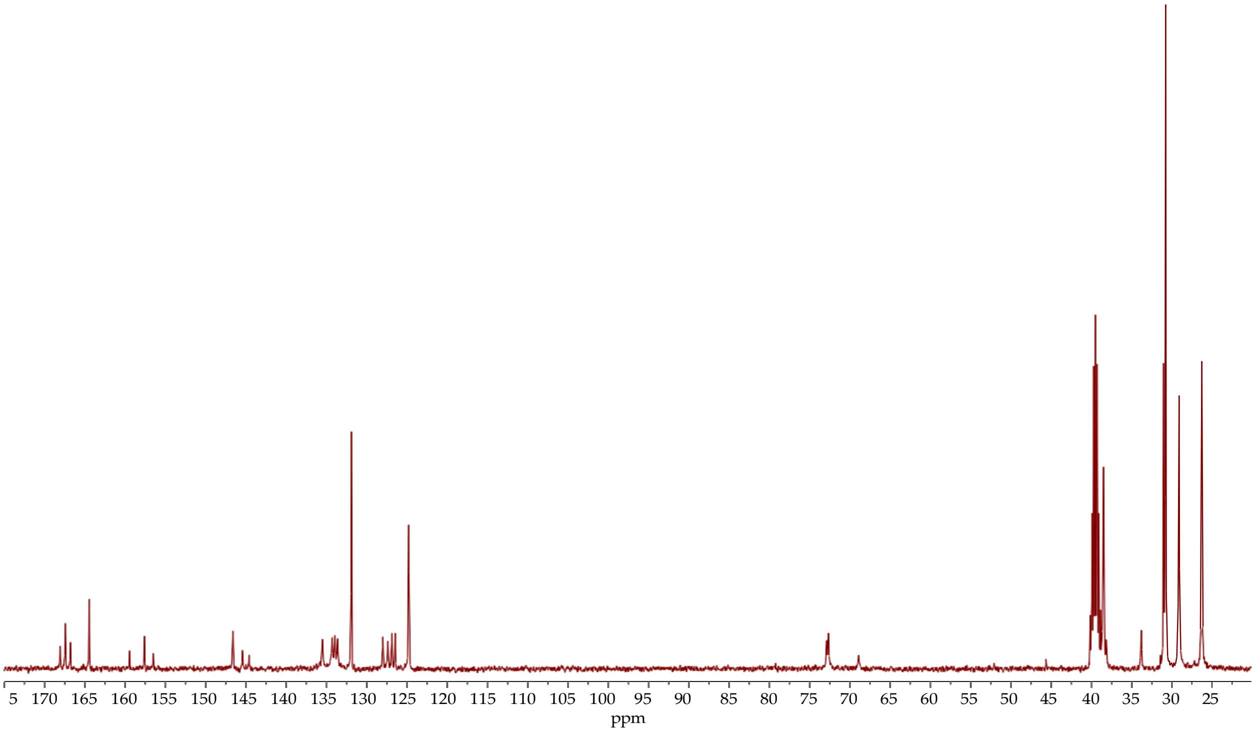

Figure S6. $^{13}C\{^{1}H\}$ NMR spectrum of **5**, DMSO-d_6, 298 K, 100 MHz

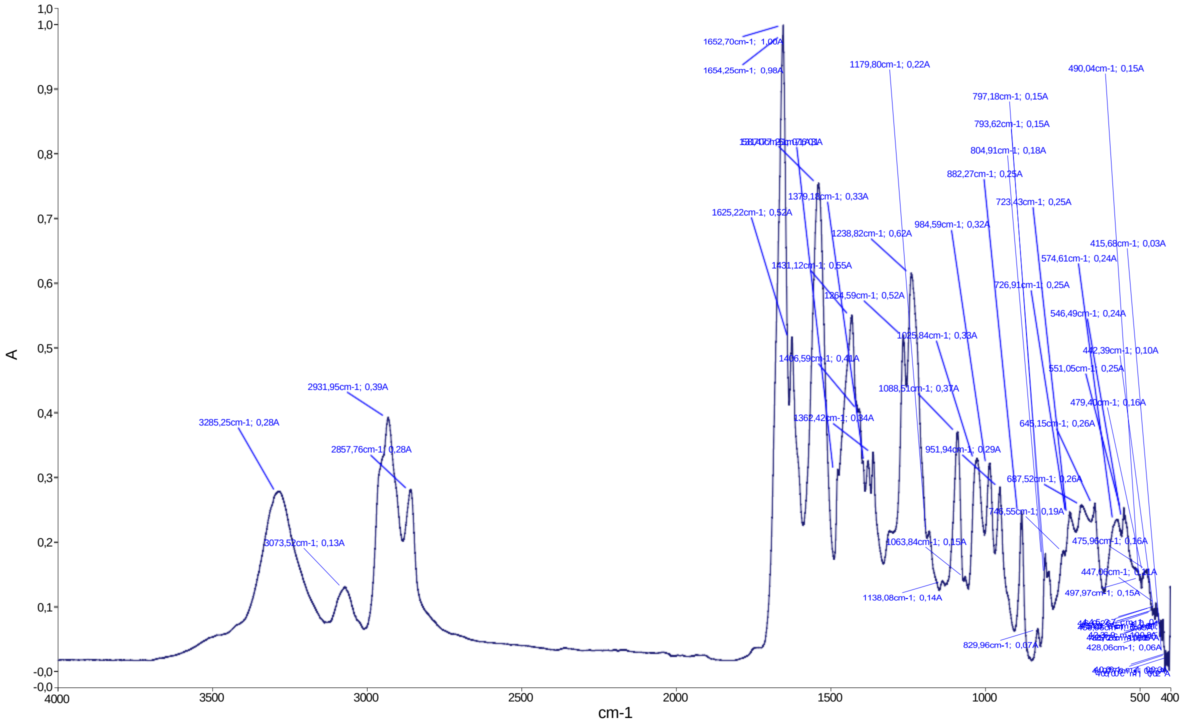

Figure S7. FTIR-ATR spectrum of **5**

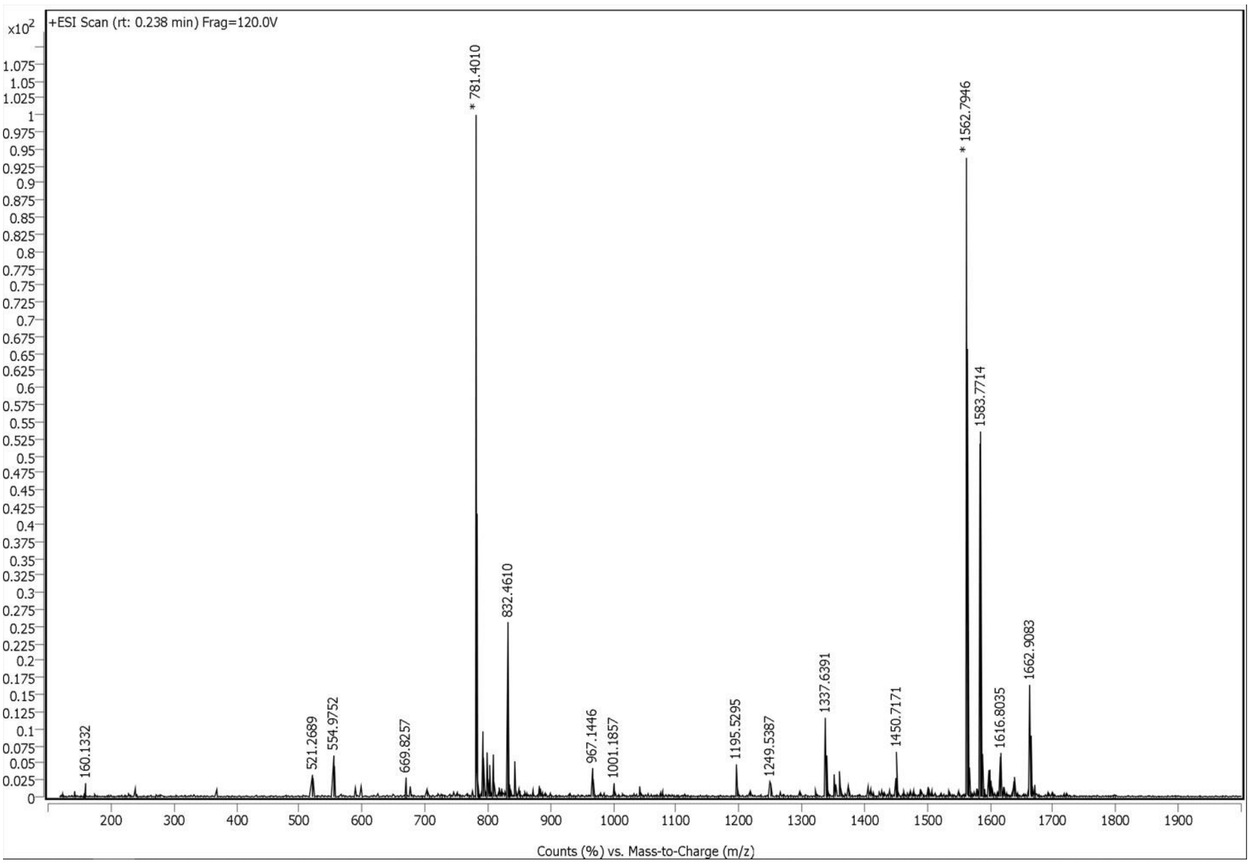

Figure S8. Mass spectrum (HR ESI) of **5**

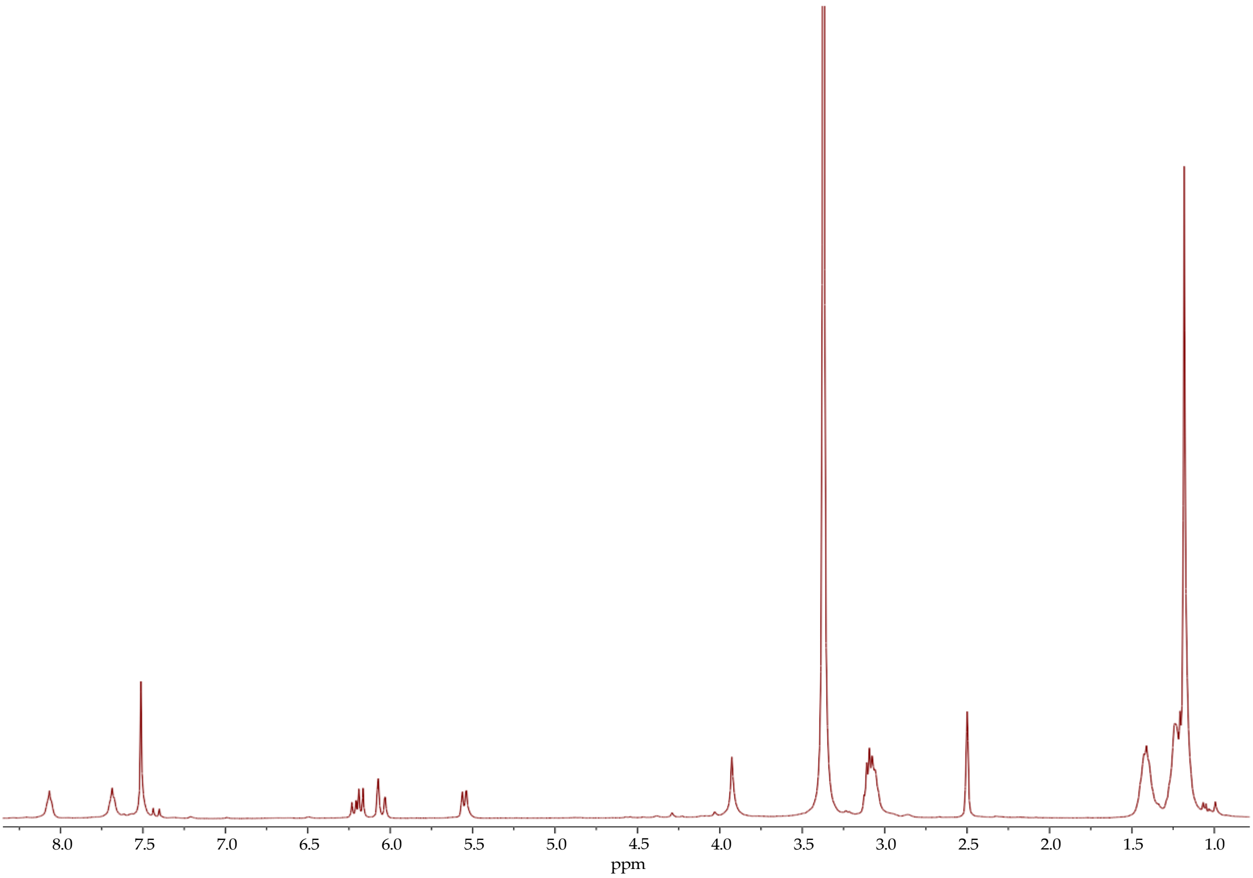

Figure S9. ^{1}H NMR spectrum of **6**, DMSO-d_6, 298 K, 400 MHz

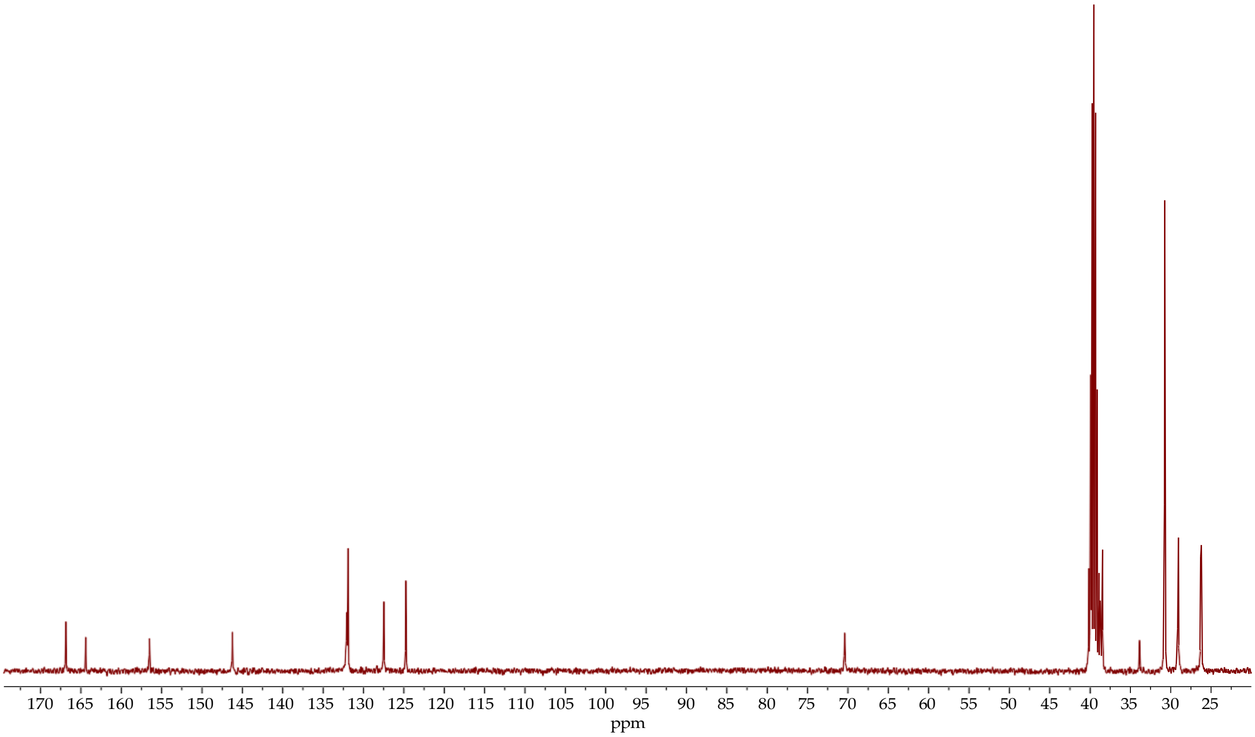

Figure S10. $^{13}C\{^{1}H\}$ NMR spectrum of **6**, DMSO-d_6, 298 K, 100 MHz

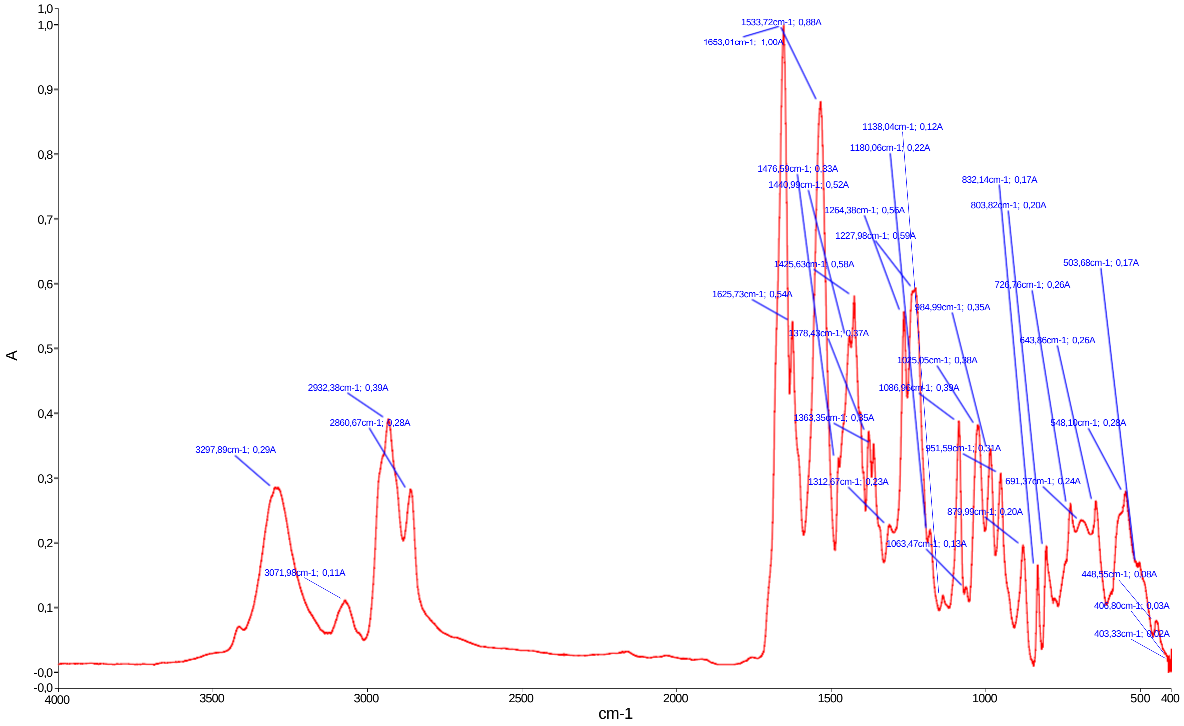

Figure S11. FTIR-ATR spectrum of **6**

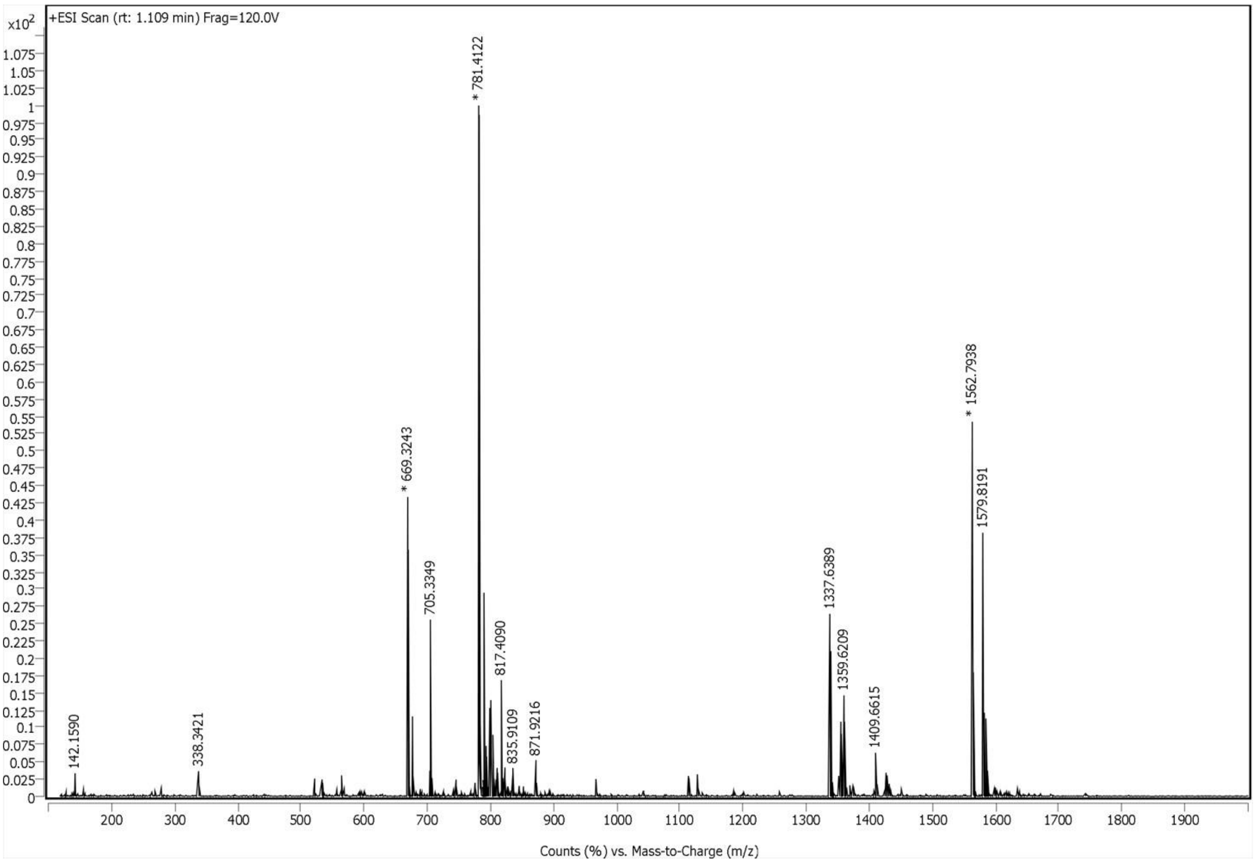

Figure S12. Mass spectrum (HR ESI) of **6**

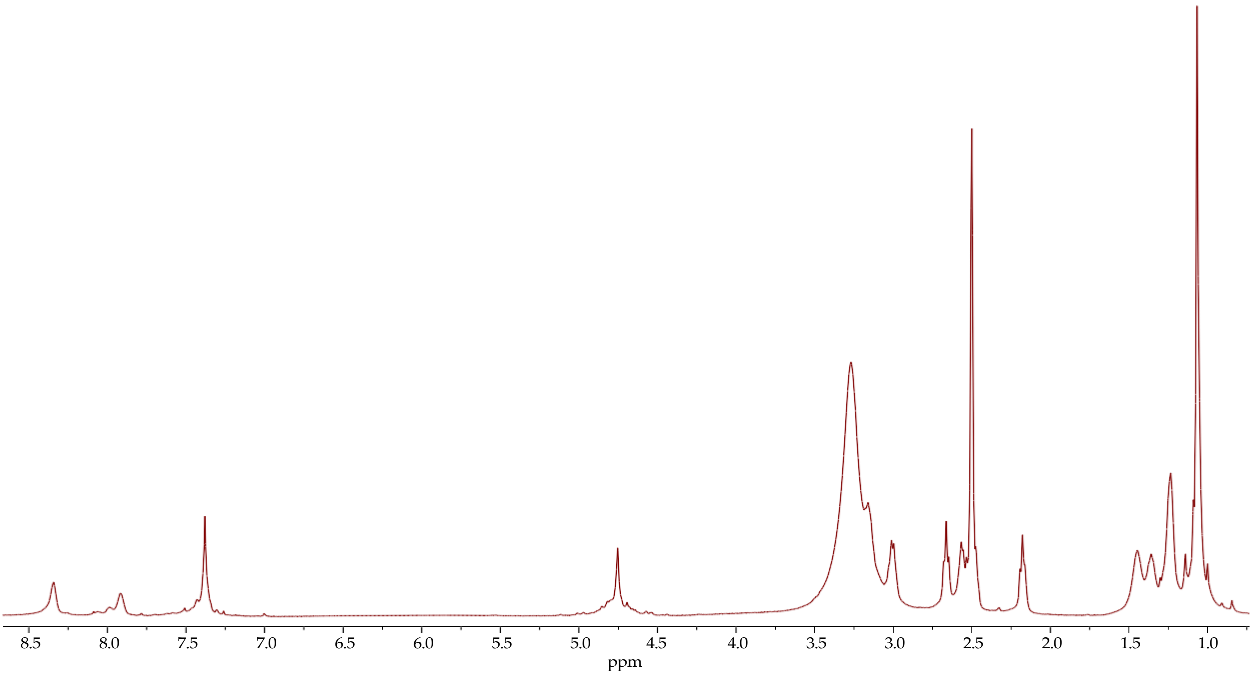

Figure S13. ^{1}H NMR spectrum of **7**, DMSO-d_6, 298 K, 400 MHz

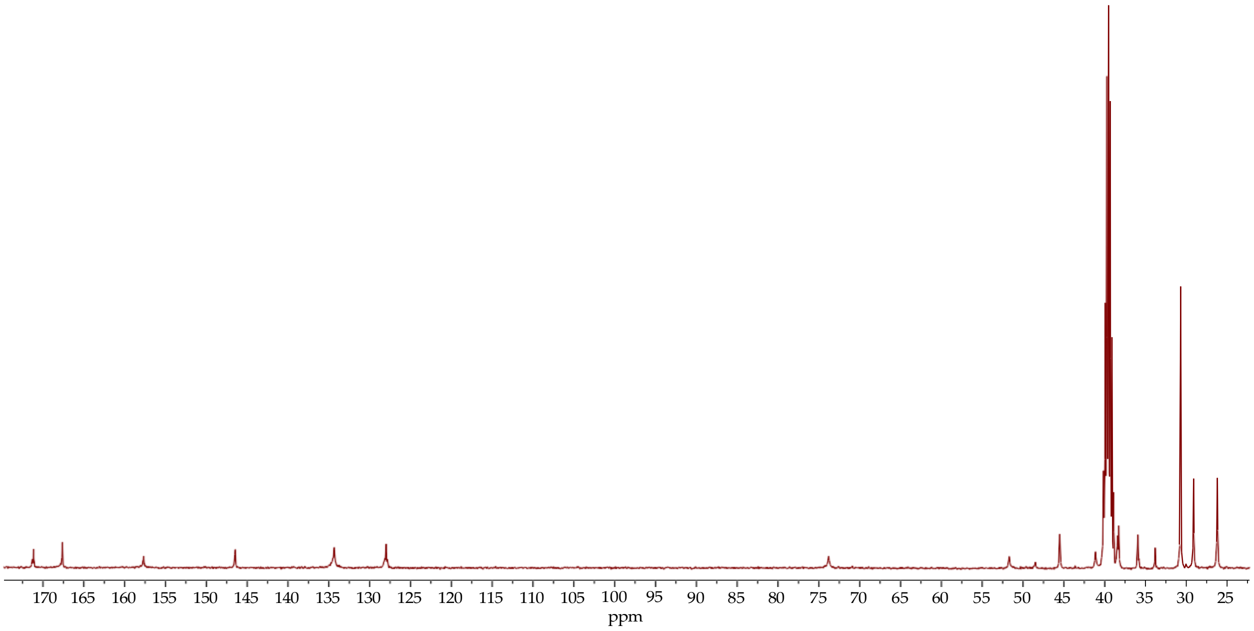

Figure S14. $^{13}C\{^{1}H\}$ NMR spectrum of **7**, DMSO-d_6, 298 K, 100 MHz

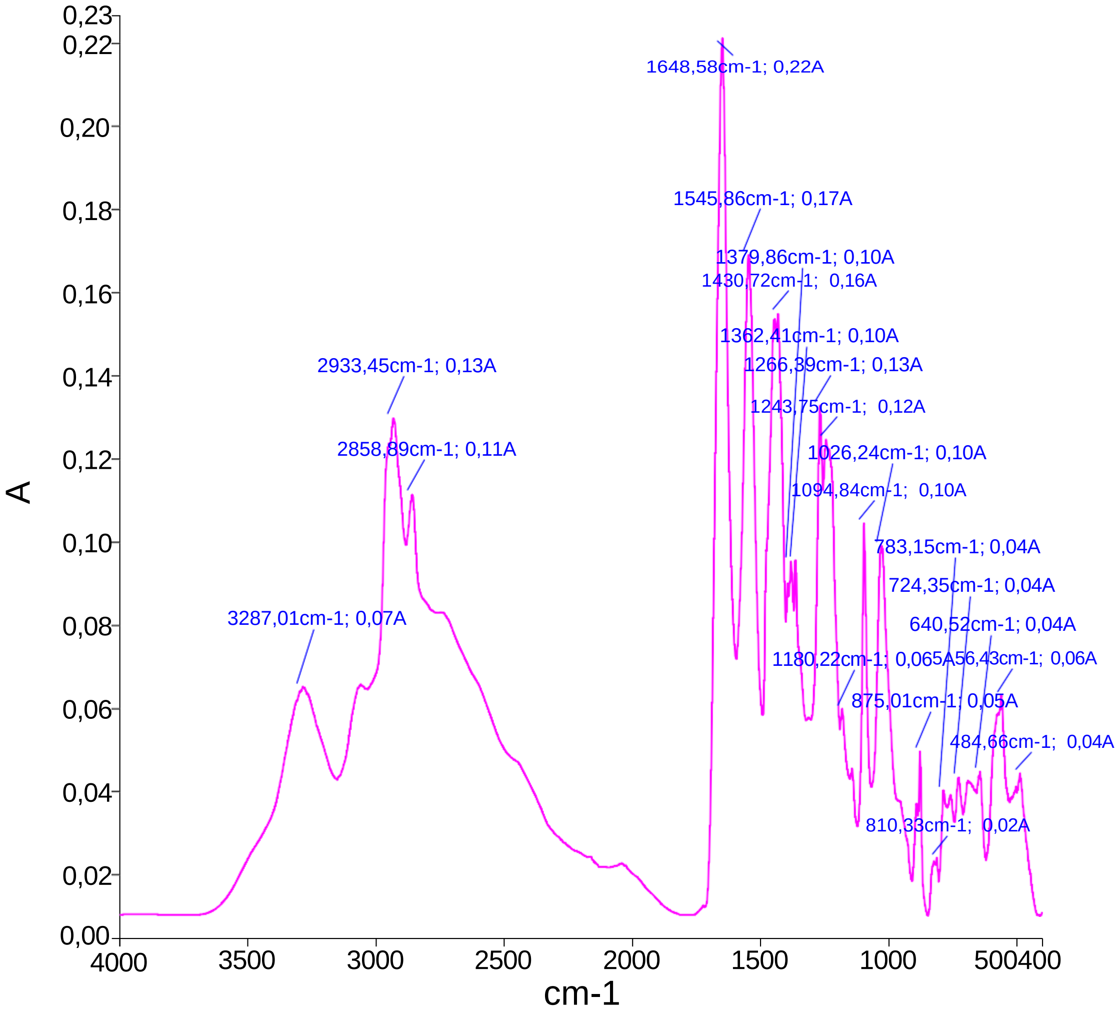

Figure S15. FTIR-ATR spectrum of **7**

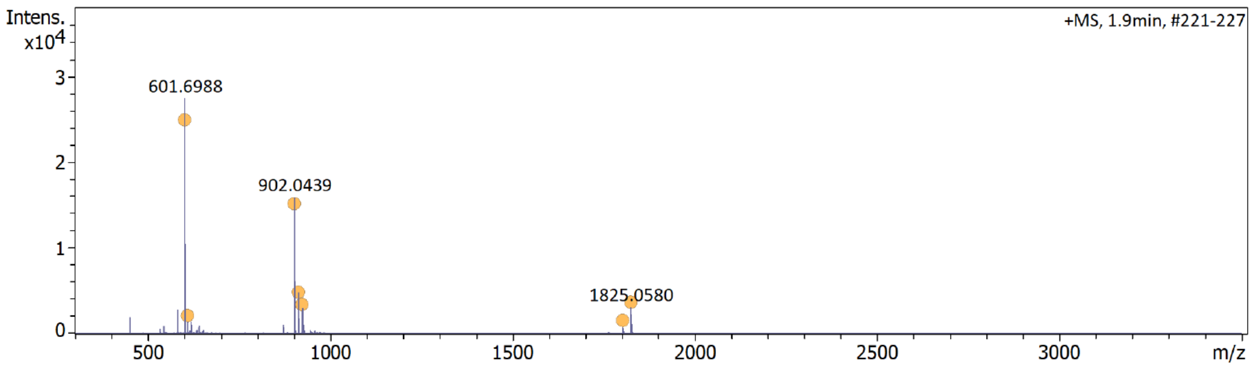

Figure S16. Mass spectrum (HR ESI) of **7**

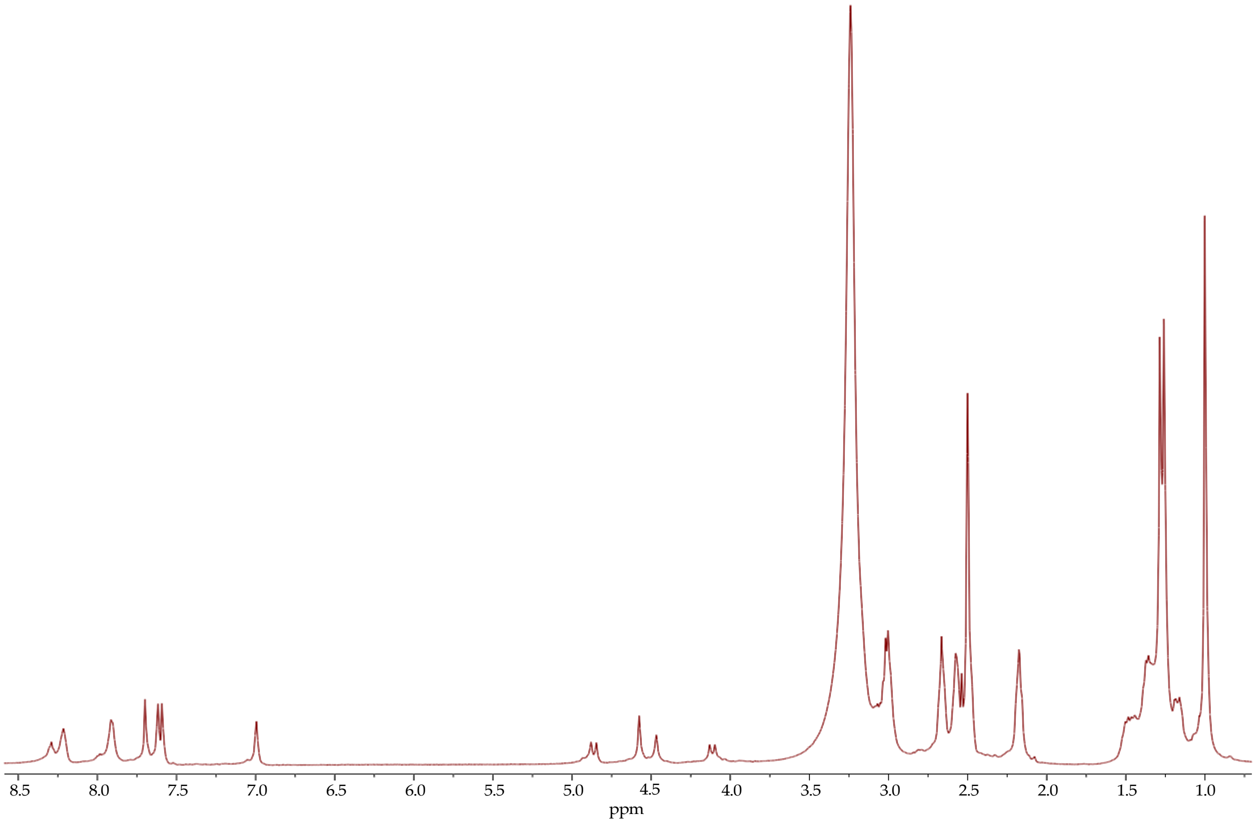

Figure S17. ^{1}H NMR spectrum of **8**, DMSO-d_6, 298 K, 400 MHz

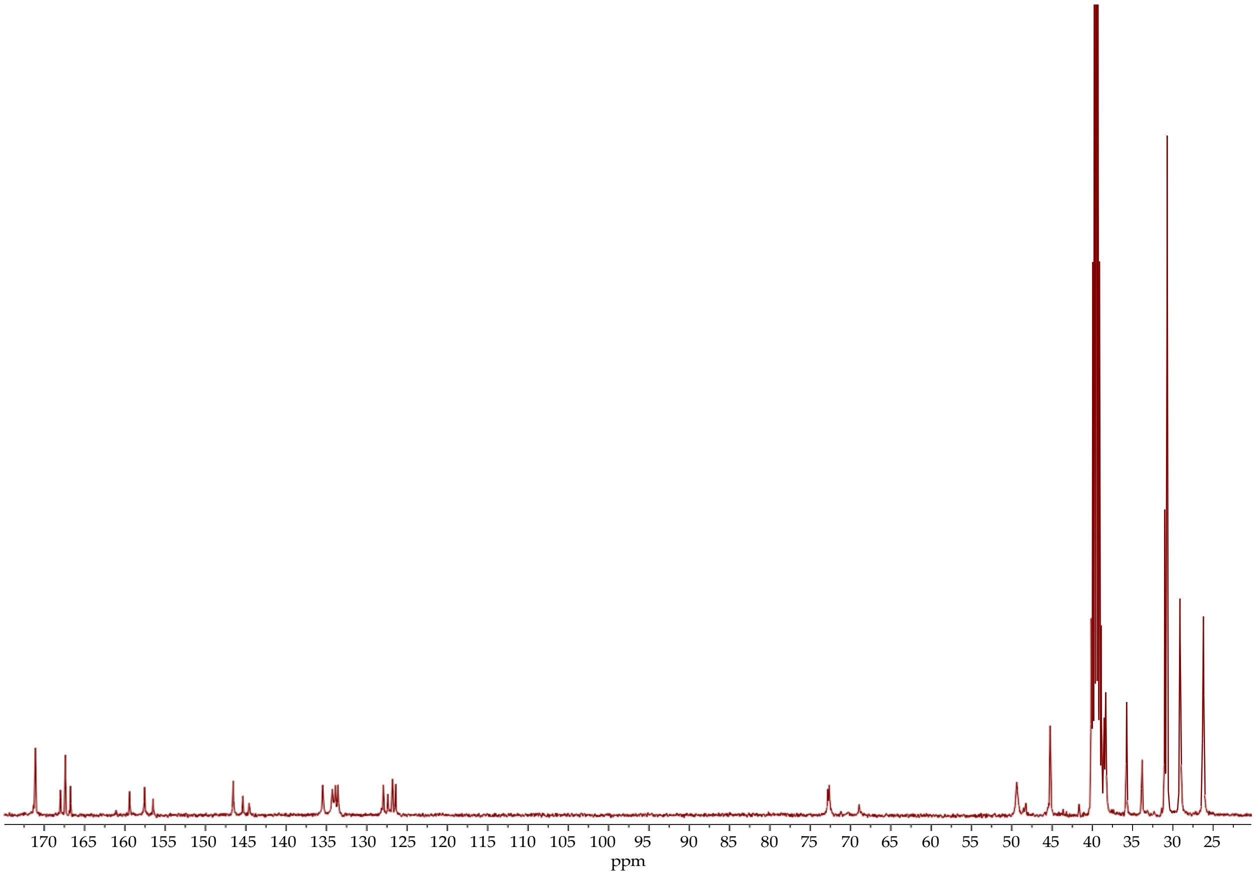

Figure S18. $^{13}C\{^{1}H\}$ NMR spectrum of **8**, DMSO-d_6, 298 K, 100 MHz

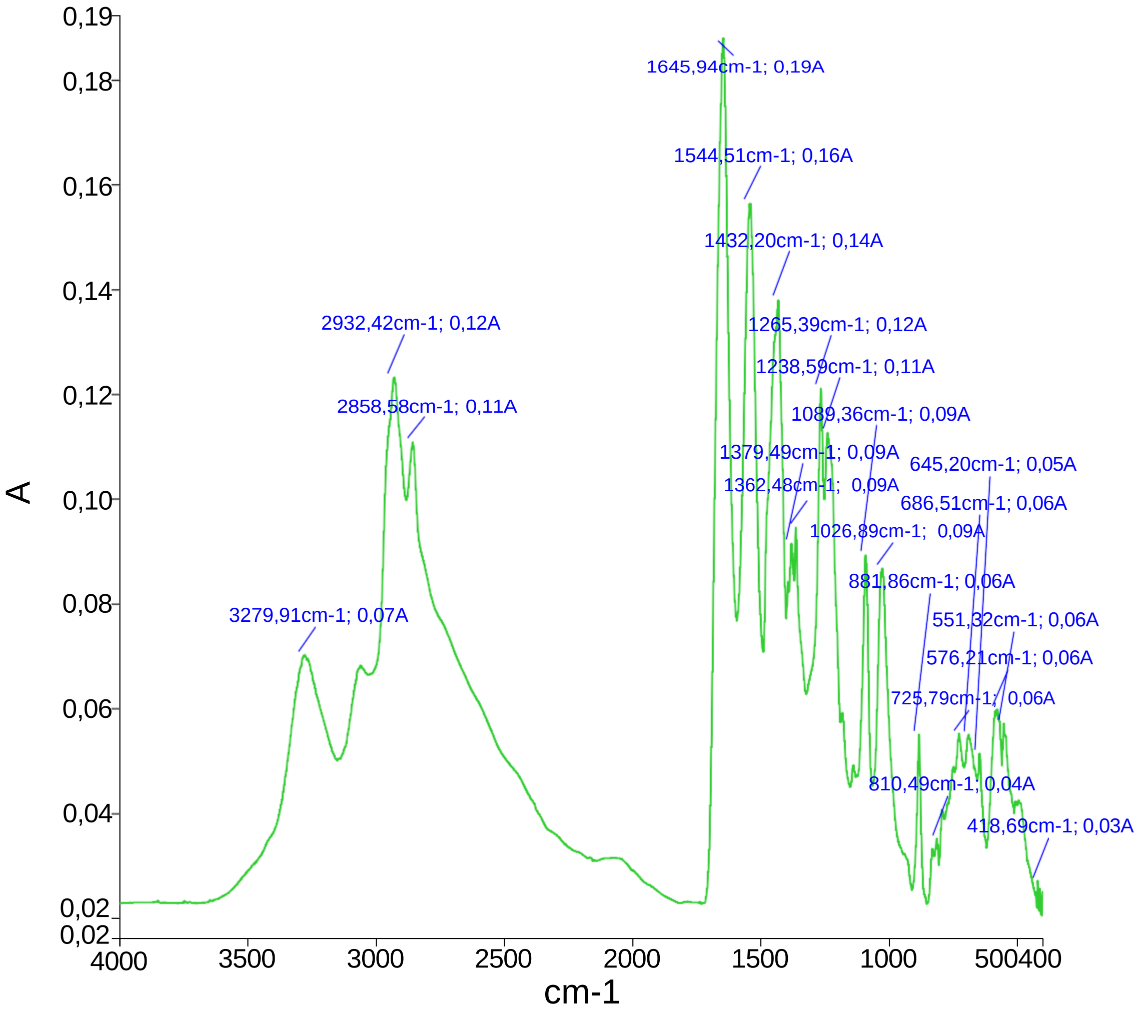

Figure S19. FTIR-ATR spectrum of **8**

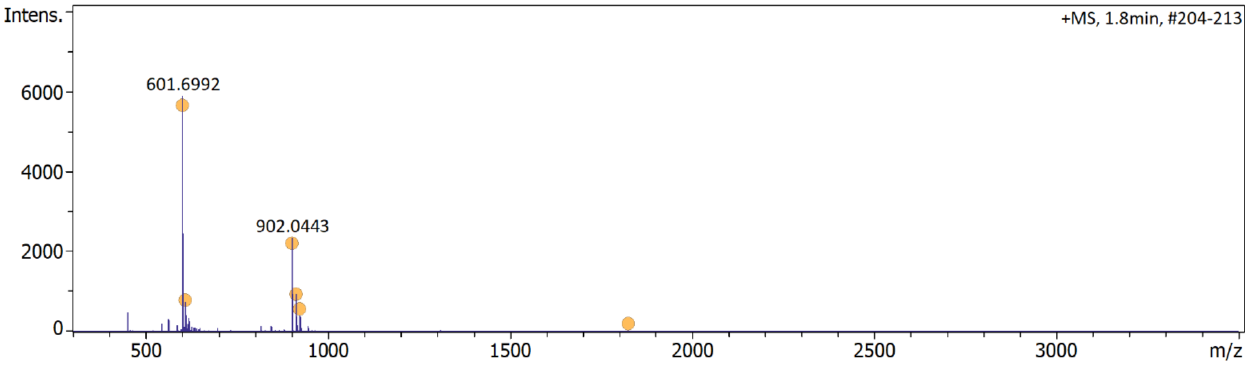

Figure S20. Mass spectrum (HR ESI) of **8**

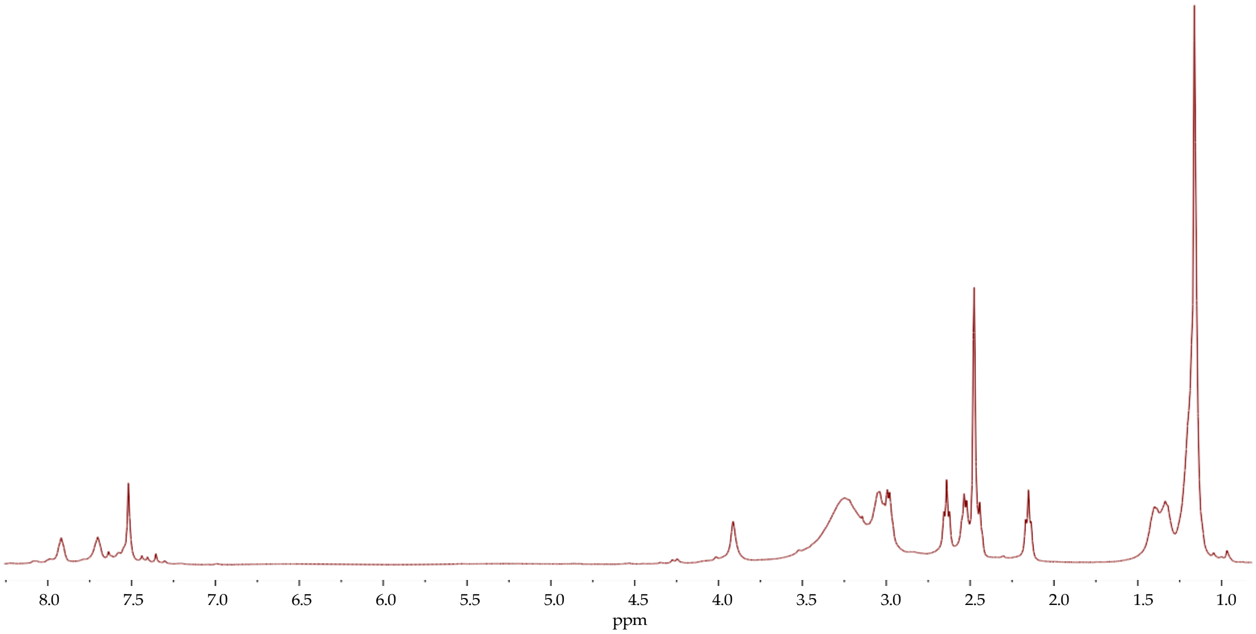

Figure S21. ^{1}H NMR spectrum of **9**, DMSO-d_6, 298 K, 400 MHz

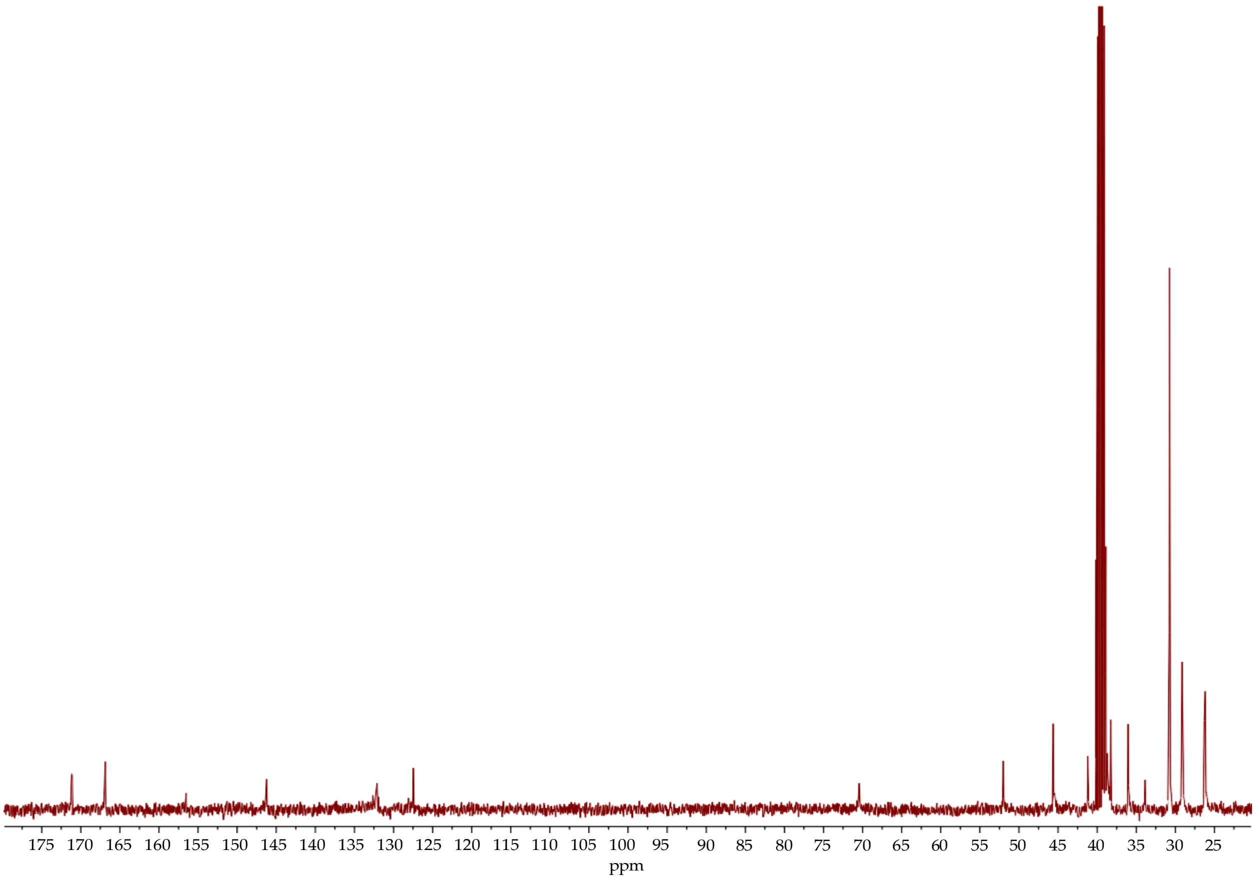

Figure S22. $^{13}C\{^{1}H\}$ NMR spectrum of **9**, DMSO-d_6, 298 K, 100 MHz

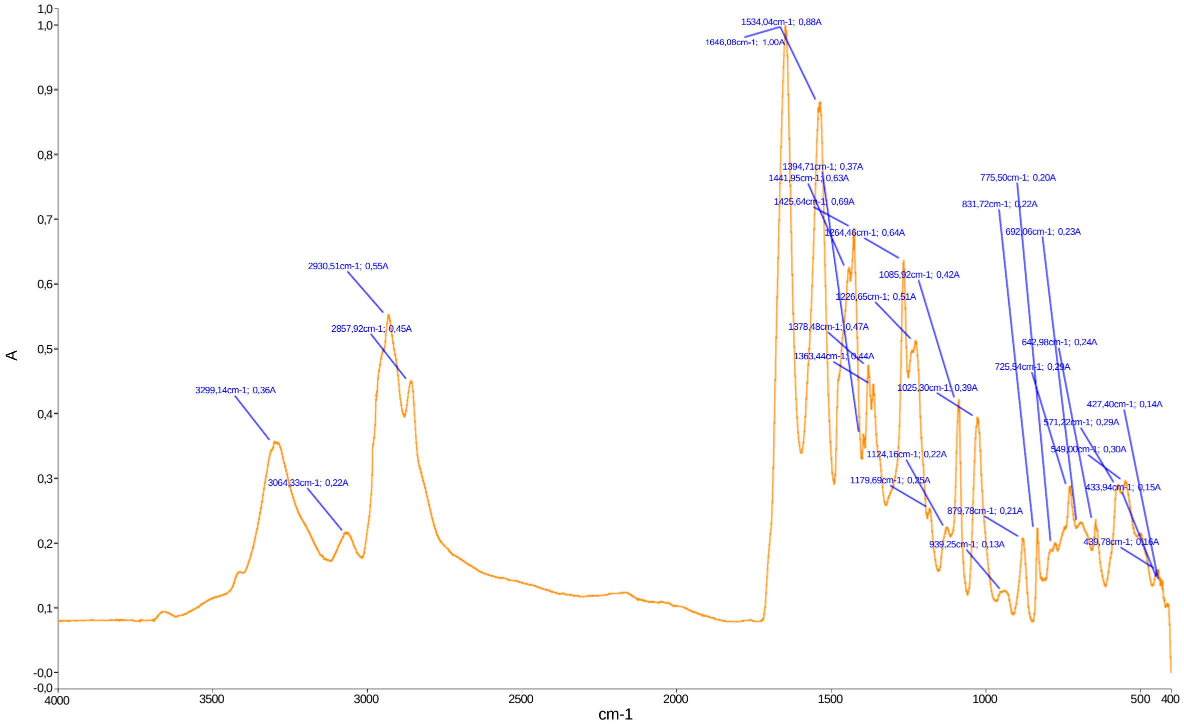

Figure S23. FTIR-ATR spectrum of **9**

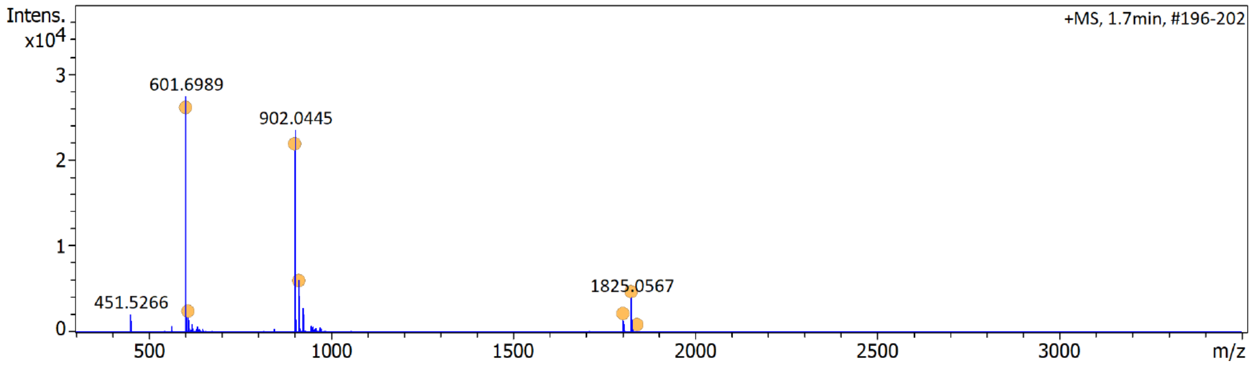

Figure S24. Mass spectrum (HR ESI) of **9**

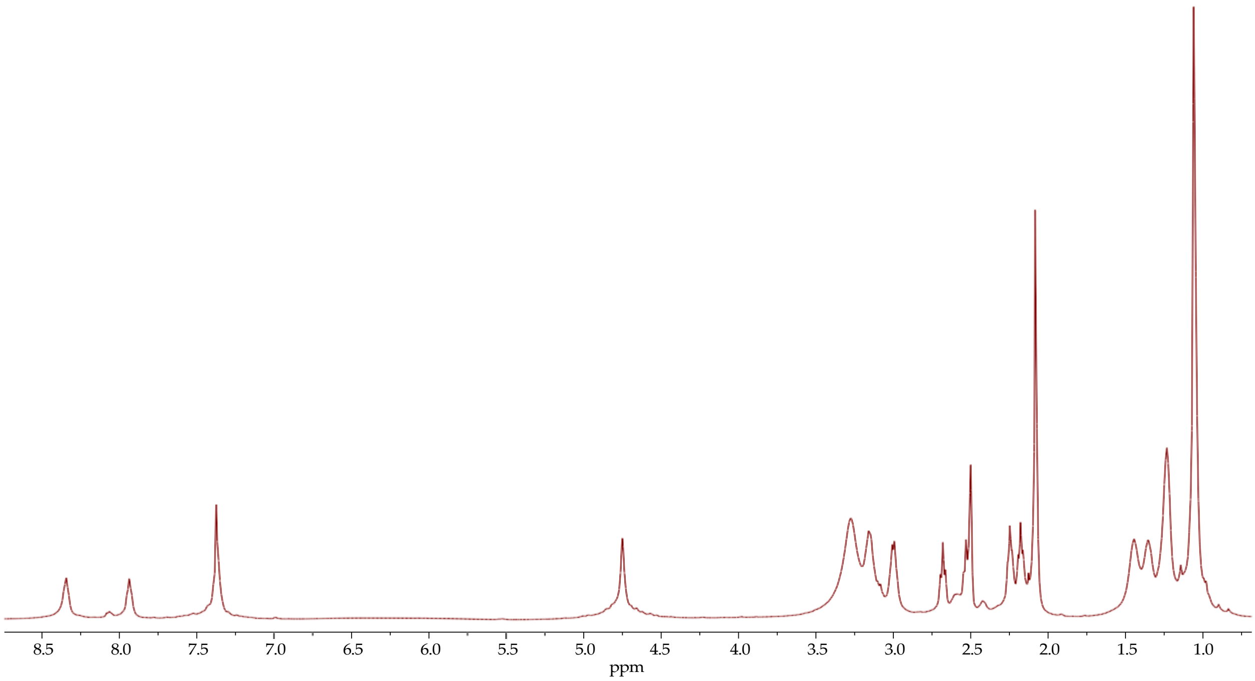

Figure S25. ^{1}H NMR spectrum of **10**, DMSO-d_6, 298 K, 400 MHz

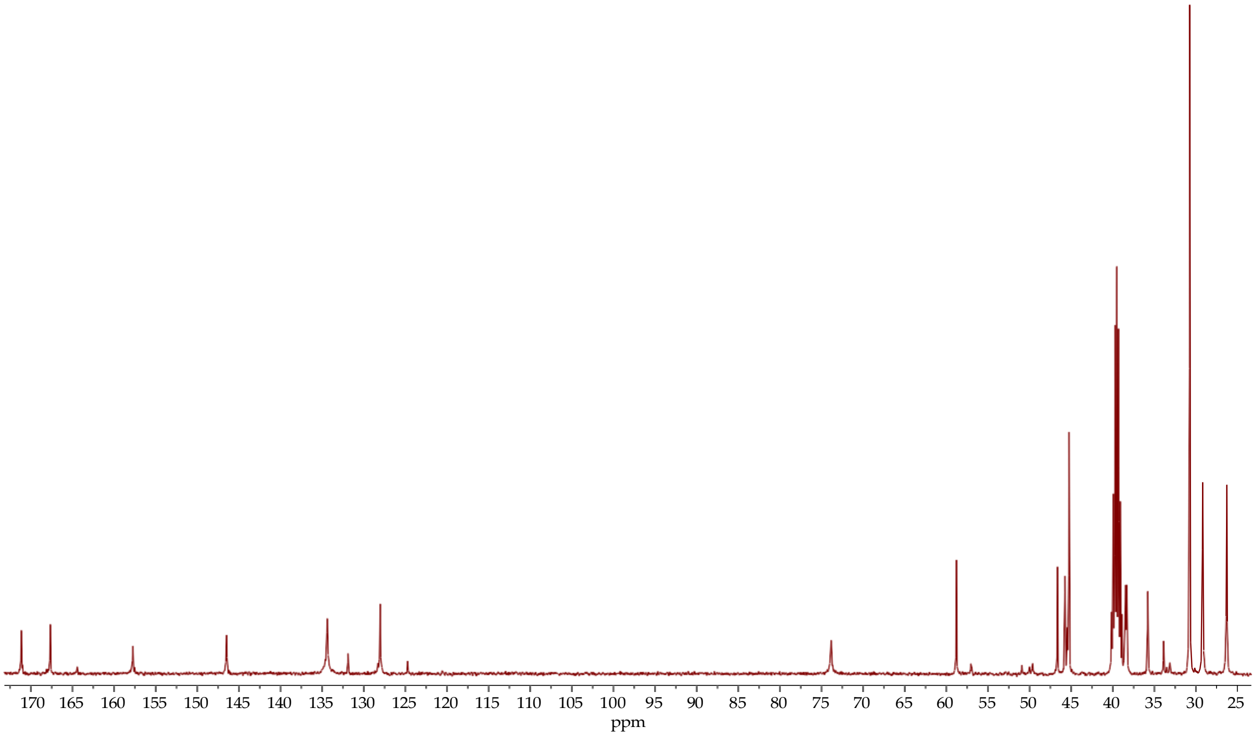

Figure S26. $^{13}C\{^{1}H\}$ NMR spectrum of **10**, DMSO-d_6, 298 K, 100 MHz

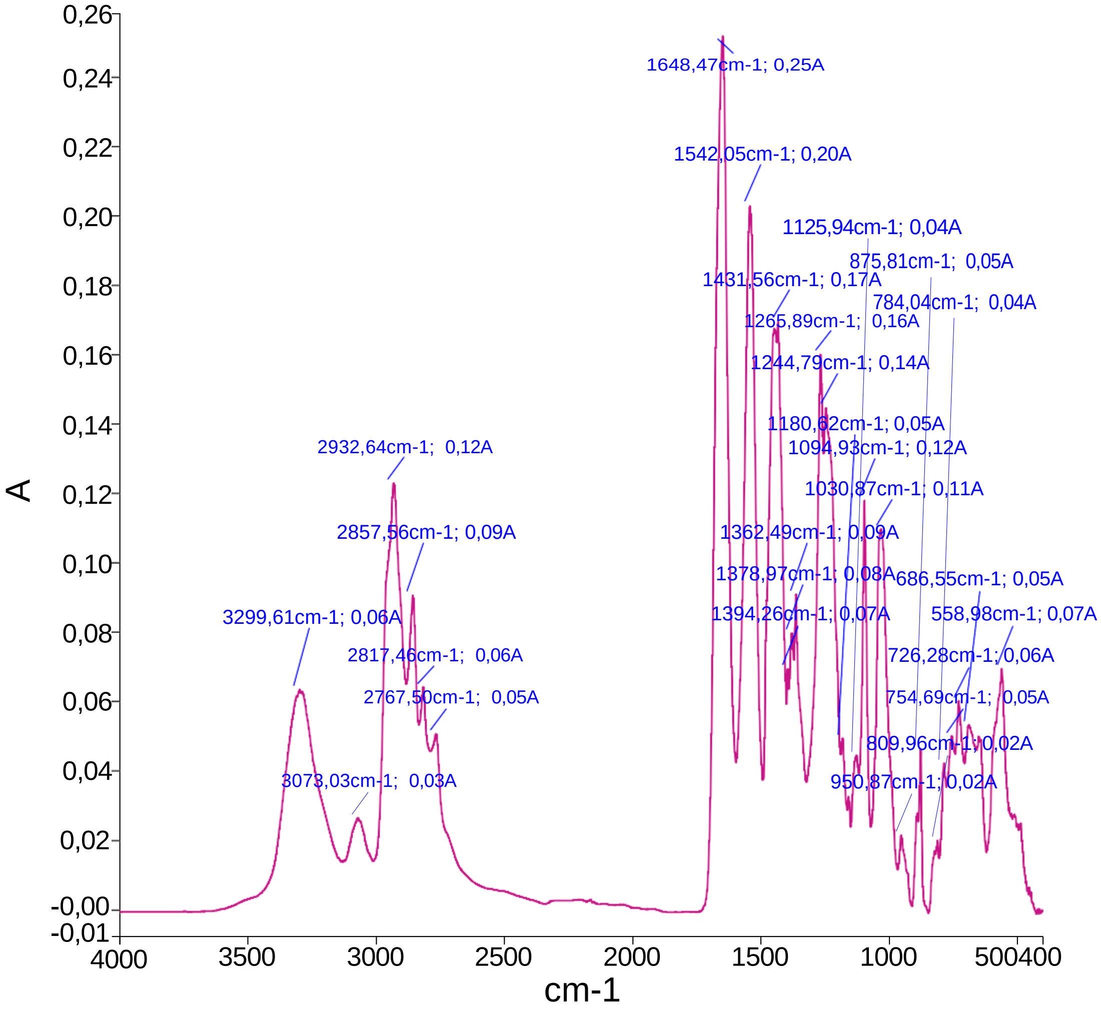

Figure S27. FTIR-ATR spectrum of **10**

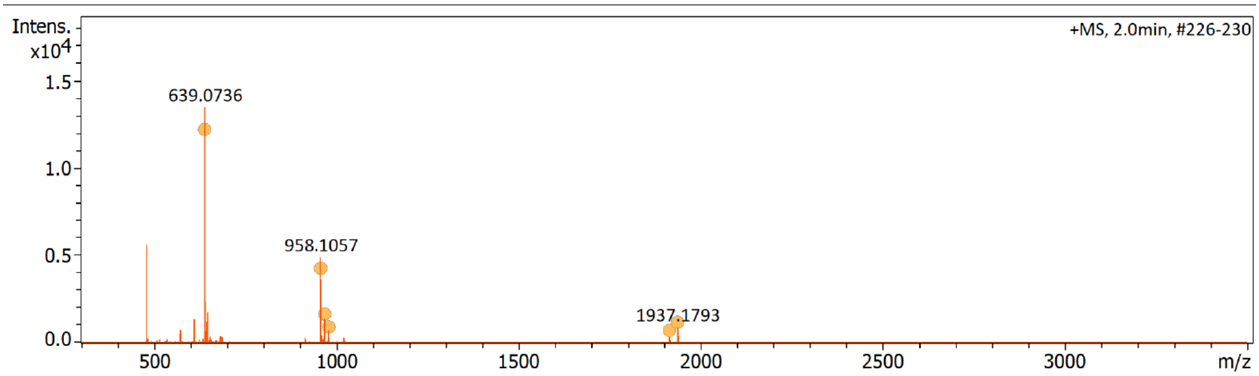

Figure S28. Mass spectrum (HR ESI) of **10**

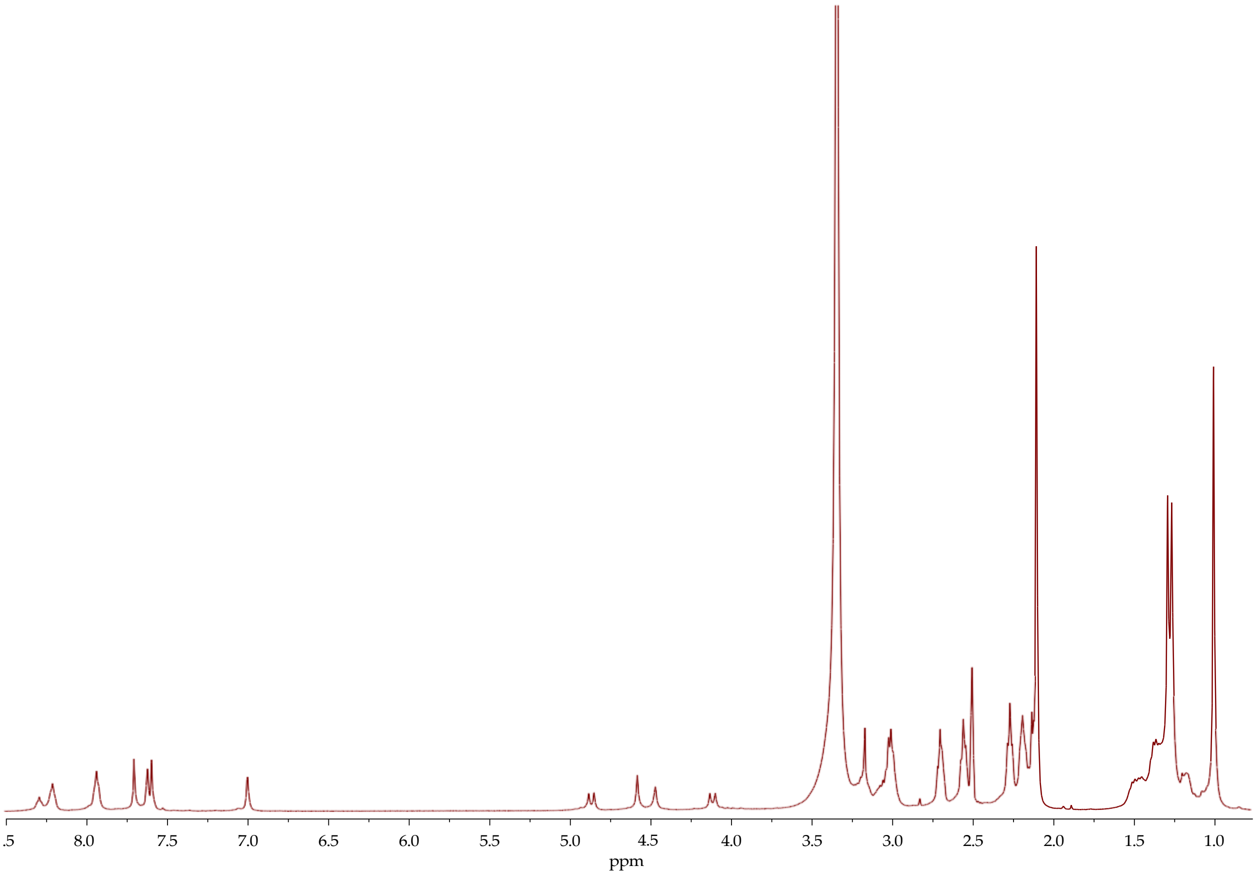

Figure S29. ^{1}H NMR spectrum of **11**, DMSO-d_6, 298 K, 400 MHz

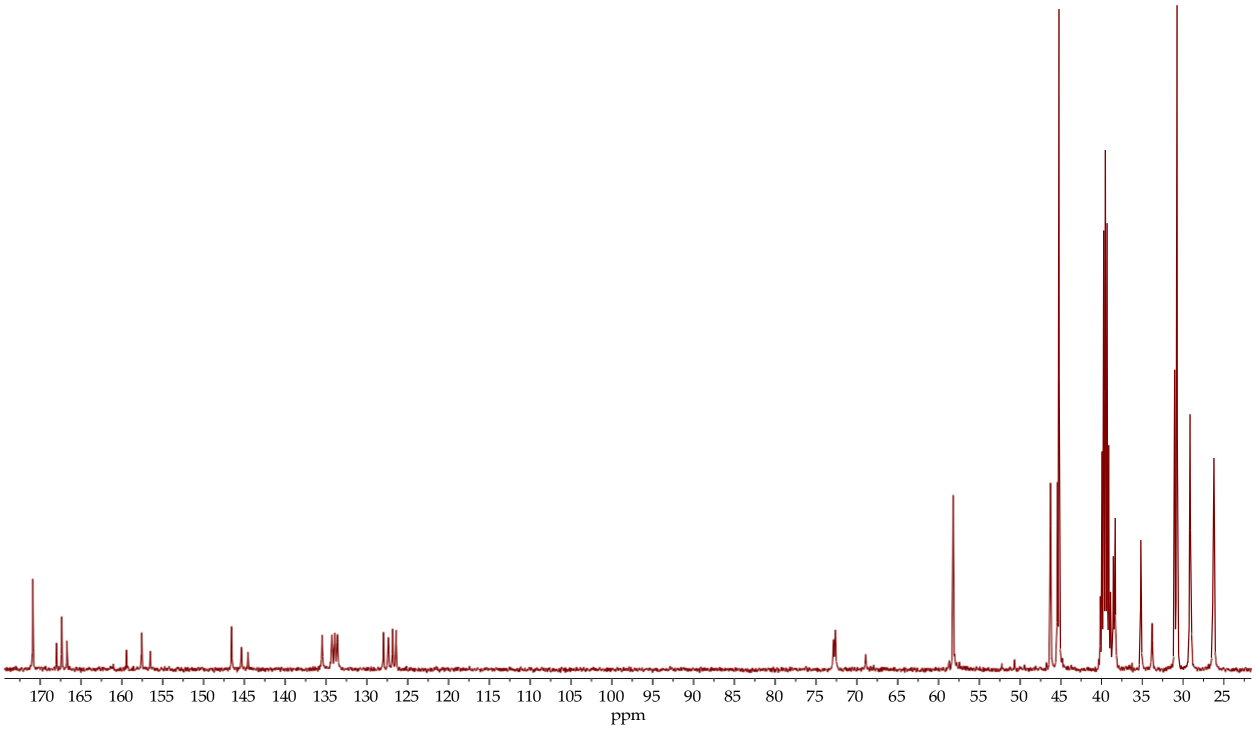

Figure S30. $^{13}C\{^{1}H\}$ NMR spectrum of **11**, DMSO-d_6, 298 K, 100 MHz

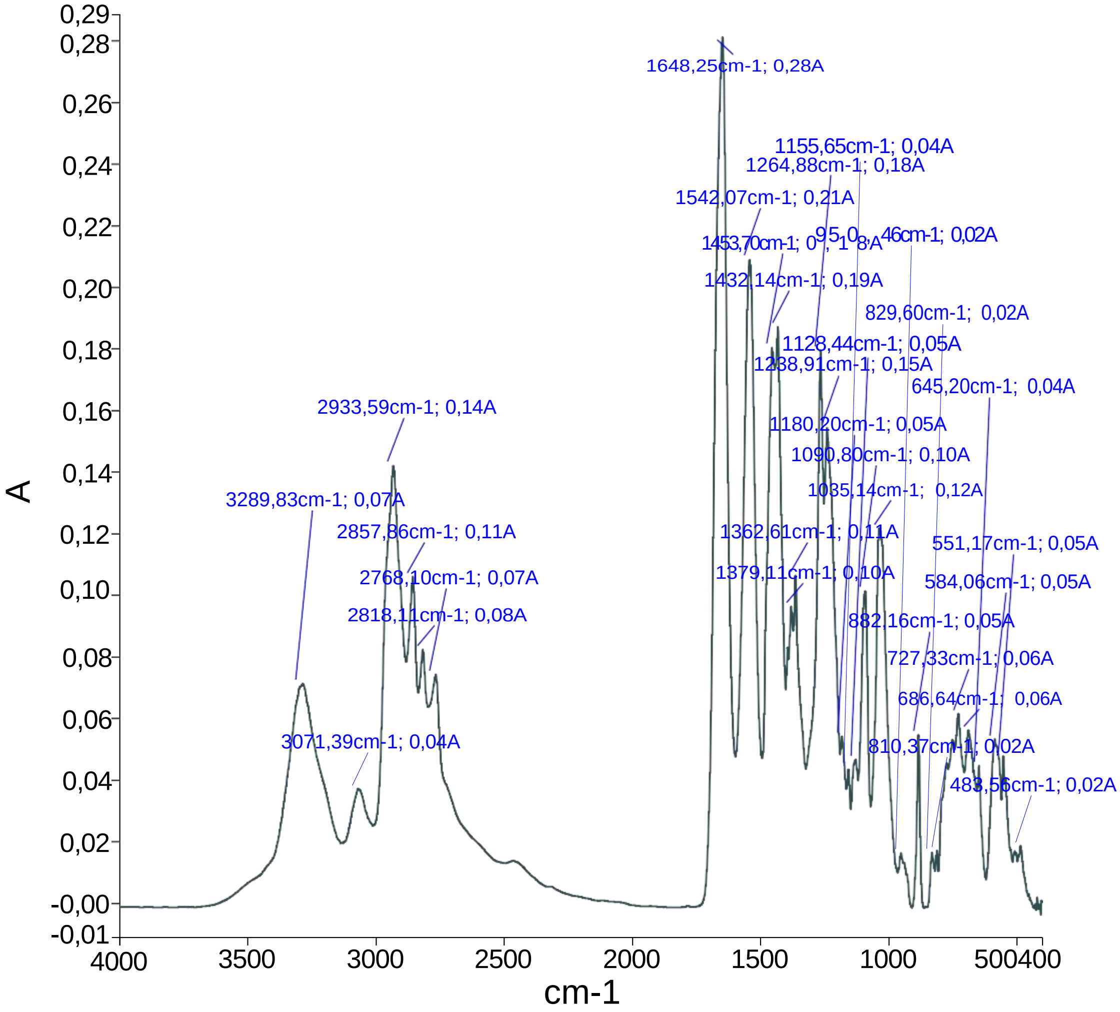

Figure S31. FTIR-ATR spectrum of **11**

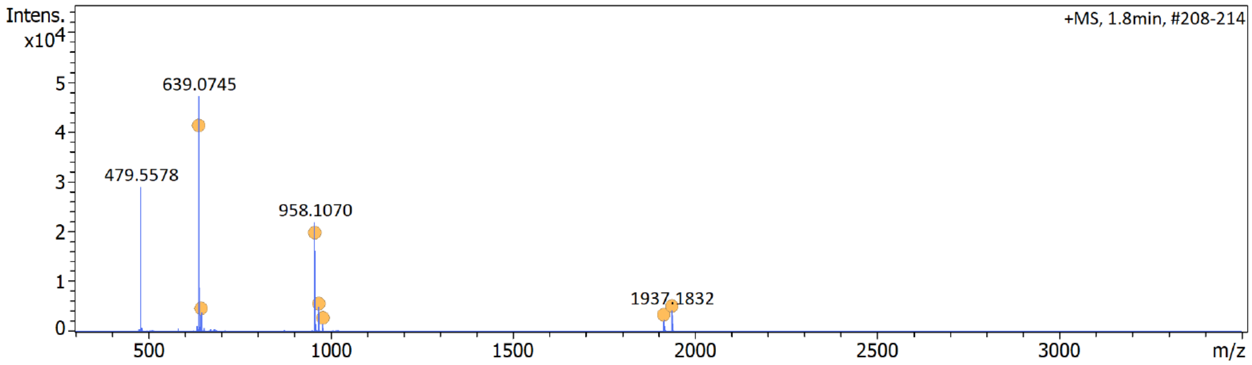

Figure S32. Mass spectrum (HR ESI) of **11**

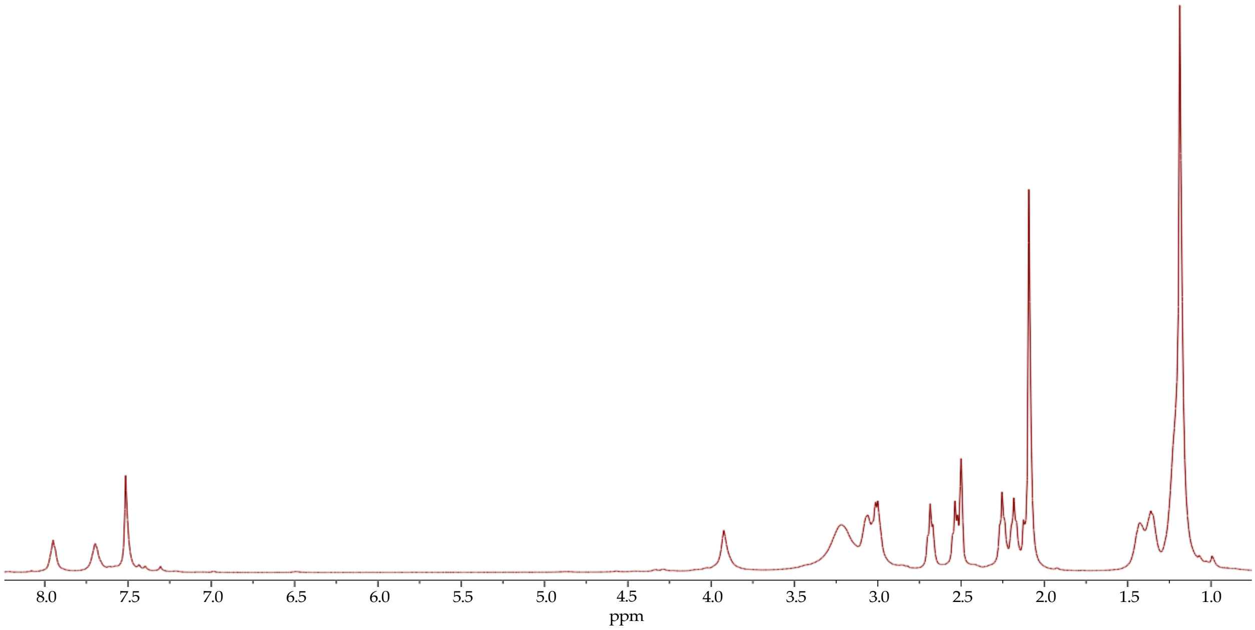

Figure S33. ^{1}H NMR spectrum of **12**, DMSO-d_6, 298 K, 400 MHz

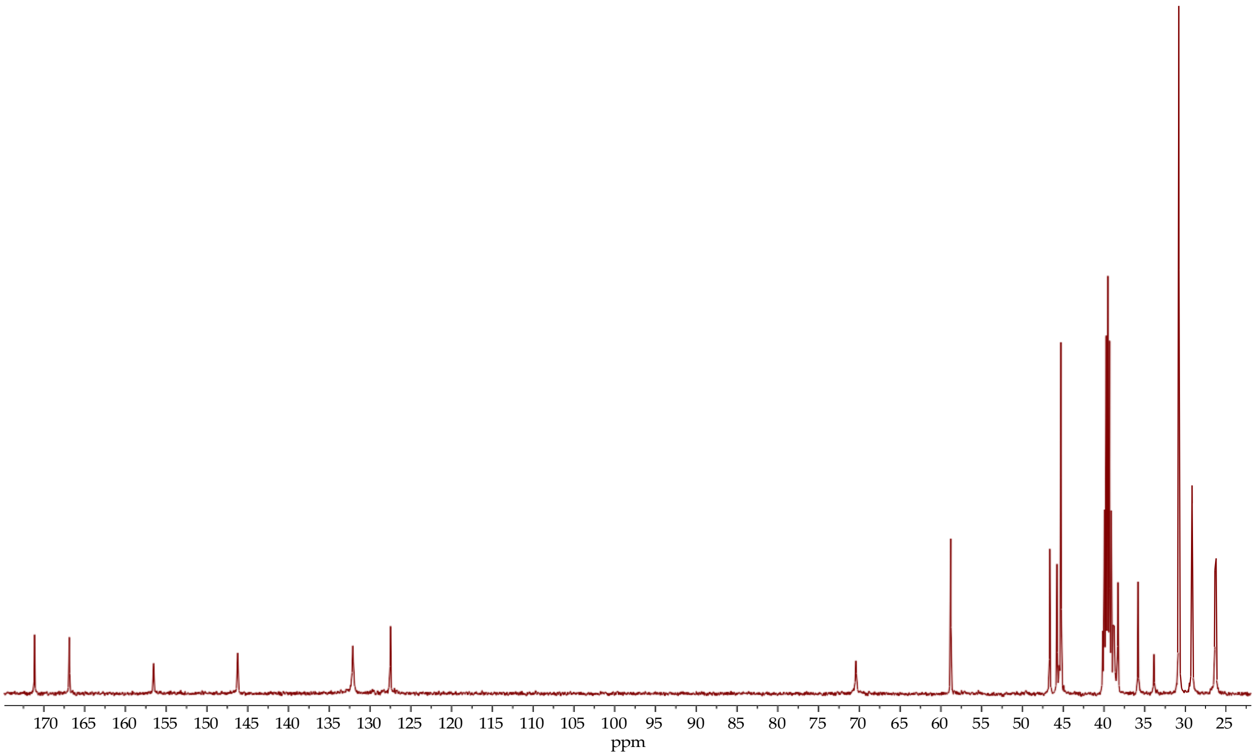

Figure S34. $^{13}C\{^{1}H\}$ NMR spectrum of **12**, DMSO-d_6, 298 K, 100 MHz

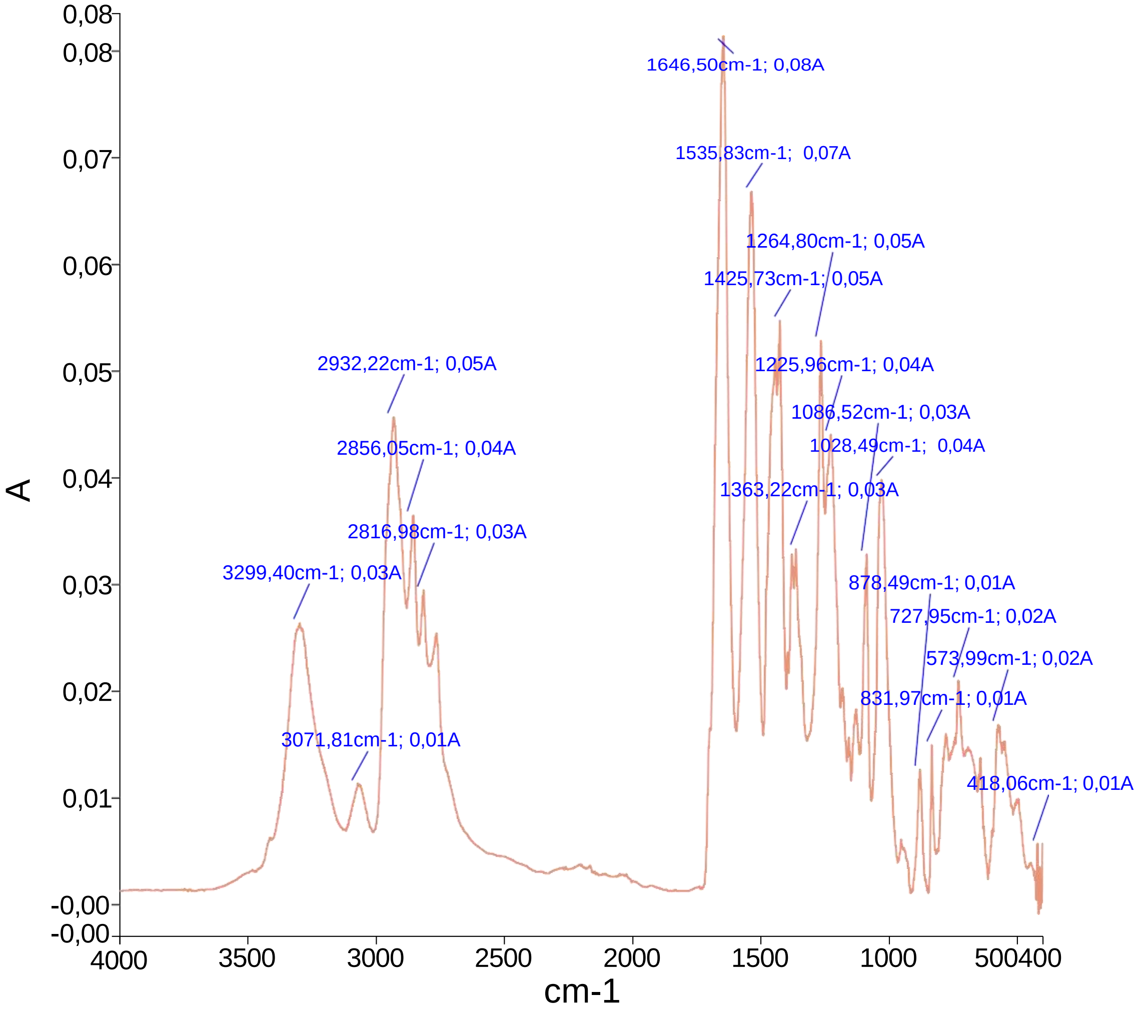

Figure S35. FTIR-ATR spectrum of **12**

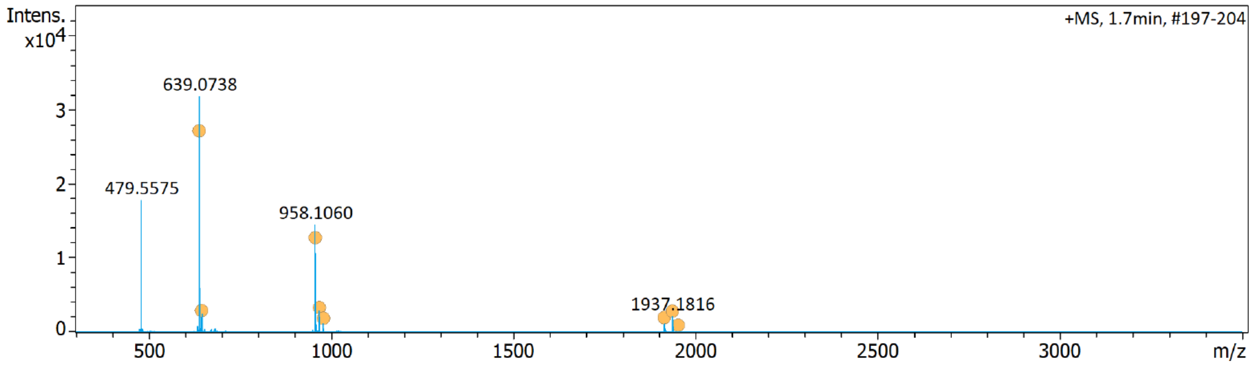

Figure S36. Mass spectrum (HR ESI) of **12**

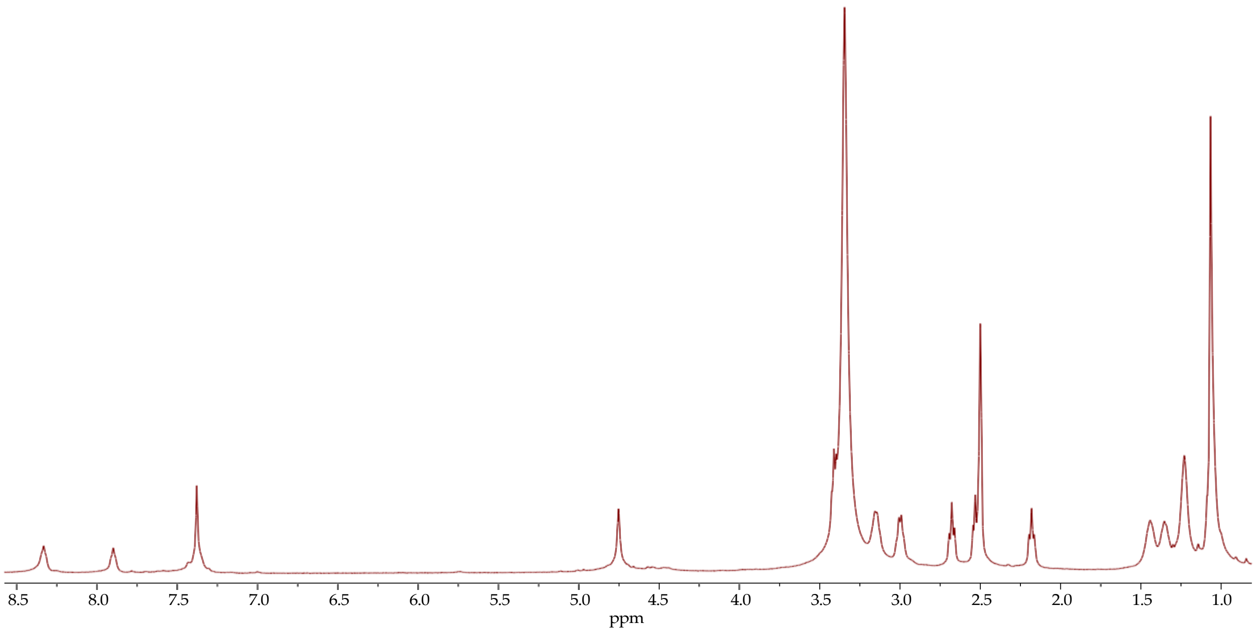

Figure S37. ^{1}H NMR spectrum of **13**, DMSO-d_6, 298 K, 400 MHz

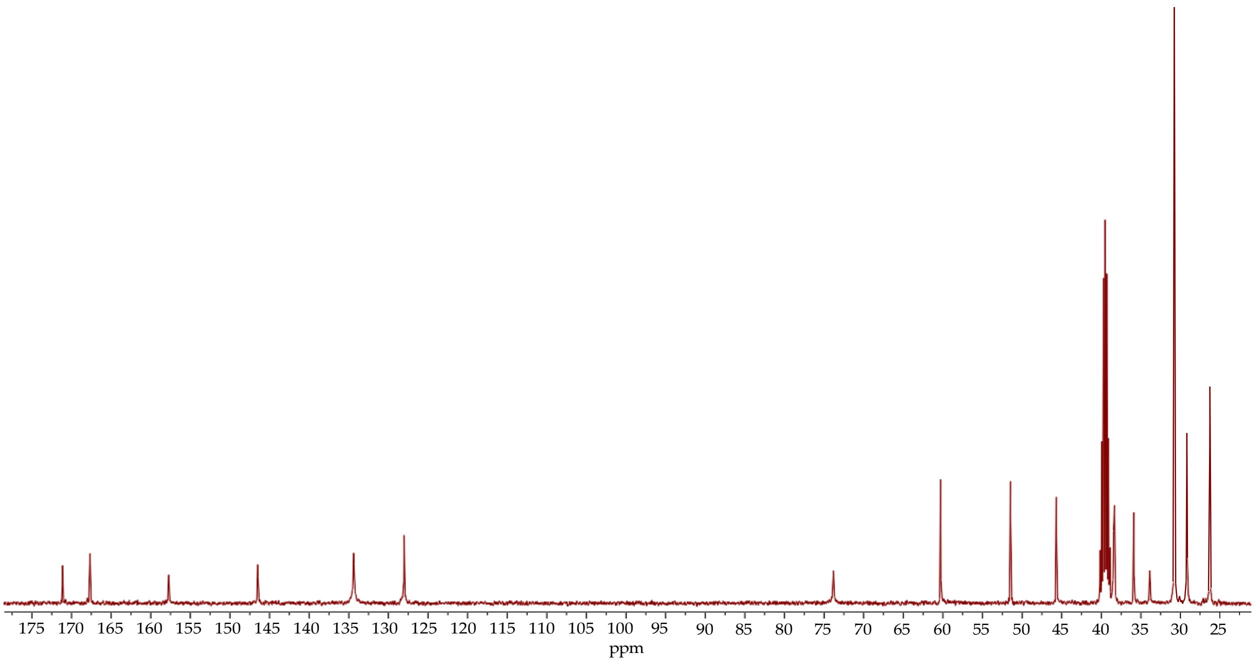

Figure S38. ^{13}C{^{1}H} NMR spectrum of **13**, DMSO-d_6, 298 K, 100 MHz

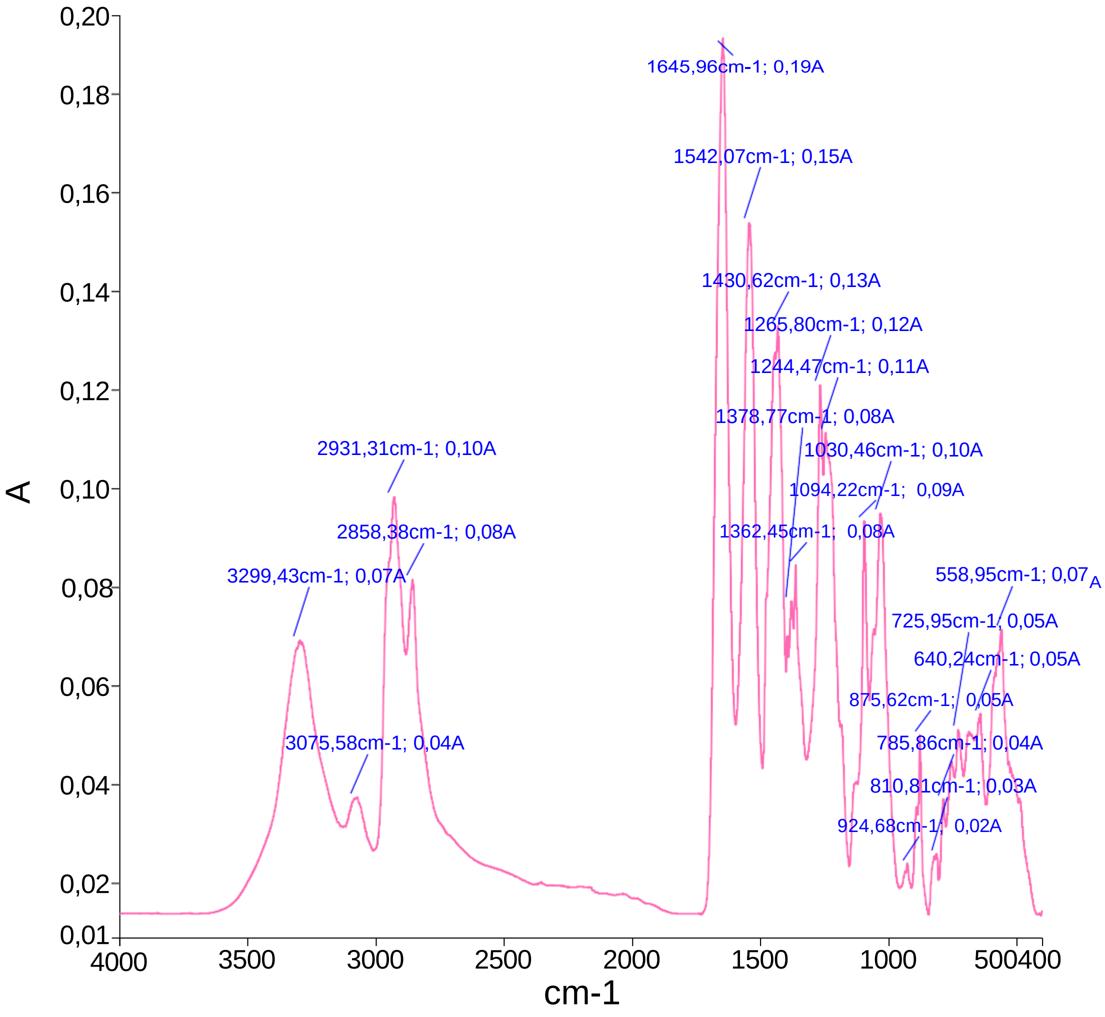

Figure S39. FTIR-ATR spectrum of **13**

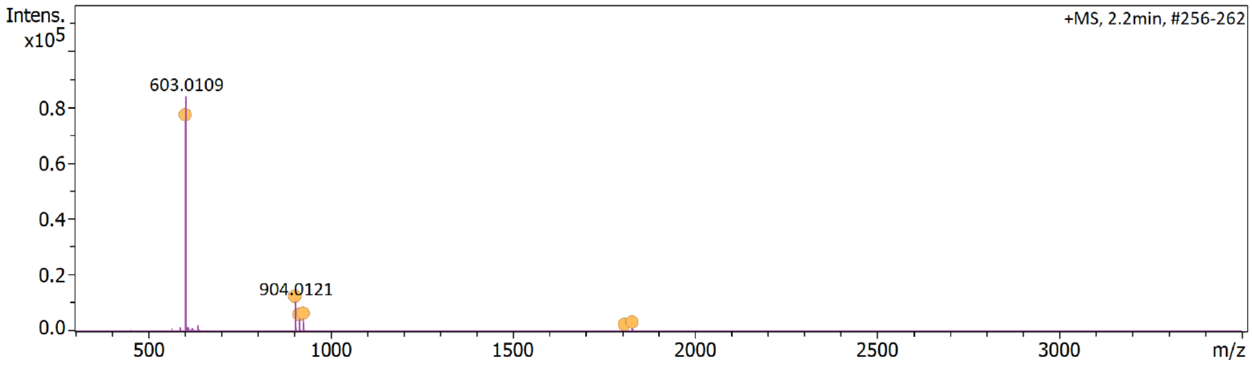

Figure S40. Mass spectrum (HR ESI) of **13**

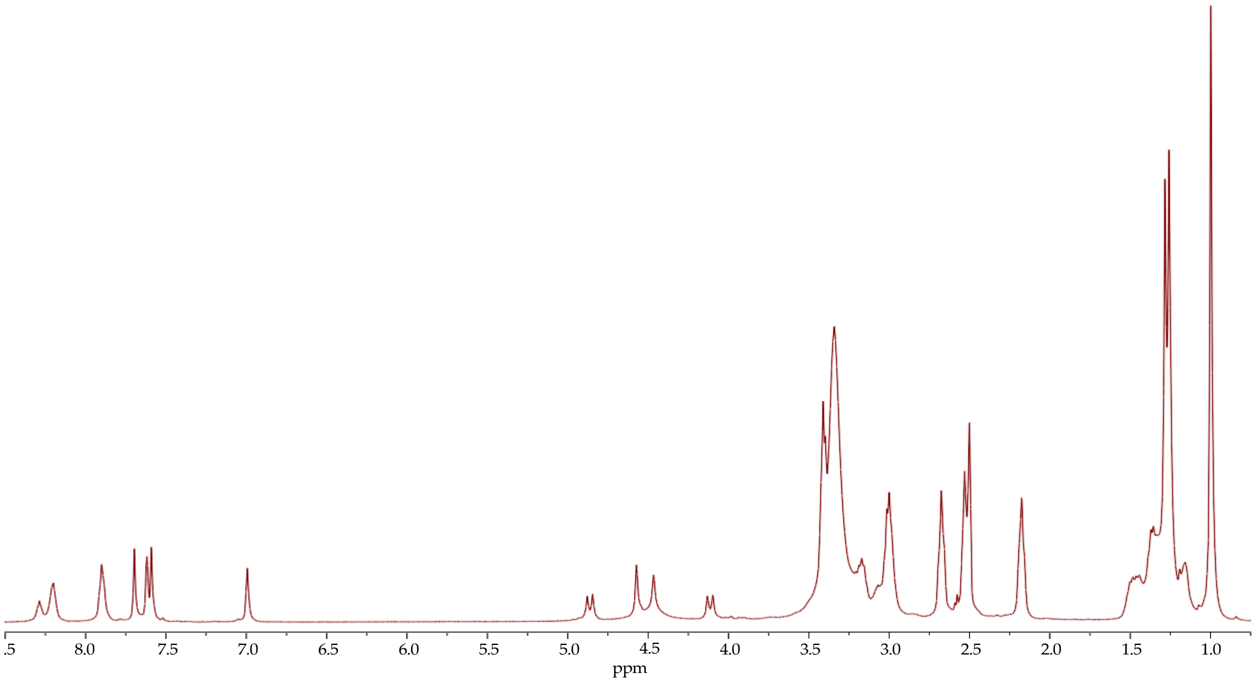

Figure S41. ^{1}H NMR spectrum of **14**, DMSO-*d*6, 298 K, 400 MHz

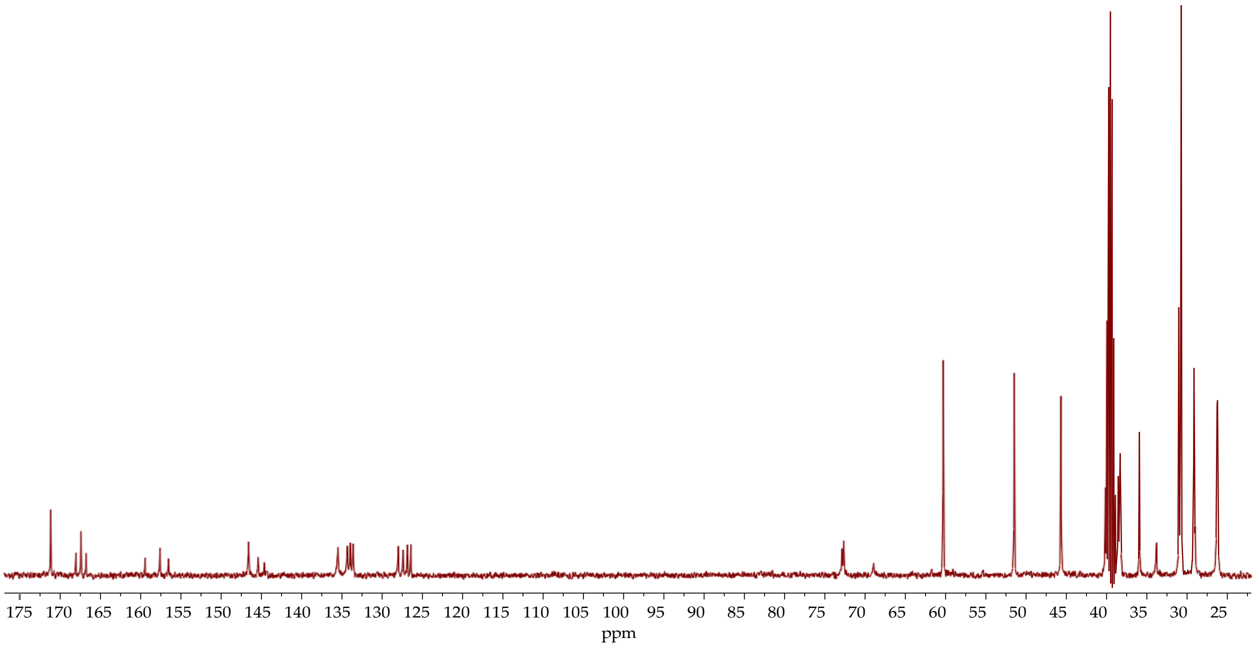

Figure S42. ^{13}C{^{1}H} NMR spectrum of **14**, DMSO-*d*6, 298 K, 100 MHz

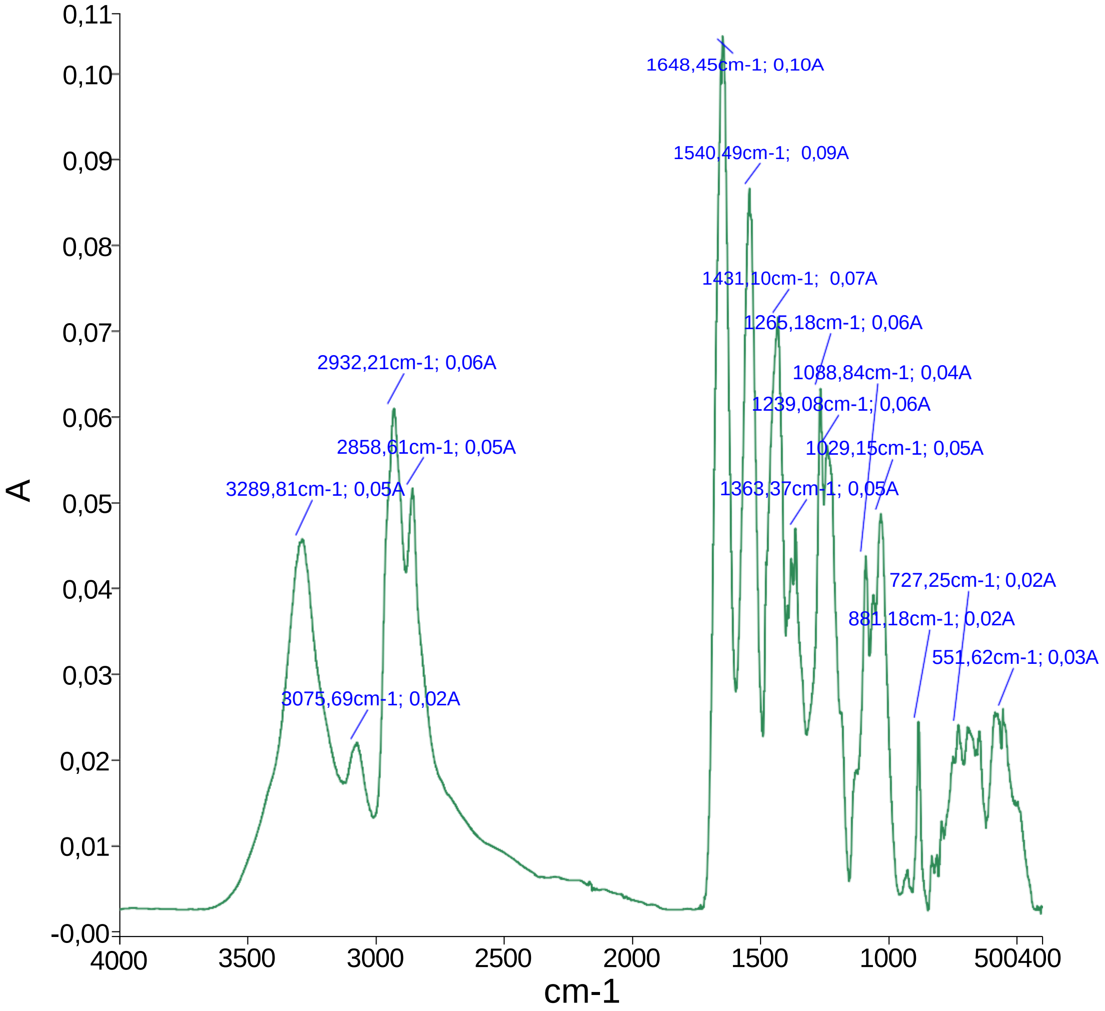

Figure S43. FTIR-ATR spectrum of **14**

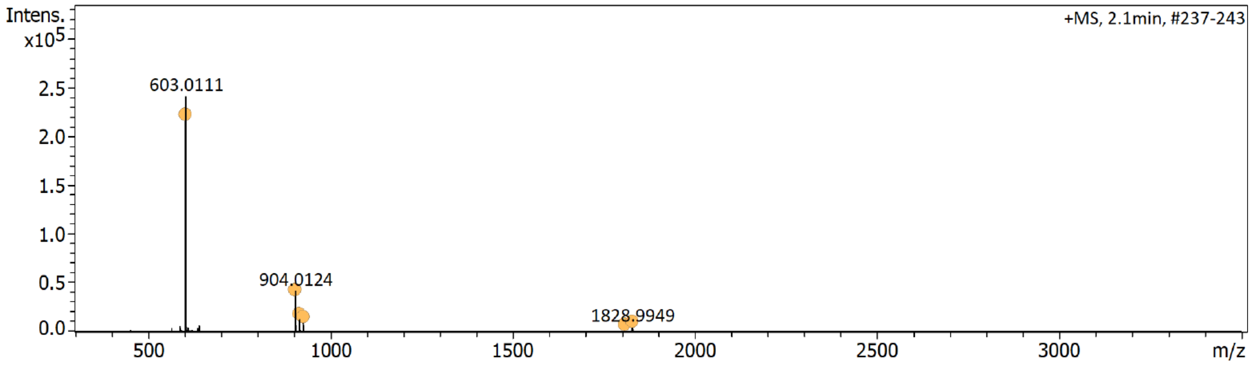

Figure S44. Mass spectrum (HR ESI) of **14**

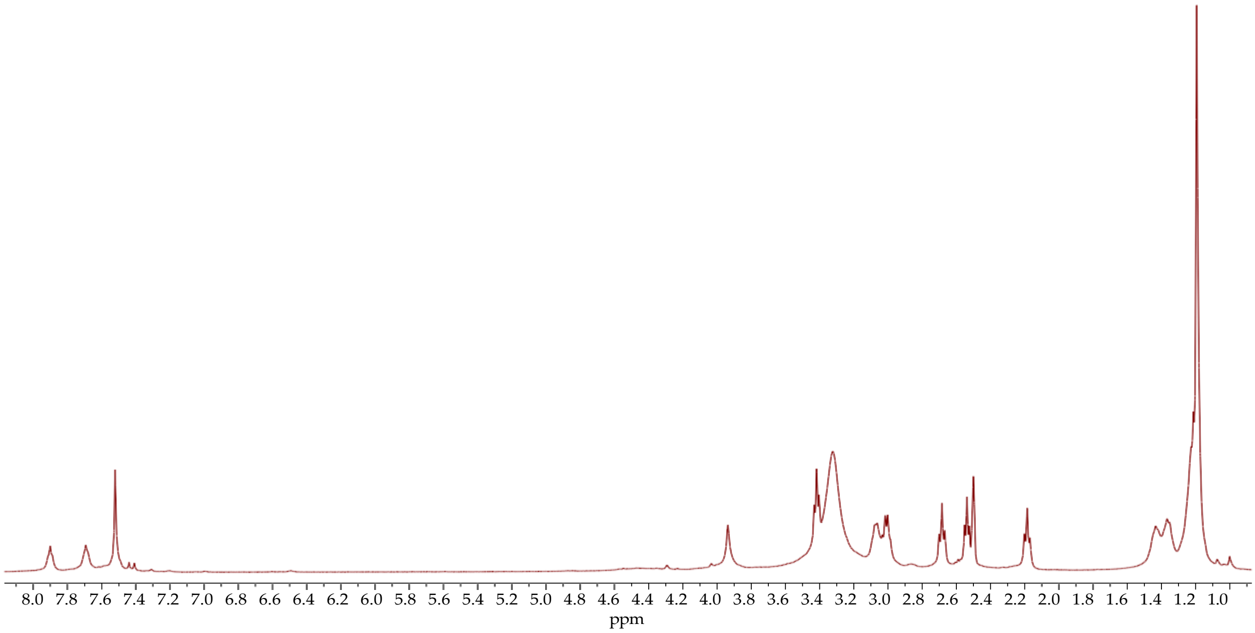

Figure S45. ^{1}H NMR spectrum of **15**, DMSO-d_6, 298 K, 400 MHz

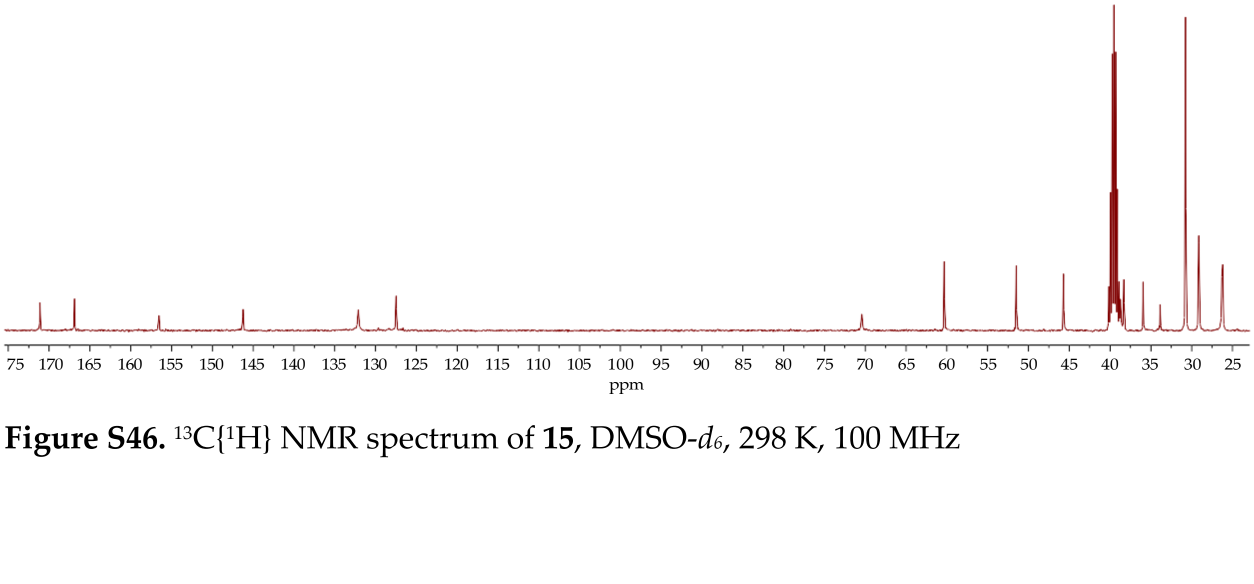

Figure S46. $^{13}C\{^{1}H\}$ NMR spectrum of **15**, DMSO-d_6, 298 K, 100 MHz

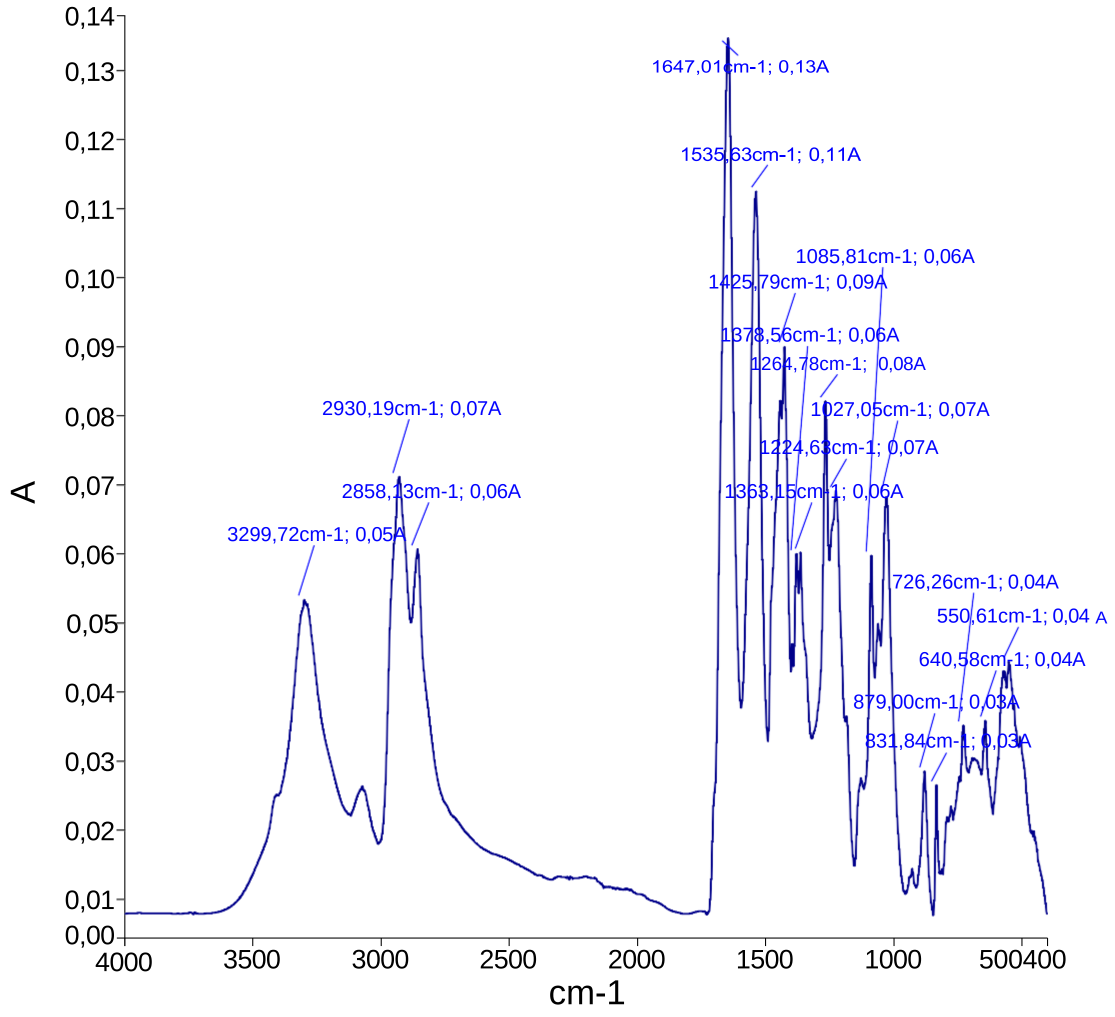

Figure S47. FTIR-ATR spectrum of **15**

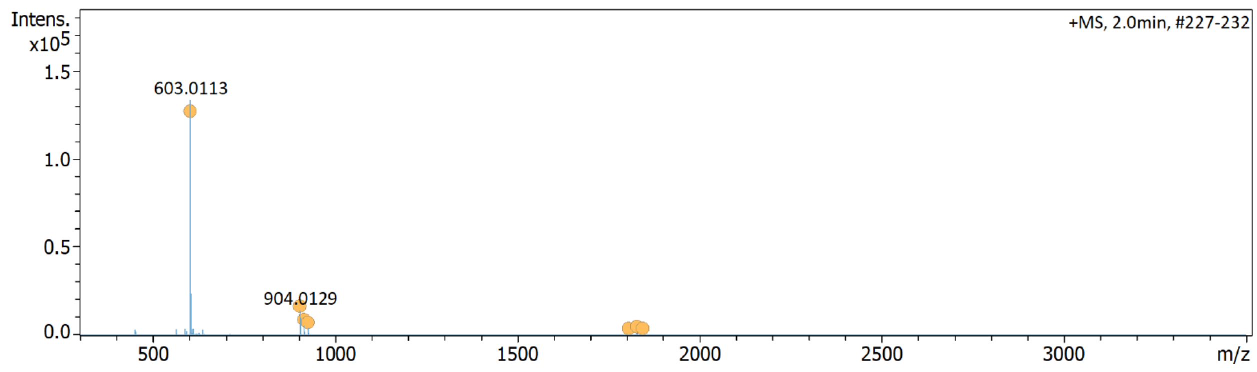

Figure S48. Mass spectrum (HR ESI) of **15**

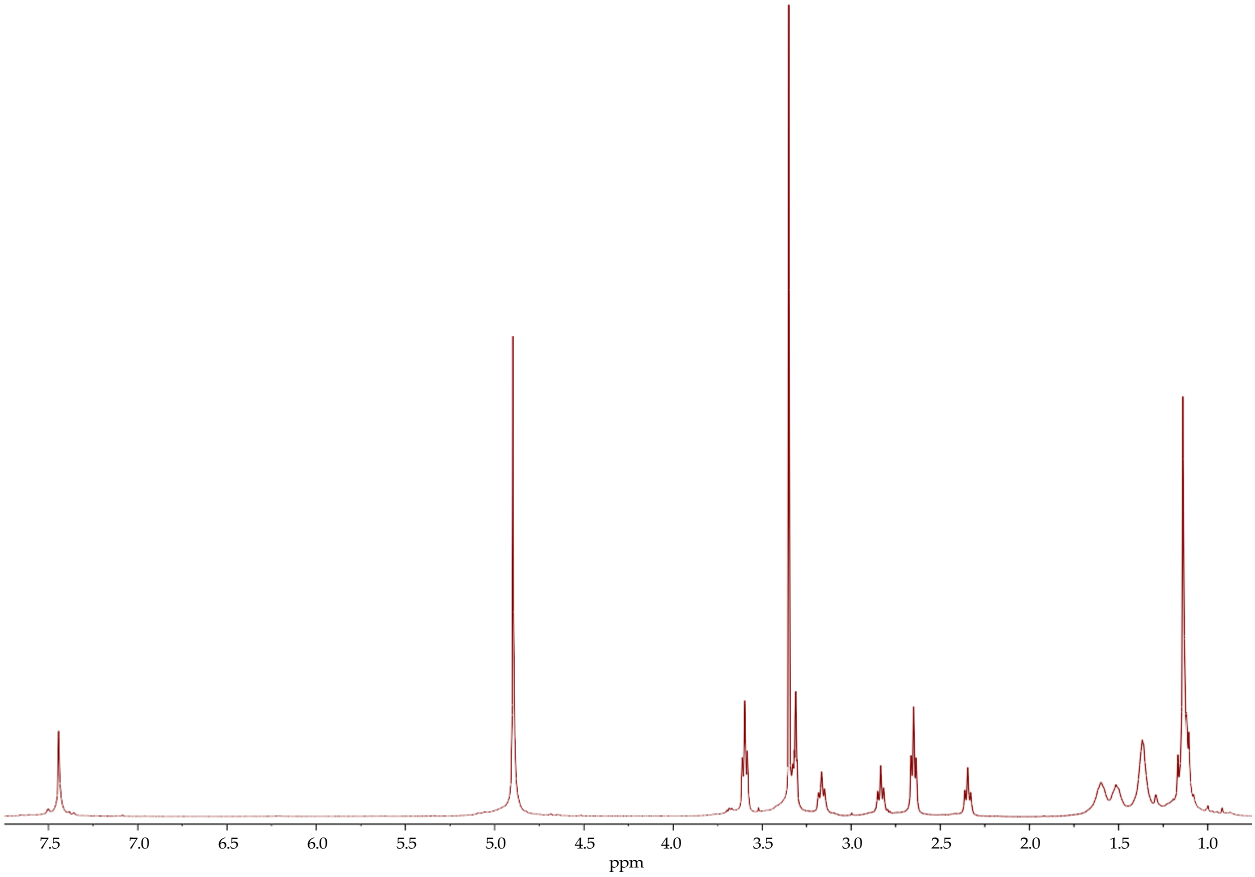

Figure S49. ^{1}H NMR spectrum of **16**, CD_3OD, 298 K, 400 MHz

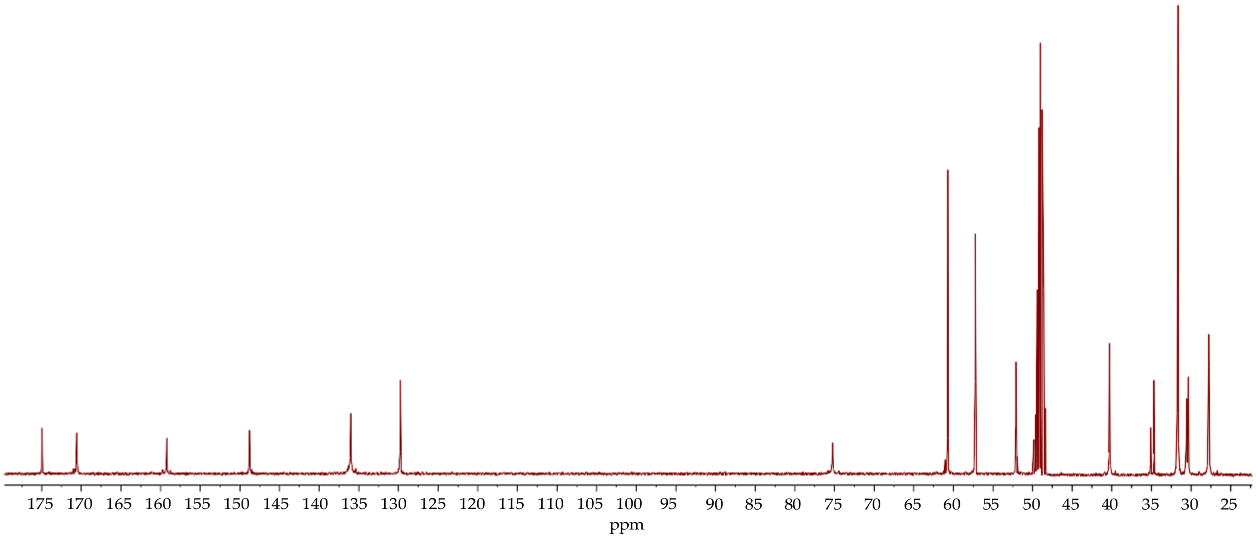

Figure S50. $^{13}C\{^1H\}$ NMR spectrum of **16**, CD_3OD, 298 K, 100 MHz

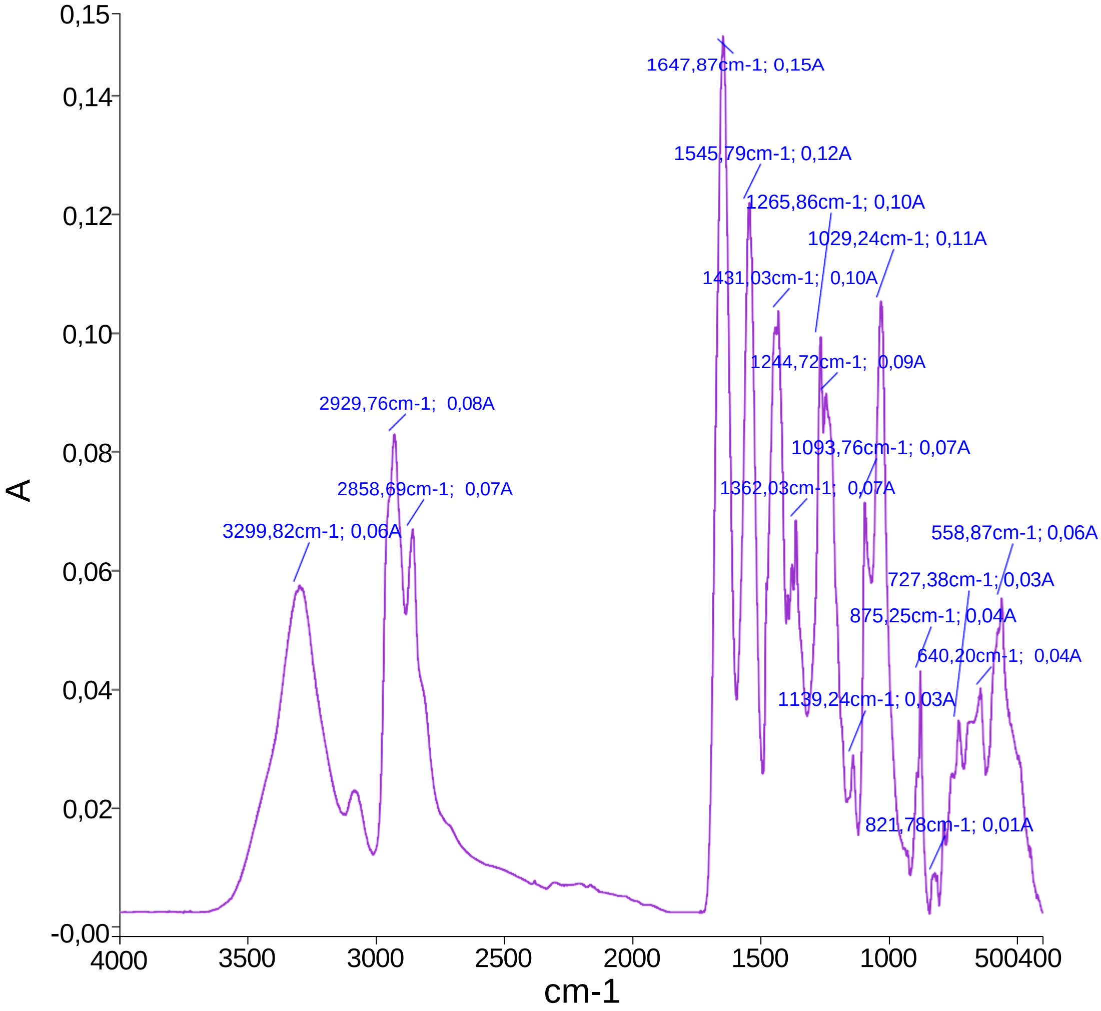

Figure S51. FTIR-ATR spectrum of **16**

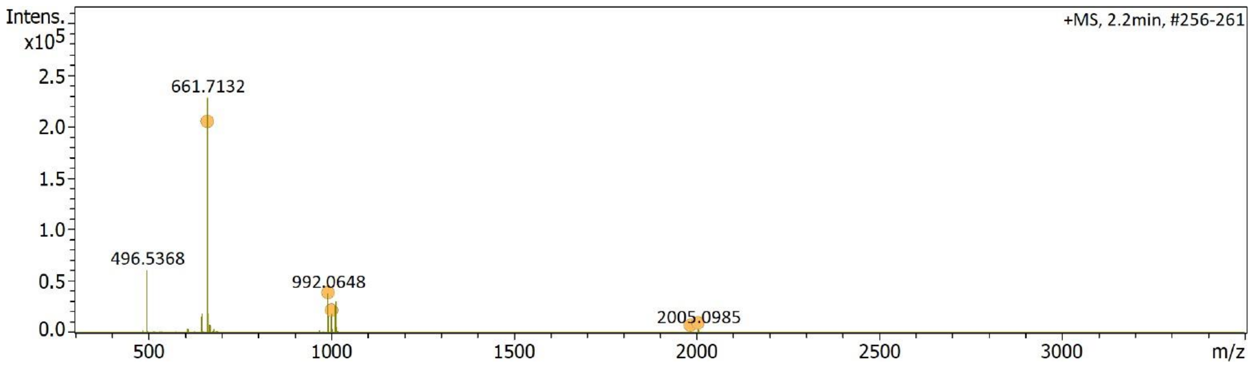

Figure S52. Mass spectrum (HR ESI) of **16**

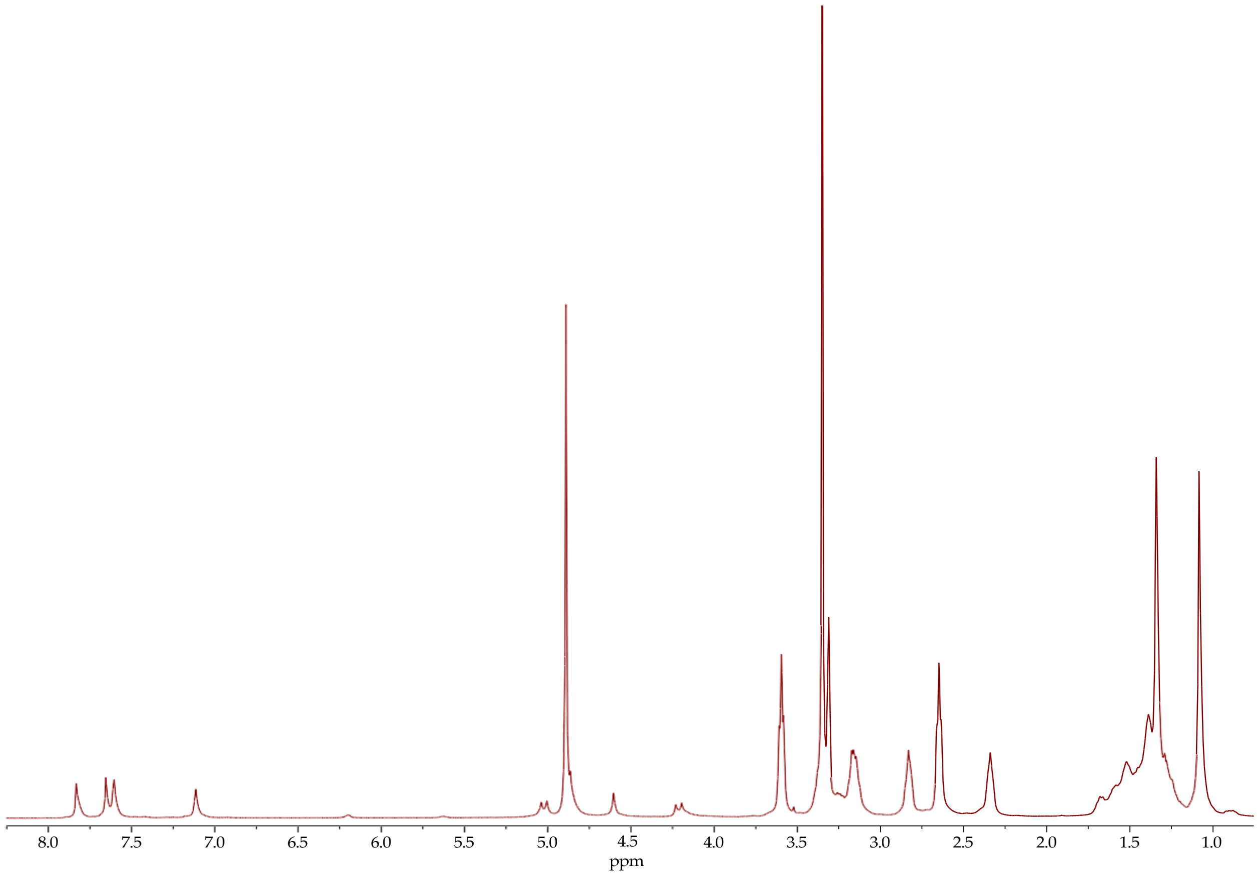

Figure S53. ^{1}H NMR spectrum of **17**, CD_3OD, 298 K, 400 MHz

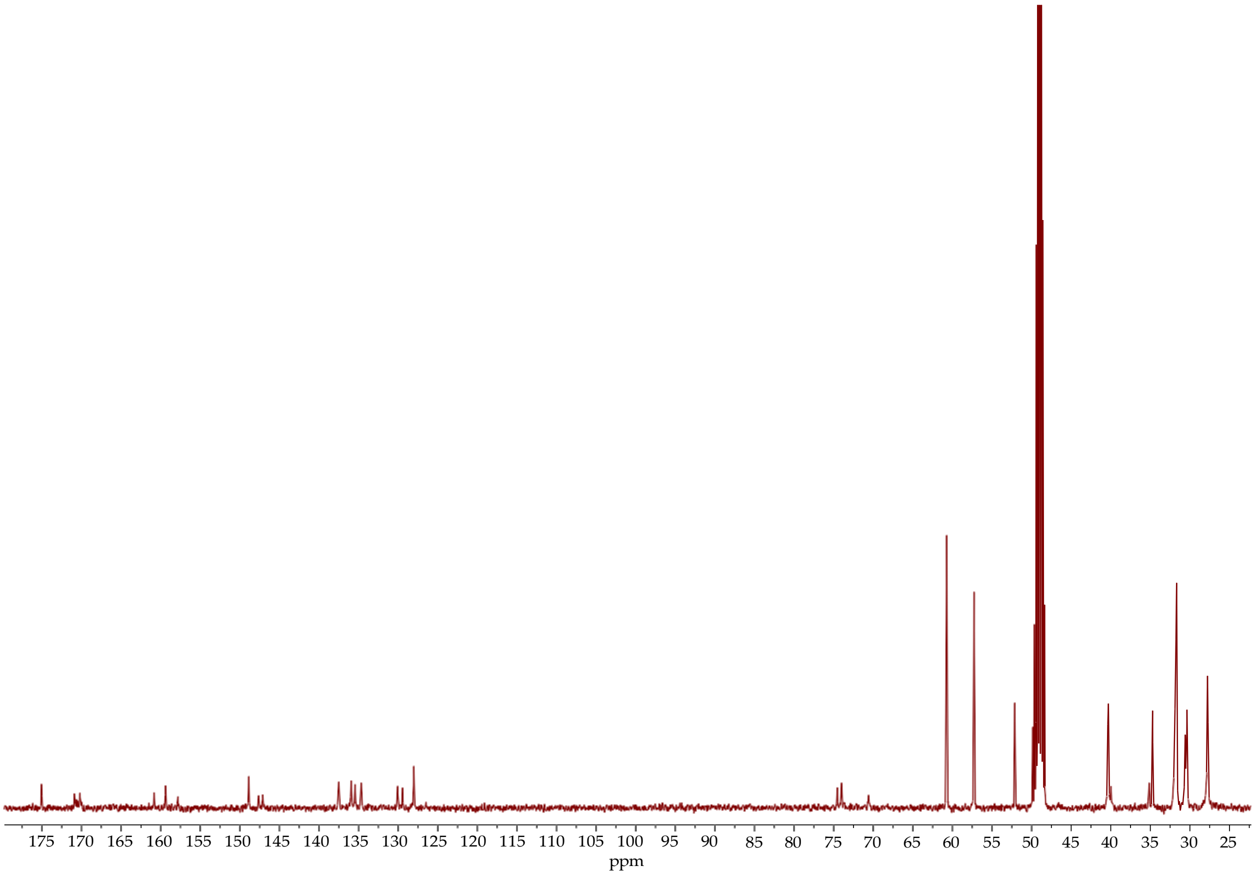

Figure S54. $^{13}C\{^{1}H\}$ NMR spectrum of **17**, CD_3OD, 298 K, 100 MHz

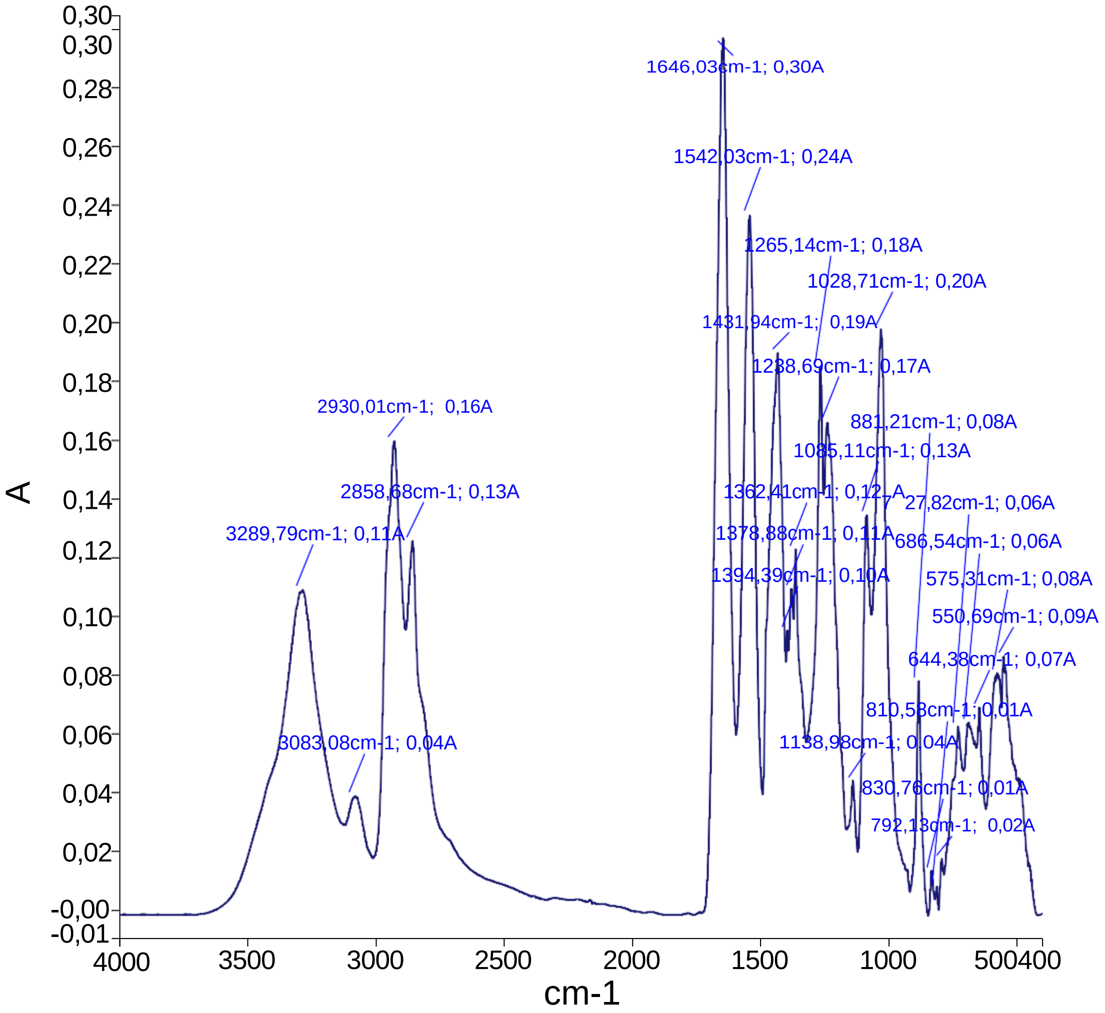

Figure S55. FTIR-ATR spectrum of **17**

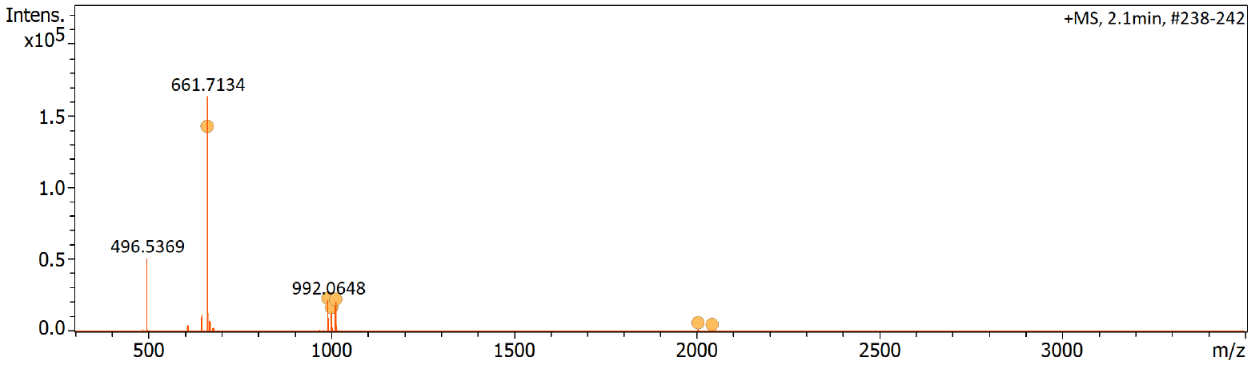

Figure S56. Mass spectrum (HR ESI) of **17**

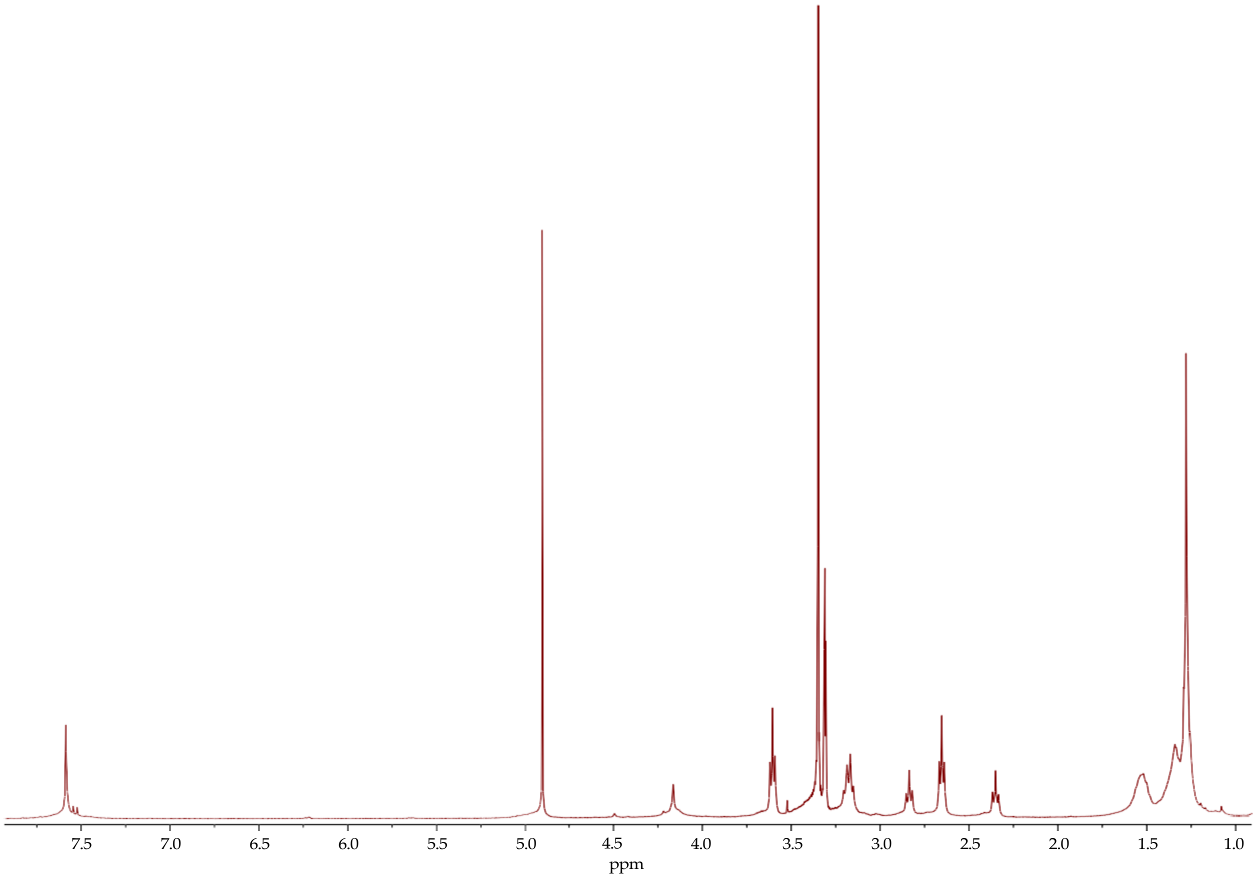

Figure S57. ^{1}H NMR spectrum of **18**, CD_3OD, 298 K, 400 MHz

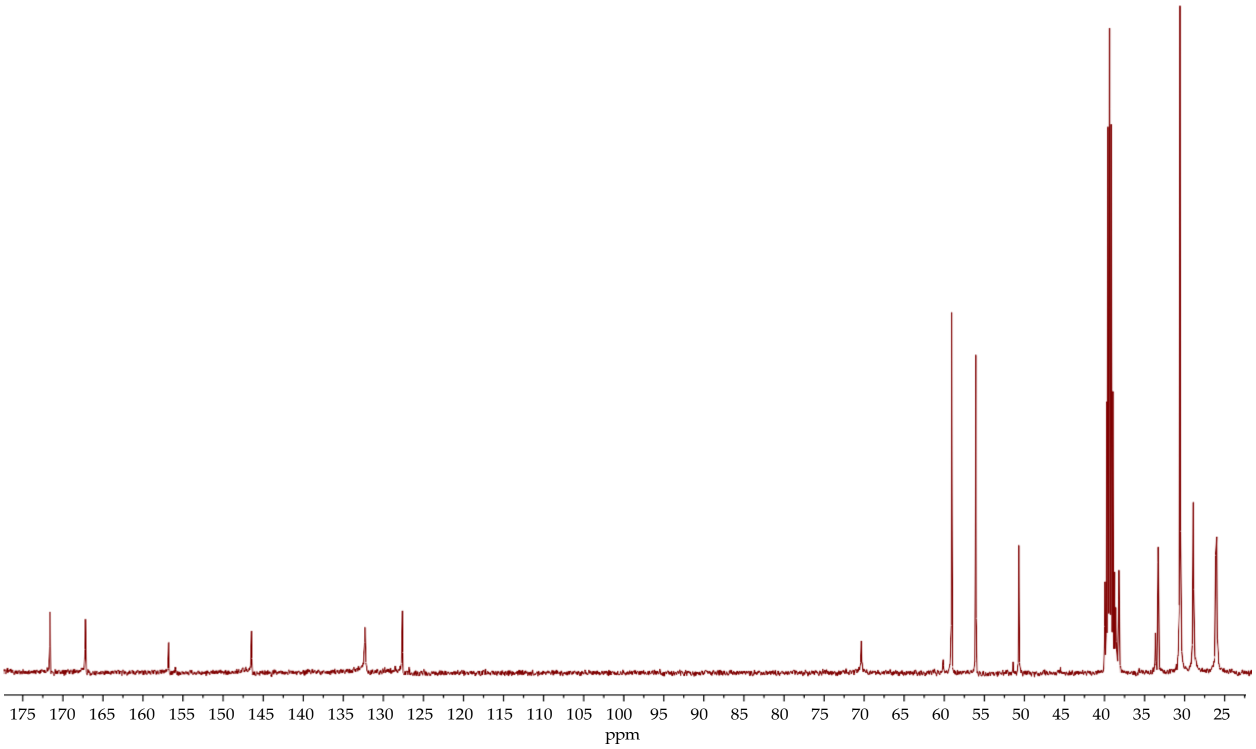

Figure S58. $^{13}C\{^{1}H\}$ NMR spectrum of **18**, CD_3OD, 298 K, 100 MHz

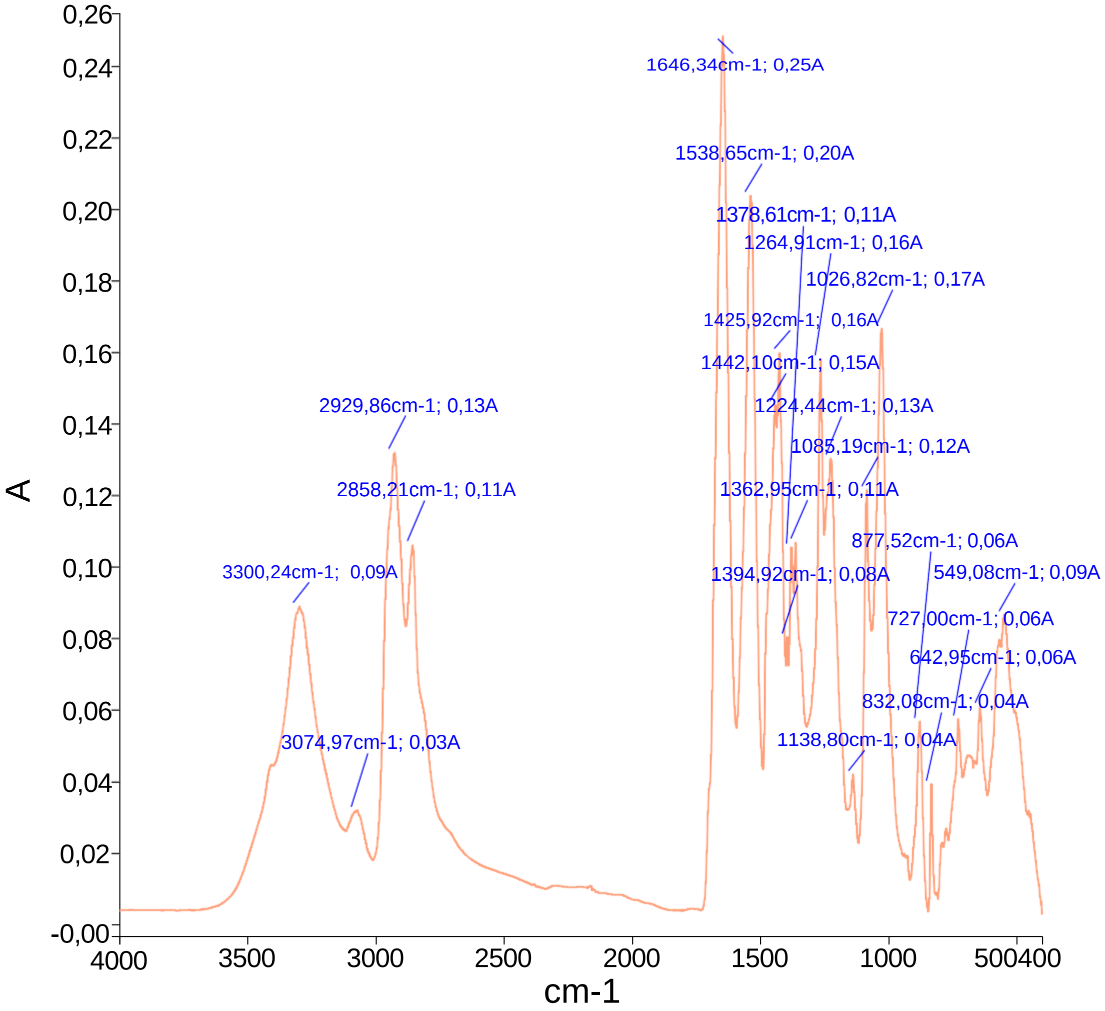

Figure S59. FTIR-ATR spectrum of **18**

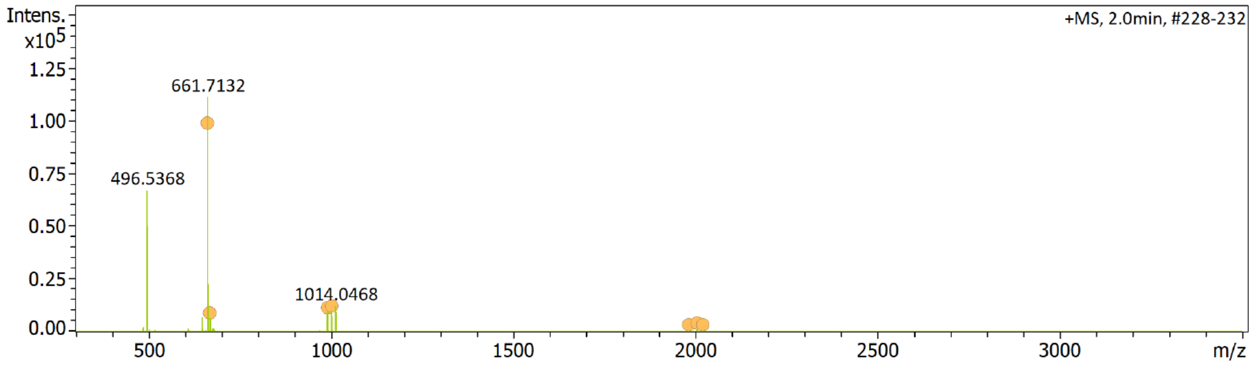

Figure S60. Mass spectrum (HR ESI) of **18**

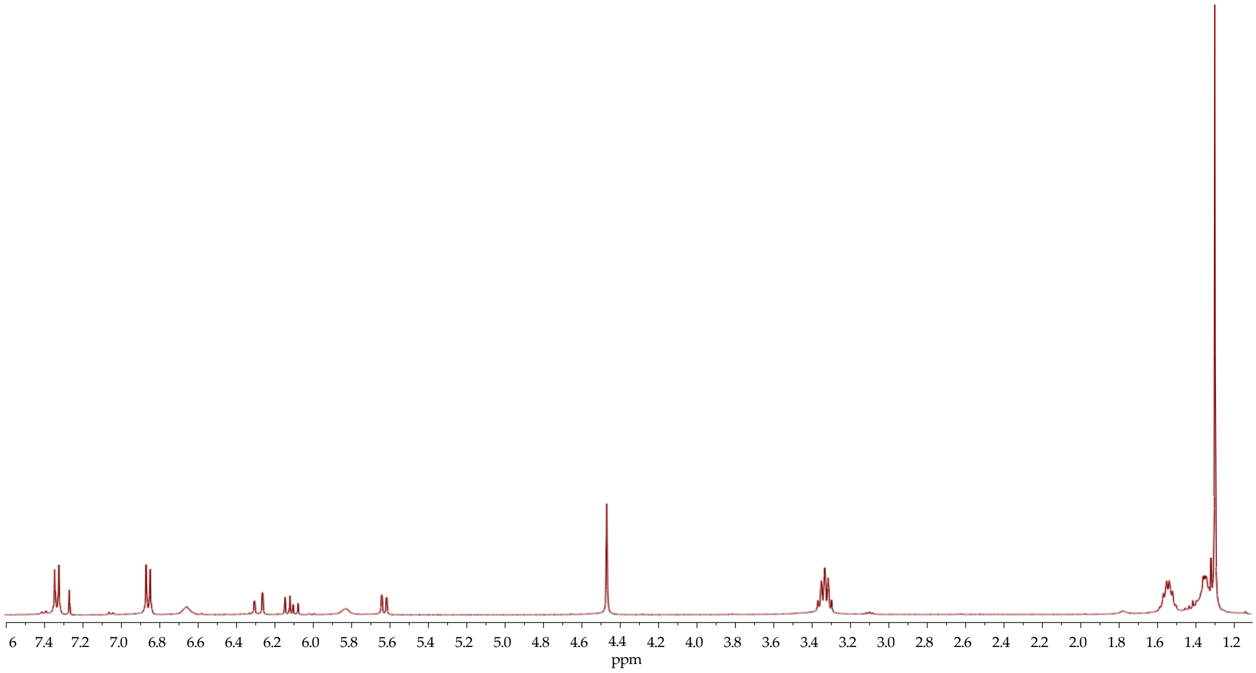

Figure S61. ^{1}H NMR spectrum of **20**, $CDCl_3$, 298 K, 400 MHz

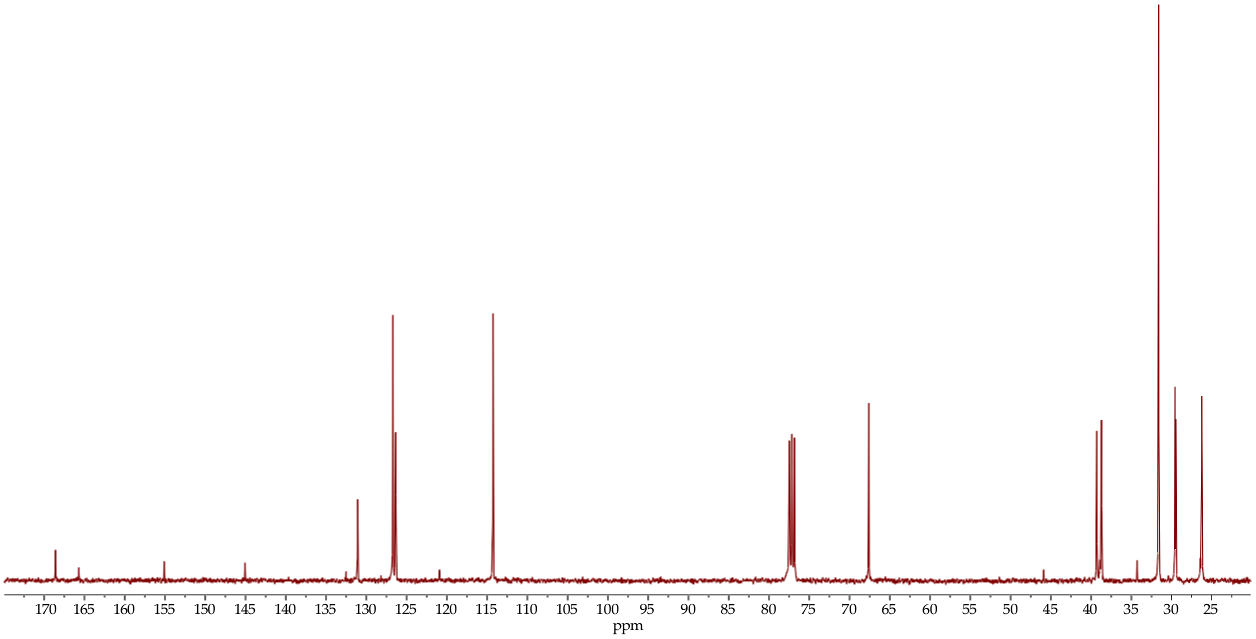

Figure S62. $^{13}C\{^{1}H\}$ NMR spectrum of **20**, $CDCl_3$, 298 K, 100 MHz

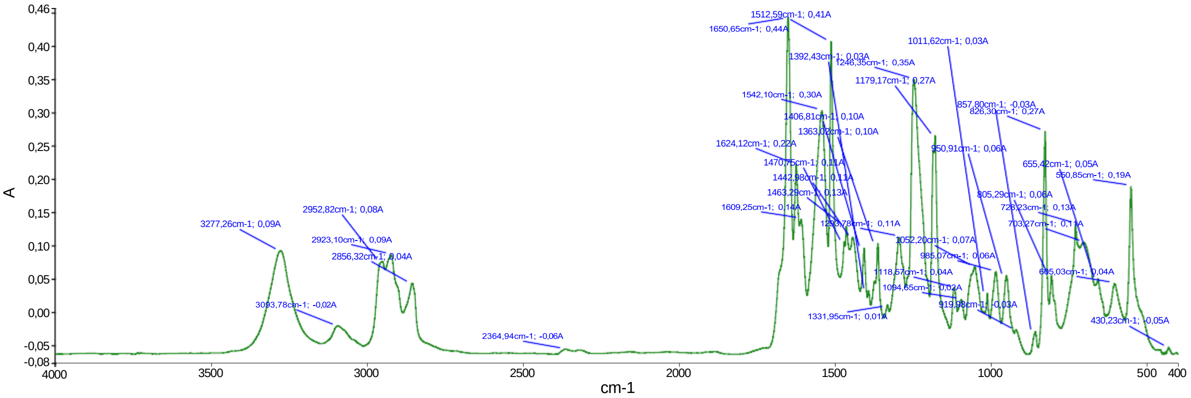

Figure S63. FTIR-ATR spectrum of **20**

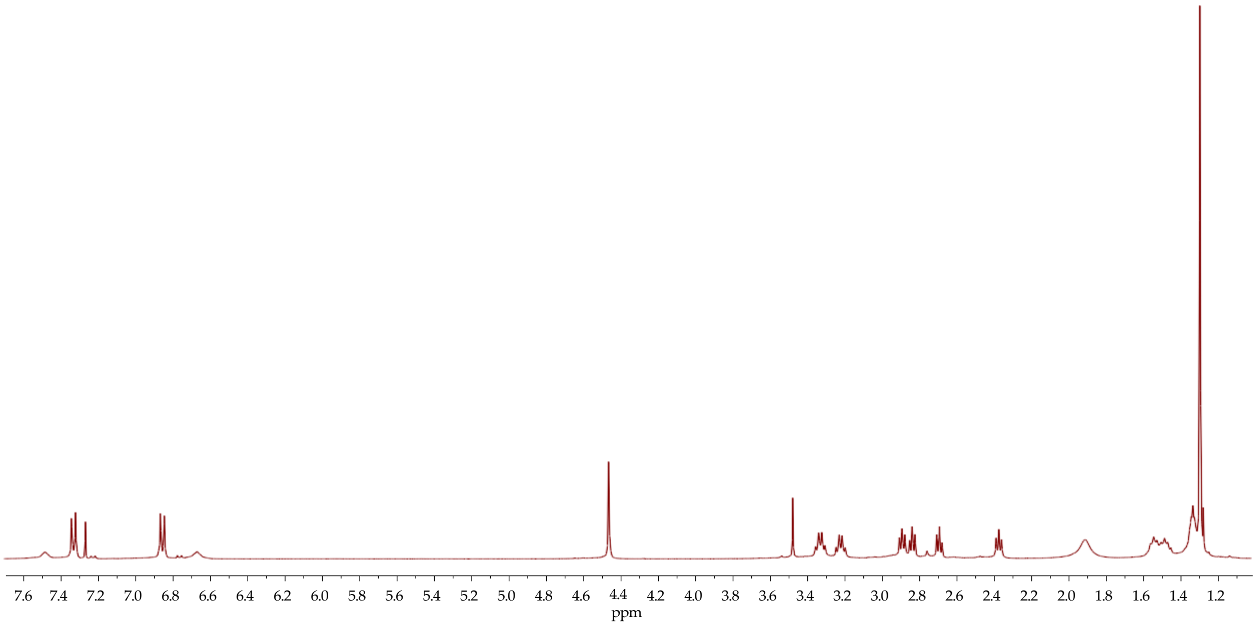

Figure S64. ^{1}H NMR spectrum of **21**, $CDCl_3$, 298 K, 400 MHz

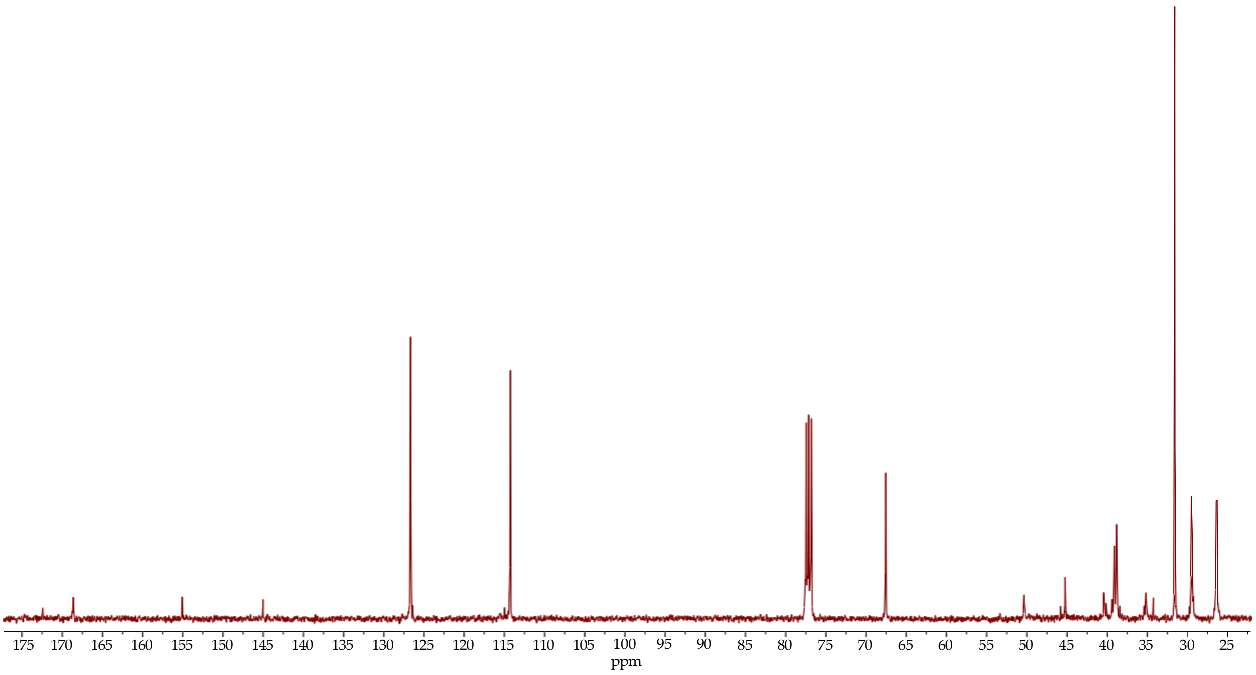

Figure S65. $^{13}C\{^{1}H\}$ NMR spectrum of **21**, $CDCl_3$, 298 K, 100 MHz

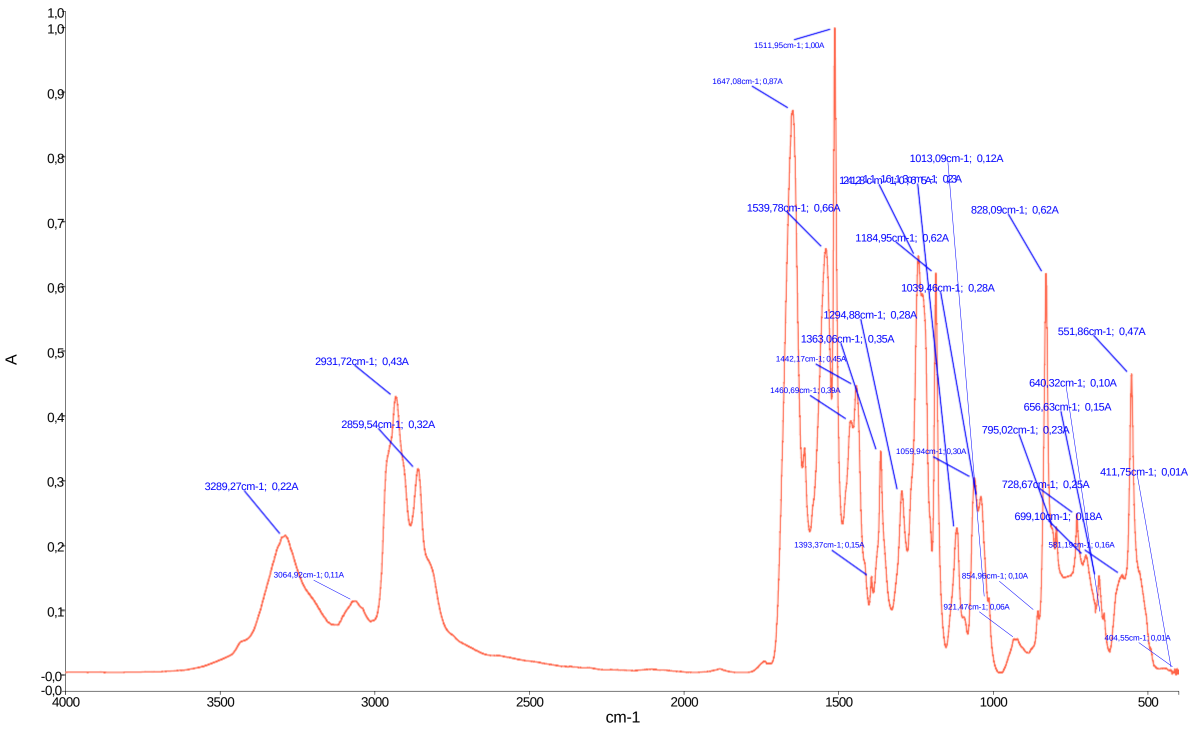

Figure S66. FTIR-ATR spectrum of **21**

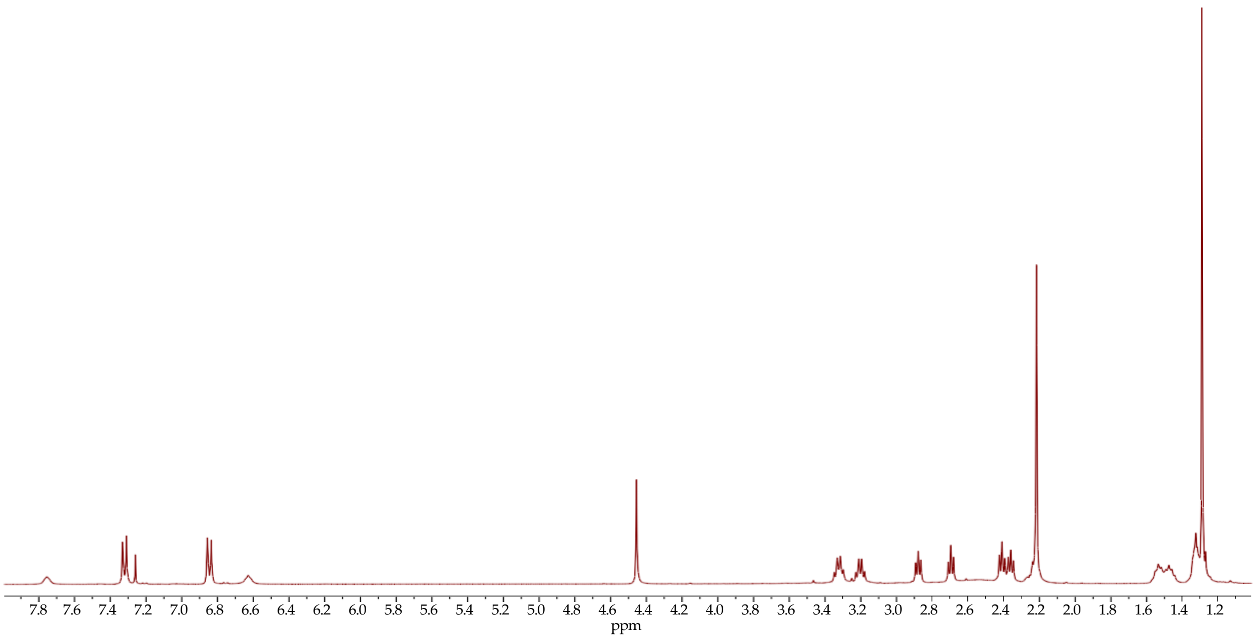

Figure S67. ^{1}H NMR spectrum of **22**, $CDCl_3$, 298 K, 400 MHz

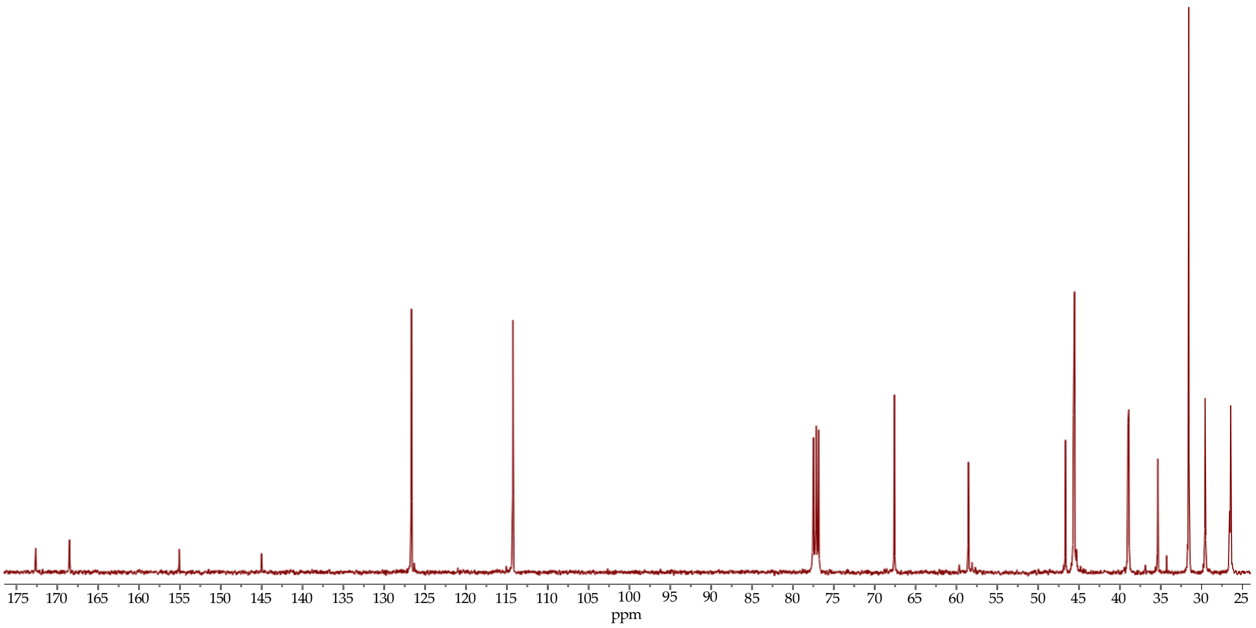

Figure S68. $^{13}C\{^{1}H\}$ NMR spectrum of **22**, $CDCl_3$, 298 K, 100 MHz

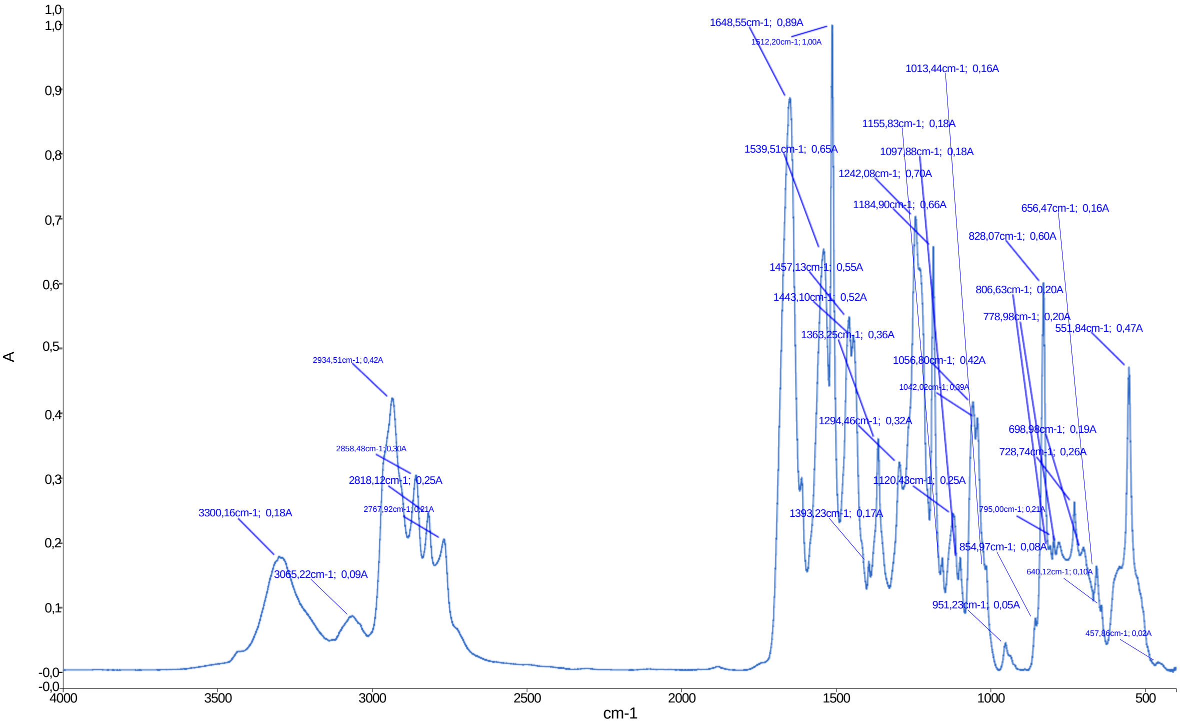

Figure S69. FTIR-ATR spectrum of **22**

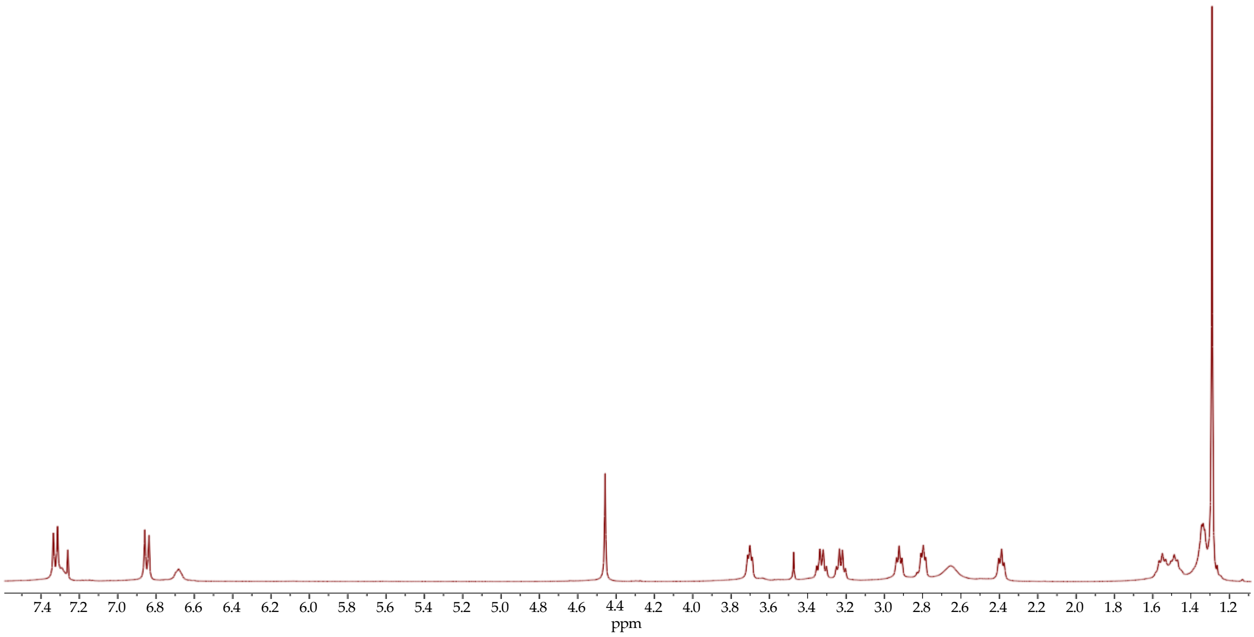

Figure S70. ^{1}H NMR spectrum of **23**, $CDCl_3$, 298 K, 400 MHz

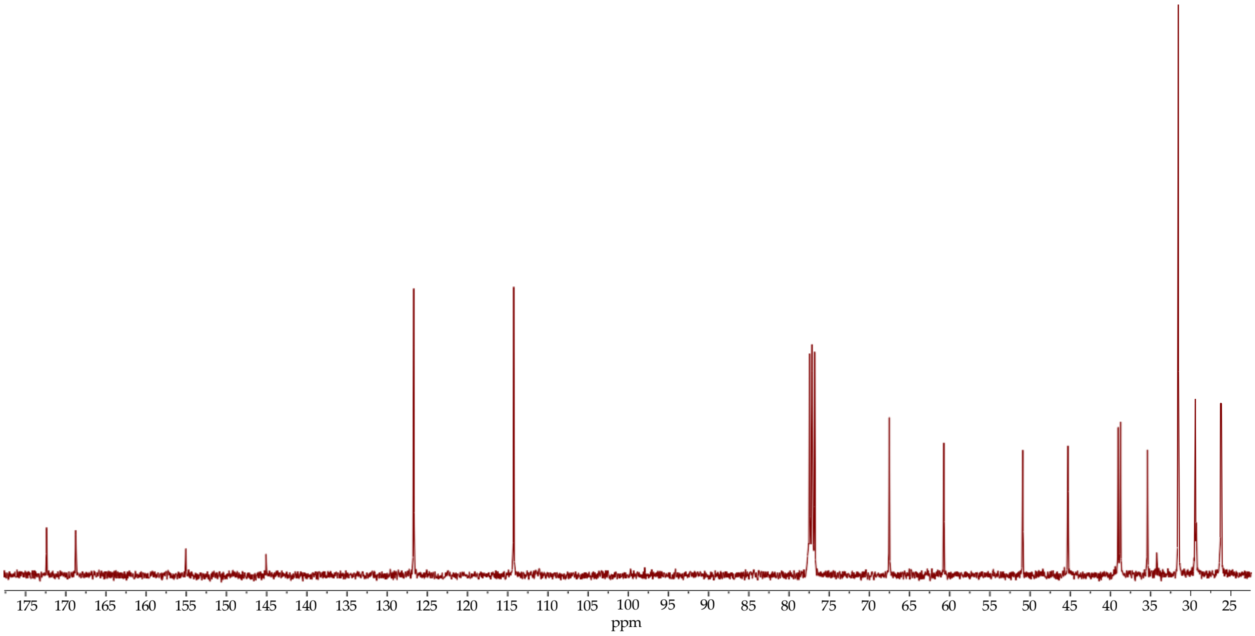

Figure S71. $^{13}C\{^1H\}$ NMR spectrum of **23**, $CDCl_3$, 298 K, 100 MHz

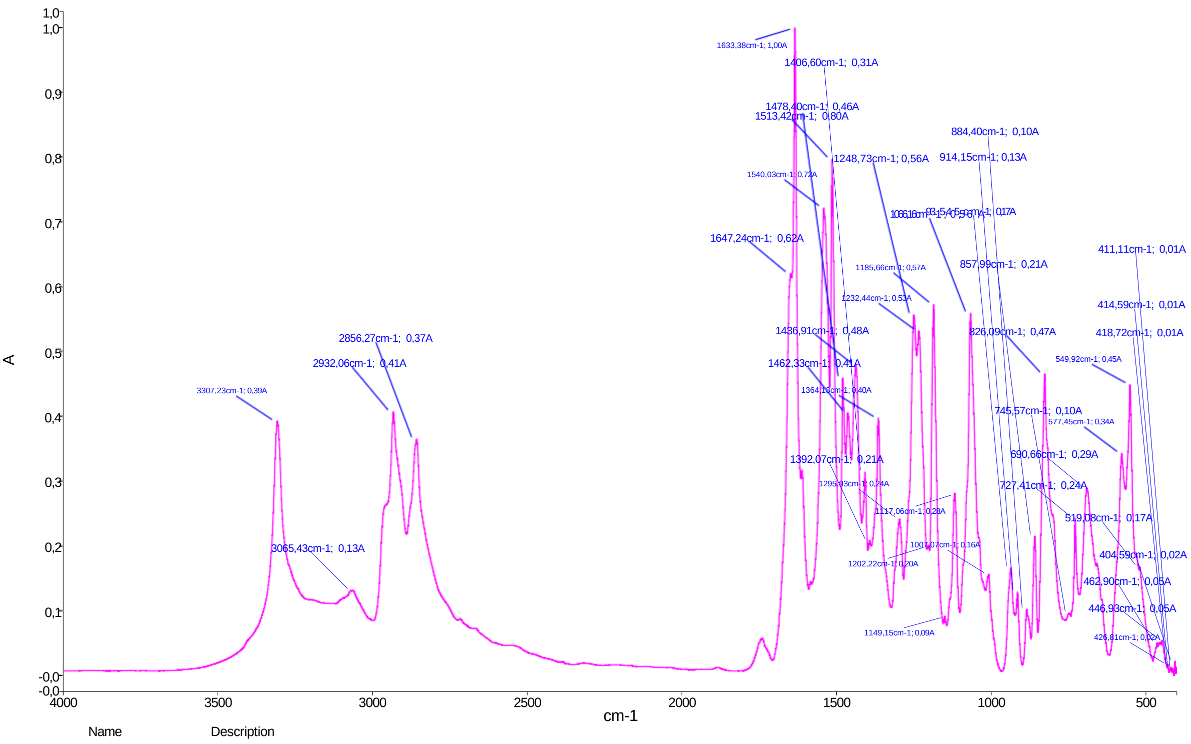

Figure S72. FTIR-ATR spectrum of **23**

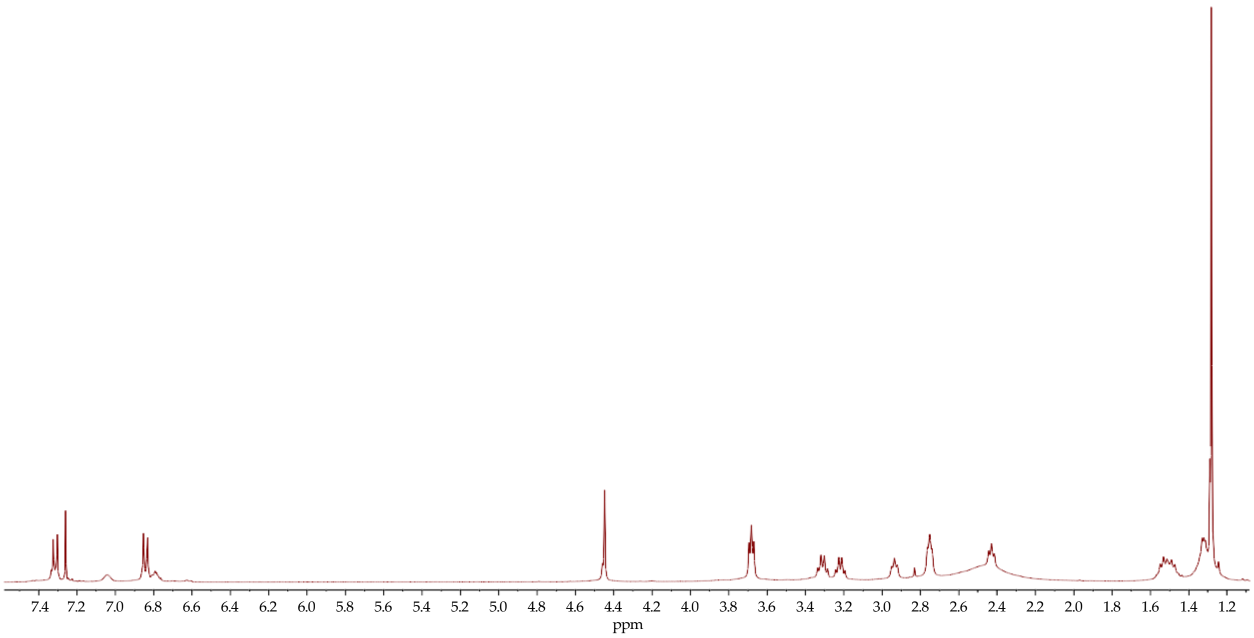

Figure S73. ^{1}H NMR spectrum of **24**, $CDCl_3$, 298 K, 400 MHz

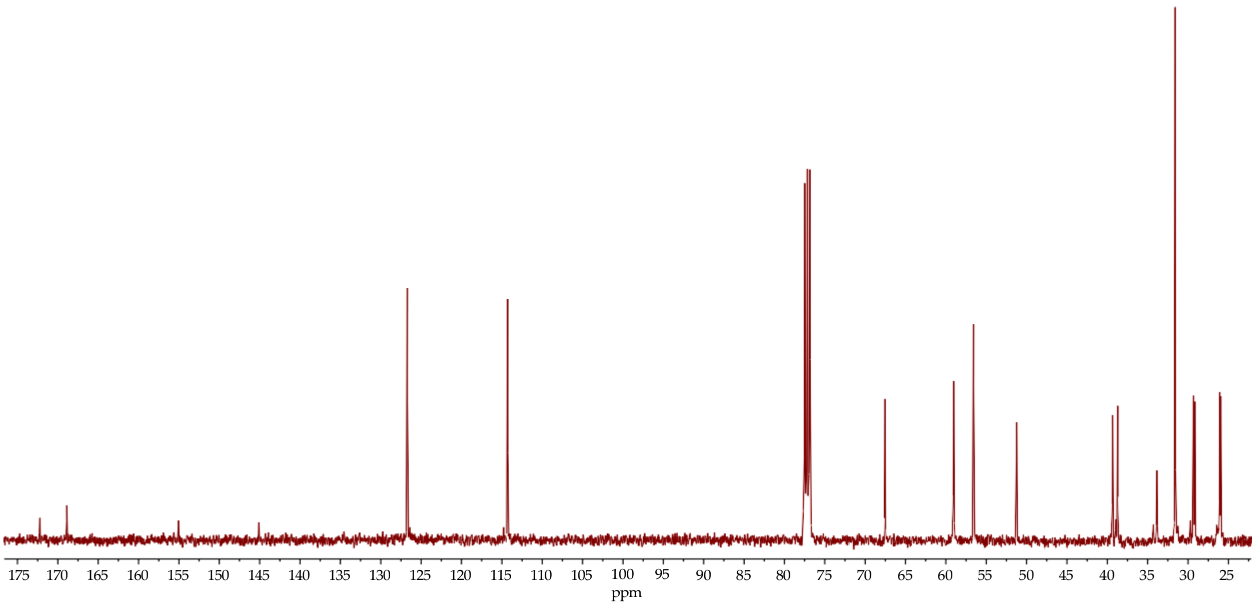

Figure S74. $^{13}C\{^1H\}$ NMR spectrum of **24**, $CDCl_3$, 298 K, 100 MHz

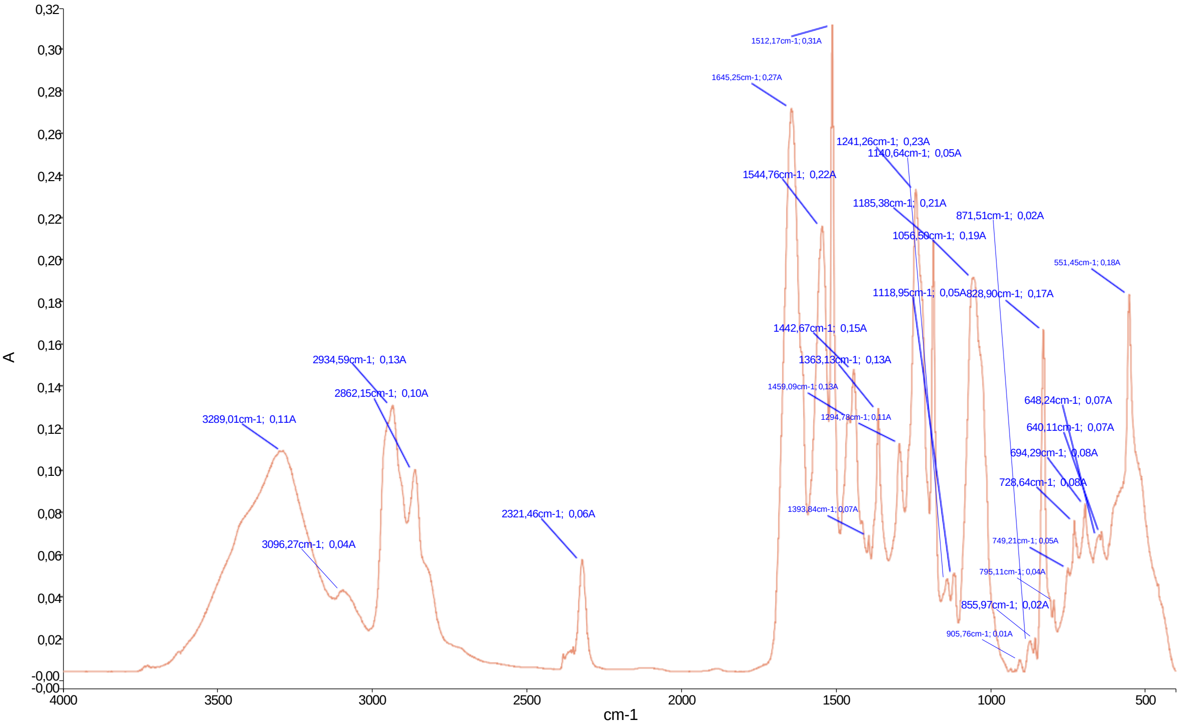

Figure S75. FTIR-ATR spectrum of **24**

2. Biological assay

2.1. Antimicrobial activity

The antimicrobial activity of the tested compounds was determined by serial micro-dilutions in 96-well plates. The dilutions were prepared in Müller-Hinton broth. Cultures of Gram-positive bacteria *Staphylococcus aureus* ATCC 6538 P FDA 209P (*S. aureus*), *Bacillus cereus* ATCC 10702 NCTC 8035 (*B. cereus*), *Enterococcus faecalis* ATCC 29212 (*E. faecalis*); Gram-negative bacteria *Escherichia coli* ATCC 25922 (*E. coli*), *Pseudomonas aeruginosa* ATCC 9027 (*P. aeruginosa*) were purchased from the State Collection of Pathogenic Microorganisms and Cell Cultures "GKPM-Obolensk". The concentration of bacteria in the experiment was equal to 3.0×10^5 CFU/mL. The results were recorded every 24 hours for 5 days. Bacterial cultures were incubated at 37 °C. The experiment was repeated three times. The dilutions of the compounds were prepared immediately in nutrient media; 5% DMSO was added for better solubility and the test strains were not inhibited at this concentration. The minimum inhibitory concentration (MIC) was defined as the minimum concentration of a compound that inhibits the growth of the corresponding test microorganism. The growth of bacteria as well as the absence of the growth due to the bacteriostatic action of a compounds were recorded. To determine the minimum bactericidal concentration (MBC), an aliquot of the test microorganism suspension was transferred to the agarized nutrient medium and incubated at 37 °C. MBCs represent the minimum concentrations, at which microbial colonies were absent indicating they were killed with the efficiency of > 99.9% [S1].

2.2. Cytotoxic Assay

The cytotoxic effect on cells was determined using the colorimetric method of cell proliferation - the MTT test. NADP-H-dependent cellular oxidoreductase enzymes can, under certain conditions, reflect the number of viable cells. These enzymes are able to reduce the tetrazolium dye (MTT) - 3-(4,5-dimethylthiazol-2-yl)-2,5-diphenyl-tetrazolium bromide to insoluble blue-violet formazan, which crystallizes inside the cell. The amount of formazan formed is proportional to the number of cells with active metabolism. Chang liver cell line (Human liver cells) from collection N. F. Gamaleya Research Center of Epidemiology and Microbiology was used in the experiments. The cells were cultured in a standard Eagle's nutrient medium manufactured at the Chumakov Institute of Poliomyelitis and Virus Encephalitis (PanEco company) and supplemented with 10% fetal calf serum and 1% nonessential amino acids. Cells were seeded on a 96-well Nunc plate at a concentration of 5×10^3 cells per well in a volume of 100 μL of medium and cultured in a CO_2 incubator at 37 °C until a monolayer was formed. Then the nutrient medium was removed and 100 μl of solutions of the test drug in the given dilutions were added to the wells, which were prepared directly in the nutrient medium with the addition of 5% DMSO to improve solubility. After 24 h of incubation of the cells with the tested compounds, the nutrient medium was removed from the plates and 100 μL of the nutrient medium without serum with MTT at a concentration of 0.5 mg/mL was added and incubated for 4 h at 3 7°C. Formazan crystals were added 100 μL of DMSO to each well. Optical density was recorded at 540 nm on an Invitrologic microplate reader (Russia). The value of IC_{50} (the drug concentration that inhibits cell growth by 50%) was calculated using Quest Graph ™ IC50 Calculator (https://www.aatbio.com/tools/ic50-calculator). The experiments for all compounds were repeated three times [S2].

3. DLS data

To prepare stock lipid suspension, 20.5 mg of 2-oleoyl-*sn*-glycero-3-phospho-(1′-*rac*-glycerol) sodium salt (POPG) was placed in flat bottom beaker and dissolved in 1000 μL of chloroform. The solution was evaporated overnight under vacuum to obtain a thin lipid film. Then 380 μL of buffer solution (50 mM Tris-HCl, 150 mM NaCl, pH 7.4) was added, and the resulting mixture stirred until formation of crude suspension. The suspension was subjected to 5 cycles of freezing in liquid nitrogen and thawing at 60 °C, followed by 19-fold extrusion with Avanti Mini-Extruder (Avanti Polar Lipids, Birmingham, AL, USA) through a 100 nm polycarbonate filter (Avanti 610005) at 50 °C. As a result, unilamellar vesicles with an average diameter of 100 nm were obtained. The concentration of a lipid in stock suspension was 70 mM.

The values of the hydrodynamic diameter and the electrokinetic potential (zeta potential) of the POPG vesicles were determined by a Zetasizer Nano ZS instrument (Worcestershire, UK) at 37 °C. POPG vesicles (1×10^{-4} M) were prepared in TRIS buffer (50 mM Tris-HCl, 150 mM NaCl, pH = 7.4) from the stock suspension. Supramolecular systems [POPG + macrocycle] were prepared by addition of the solutions of the compounds to the resulting vesicles in the molar ratio [POPG : macrocycle] = 1:0.1 and [POPG : macrocycle] = 1:1. The determination of the particle size was carried out in 1 h after the sample preparation. To assess the kinetic stability of the systems, the measurements were also carried out under similar conditions after 24 h.

Table S1. The size distributions (intensity) of systems POPG (1×10^{-4} M) and POPG/Macrocycle (Tris-HCl buffer, pH = 7.4, 50 mM, 150 mM NaCl).

**Supramolecular system**		**Size Distribution (intensity)**
POPG		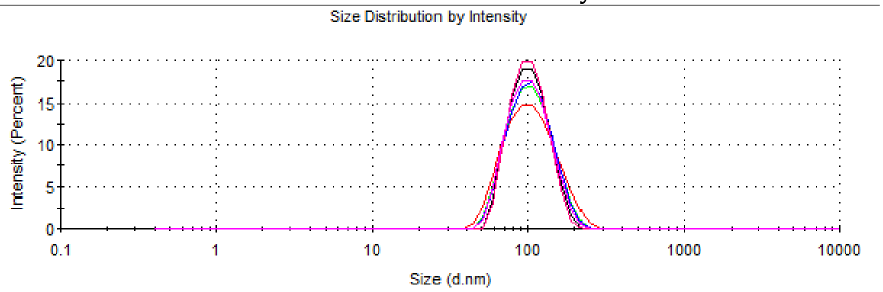
POPG + **10** (*cone*)	1:0.1	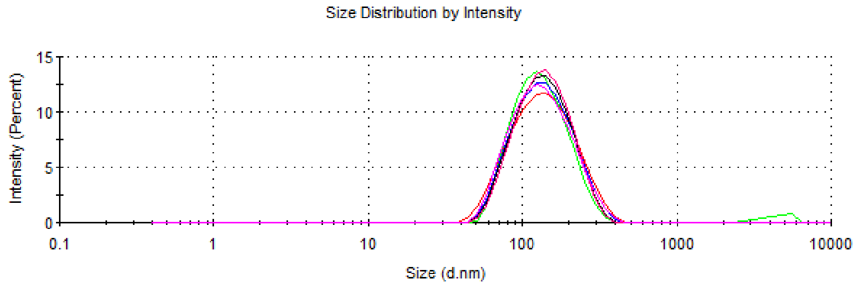
	1:1	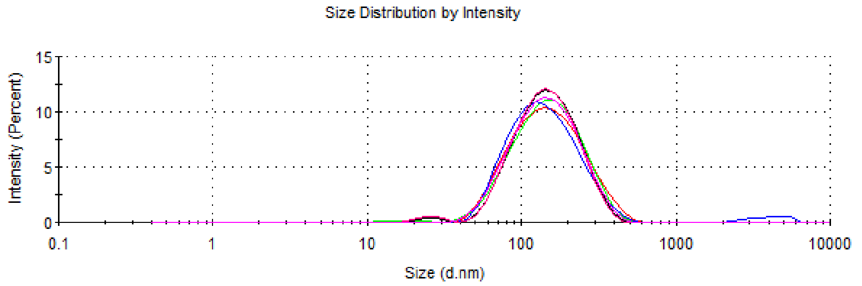

POPG + **11** (*partial cone*)	1:0.1	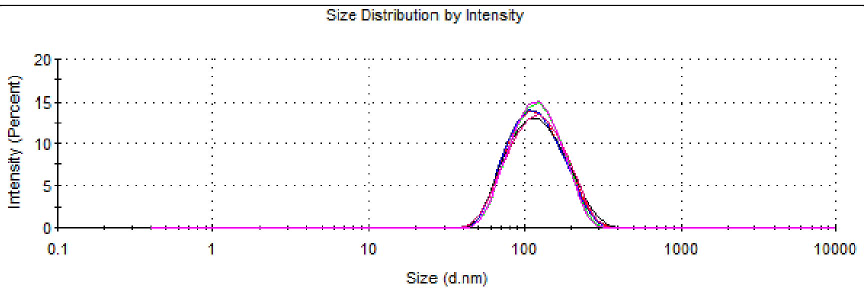
	1:1	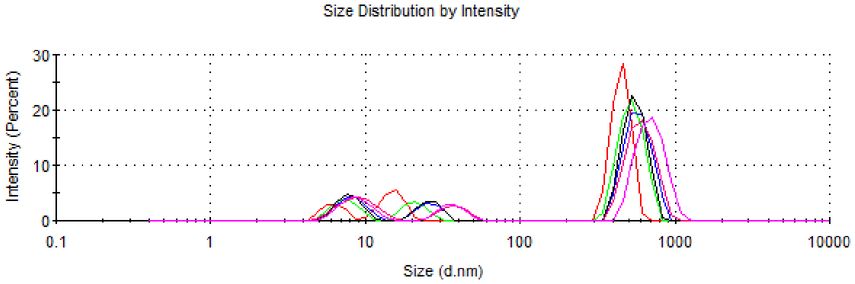
POPG + **12** (*1,3-alternate*)	1:0.1	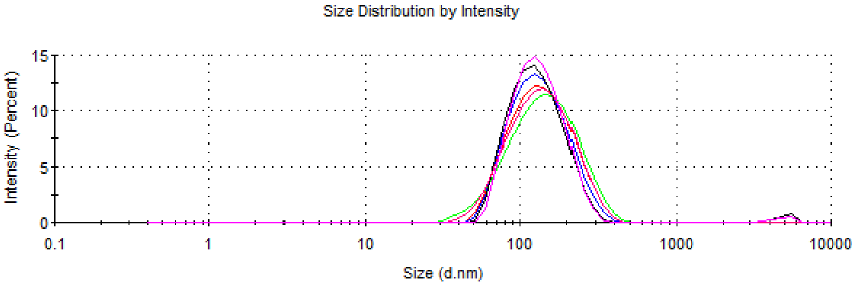
	1:1	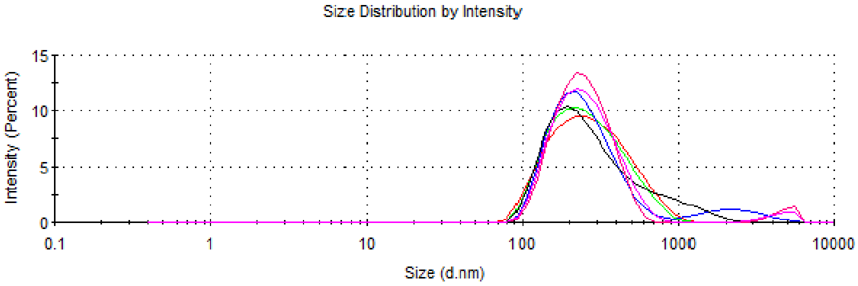

Table S2. Aggregation data (by intensity) of compounds **10–12** (Tris-HCl buffer, pH = 7.4, 50 mM, 150 mM NaCl).

Compound	Concentration (M)	D, nm	PDI
**10** (*cone*)	1×10^{-4}	684 ± 67	0.59
	1×10^{-5}	392 ± 14	0.32
**11** (*partial cone*)	1×10^{-4}	1232 ± 130	0.69
	1×10^{-5}	517 ± 26	0.40
**12** (*1,3-altenate*)	1×10^{-4}	1217 ± 111	0.70
	1×10^{-5}	746 ± 45	0.47

Table S3. Values (MW, miLogP, HBA, HBD, TPSA)[a] and solubility data for compounds **7–18** and **21–24**.

Compound	Terminal fragment	MW	miLogP	TPSA	HBA	HBD	solubility[b]
**7** (*cone*)<br>**8** (*partial cone*)<br>**9** (*1,3-alternate*)	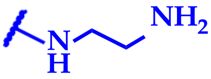	1803	8.74	422	28	20	H_2O, MeOH, EtOH, *i*PrOH, DMSO
**21** (monomer)		449	1.97	105	7	5	H_2O, MeOH, EtOH, *i*PrOH, DMSO
**10** (*cone*)<br>**11** (*partial cone*)<br>**12** (*1,3-alternate*)	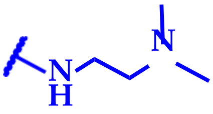	1915	9.95	331	28	12	H_2O, PBS, MeOH, EtOH, *i*PrOH, DMSO
**22** (monomer)		421	3.20	83	7	3	H_2O, PBS, MeOH, EtOH, *i*PrOH, DMSO
**13** (*cone*)<br>**14** (*partial cone*)<br>**15** (*1,3-alternate*)	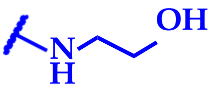	1807	9.47	399	28	16	H_2O, PBS, MeOH, EtOH, *i*PrOH, DMSO
**23** (monomer)		422	2.54	100	7	4	H_2O, PBS, MeOH, EtOH, *i*PrOH, DMSO
**16** (*cone*)<br>**17** (*partial cone*)<br>**18** (*1,3-alternate*)	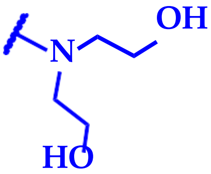	1983	9.44	445	32	16	H_2O, PBS, MeOH, EtOH, *i*PrOH, DMSO
**24** (monomer)		466	2.50	111	8	4	H_2O, PBS, MeOH, EtOH, *i*PrOH, DMSO

[a]MW, molecular weight; miLogP, logarithm of the octanol–water partition coefficient; HBA, hydrogen bond acceptor atoms; HBD, hydrogen bond donor atoms; TPSA, topological polar surface area values were calculated using an online platform http://www.molinspiration.com/cgi-bin/properties. [b]The solvents listed provide complete solubility of the compounds.

References

S1. Voloshina, A.D.; Gumerova, S.K.; Sapunova, A.S.; Kulik, N.V.; Mirgorodskaya, A.B.; Kotenko, A.A.; Prokopyeva, T.M.; Mikhailov, V.A.; Zakharova, L.Y.; Sinyashin, O.G. The Structure – Activity Correlation in the Family of Dicationic Imidazolium Surfactants: Antimicrobial Properties and Cytotoxic Effect. *Biochim. Biophys. Acta, Gen. Subj.* **2020**, *1864*, 129728. doi:10.1016/j.bbagen.2020.129728

S2. Agarkov, A.S.; Nefedova, A.A.; Gabitova, E.R.; Mingazhetdinova, D.O.; Ovsyannikov, A.S.; Islamov, D.R.; Amerhanova, S.K.; Lyubina, A.P.; Voloshina, A.D.; Litvinov, I.A.; et al. (2-Hydroxy-3-Methoxybenzylidene)Thiazolo[3,2-a]Pyrimidines: Synthesis, Self-Assembly in the Crystalline Phase and Cytotoxic Activity. *Int. J. Mol. Sci.* **2023**, *24*, 2084. doi:10.3390/ijms24032084
